# Heterogeneity of Inflammatory and Cytokine Networks in Chronic Plaque Psoriasis

**DOI:** 10.1371/journal.pone.0034594

**Published:** 2012-03-29

**Authors:** William R. Swindell, Xianying Xing, Philip E. Stuart, Cynthia S. Chen, Abhishek Aphale, Rajan P. Nair, John J. Voorhees, James T. Elder, Andrew Johnston, Johann E. Gudjonsson

**Affiliations:** 1 Department of Genetics, Harvard Medical School, Boston, Massachusetts, United States of America; 2 Department of Dermatology, University of Michigan School of Medicine, Ann Arbor, Michigan, United States of America; BSRC ‘Alexander FLEMING’, Greece

## Abstract

The clinical features of psoriasis, characterized by sharply demarcated scaly erythematous plaques, are typically so distinctive that a diagnosis can easily be made on these grounds alone. However, there is great variability in treatment response between individual patients, and this may reflect heterogeneity of inflammatory networks driving the disease. In this study, whole-genome transcriptional profiling was used to characterize inflammatory and cytokine networks in 62 lesional skin samples obtained from patients with stable chronic plaque psoriasis. We were able to stratify lesions according to their inflammatory gene expression signatures, identifying those associated with strong (37% of patients), moderate (39%) and weak inflammatory infiltrates (24%). Additionally, we identified differences in cytokine signatures with heightened cytokine-response patterns in one sub-group of lesions (IL-13-strong; 50%) and attenuation of these patterns in a second sub-group (IL-13-weak; 50%). These sub-groups correlated with the composition of the inflammatory infiltrate, but were only weakly associated with increased risk allele frequency at some psoriasis susceptibility loci (e.g., *REL*, *TRAF3IP2* and *NOS2*). Our findings highlight variable points in the inflammatory and cytokine networks known to drive chronic plaque psoriasis. Such heterogeneous aspects may shape clinical course and treatment responses, and can provide avenues for development of personalized treatments.

## Introduction

Psoriasis is a chronic inflammatory skin disease characterized by marked proliferation of keratinocytes leading to pronounced epidermal hyperplasia, elongation of rete ridges and hyperkeratosis. The most common form of psoriasis, chronic plaque psoriasis (*Psoriasis vulgaris*), involves relatively stable occurrence and progression of sharply demarcated lesions, usually on the trunk and extremities, which share a combination of trademark histological features, including tortuous and dilated dermal capillaries, loss of the epidermal granular layer, and accumulation of neutrophils beneath parakeratotic scale [1], [2]. Severity and progression of psoriasis, as well as its response to treatment, is variable among patients [3]–[5]. This may reflect heterogeneity in patient-specific inflammatory networks, although at present, factors that account for variability in disease presentation, progression and treatment response are not well understood [6], [7].

The transcriptome of psoriasis lesions has been evaluated using high-throughput methods, and in principle, such information could provide an objective basis for identifying molecular sub-types of psoriasis, while providing insight into factors that account for variability among patients [8]–[15]. The most widely held model of psoriasis pathogenesis proposes that keratinocyte hyper-proliferation is triggered by cutaneous lymphocyte infiltration, activation and differentiation of inflammatory cells, including T-cells, macrophages, and dendritic cells, and that these events generate a localized cytokine environment that both sustains and reinforces the pathogenic cascade [16]. Key factors that underlie this process, i.e., the presence of inflammatory cells and cytokines, can be characterized and modeled by algorithms applied to genome-wide expression patterns [13], [17], [18]. Objective and biologically meaningful differentiation among individual patients may therefore be achieved on the basis of functional genomic data and development of computational approaches. Previous analyses of the psoriasis transcriptome have identified genes differentially expressed between lesional and non-lesional skin, leading to the identification of numerous genes for which expression is robustly associated with psoriatic lesions [8]–[15]. This analytical approach, however, is implicitly oriented towards those features of genome-wide expression profiles that exhibit least variability among lesional skin samples from patients, filtering out heterogeneous expression patterns and their inter-patient variability.

In this study, we have analyzed genome-wide expression patterns in psoriasis from a new perspective, with the distinct aim of characterizing heterogeneity among lesional skin samples in terms of biologically meaningful patterns embedded within transcriptome data. For this purpose, we utilized an algorithm developed for the calculation of “inflammation profiles” from microarray data, which aims to explain expression differences between lesional and non-lesional skin in terms of shifts in the composition of cellular infiltrate [18]. In addition, sets of cytokine-responsive transcripts, identified from experiments performed with cultured keratinocytes, are used to analyze differences in cytokine activity among psoriasis lesions [19]. Based on these patterns, we characterize the spectrum of molecular phenotypes associated with the clinical presentation of chronic plaque psoriasis. These analytical methods can be readily applied on a larger scale, and we anticipate that this will facilitate development of personalized treatment regimes [6], [20], [21].

## Results

### Psoriasis lesions from 62 patients can be classified into three sub-groups (weak, moderate and strong inflammatory infiltrate signatures)

**Figure 1:**
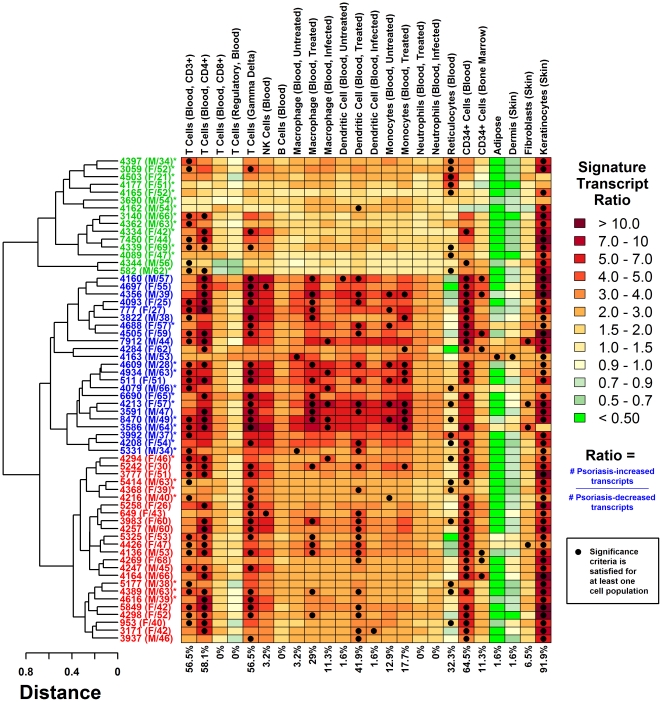
Sub-division of psoriasis lesions into strong, moderate and weak inflammatory groups based on genome-wide expression profiles. The heatmap displays intensity of inflammatory gene expression patterns in psoriatic plaques obtained from 62 subjects (rows) with respect to 24 different cell types (columns; immunocytes and dermal cells). Strong inflammatory signatures are denoted by blue subject labels (23/62 subjects), moderate signatures by red labels (24/62) and weak signatures by green labels (15/62). Lesional (PP) and non-lesional (PN) skin from each subject was analyzed by microarray to identify PP-increased and PP-decreased transcripts. Chart colors denote the behavior of 1000 “signature transcripts” associated with each cell population (see scale and Methods). Percentage values (bottom) denote the total fraction of subjects with a significant bias towards PP-increased expression among signature transcripts (black dots). Subject labels include a patient identifier with sex (M or F) and age. Asterisk symbols denote subjects with IL-13-weak gene expression signatures (see text and Figure 2). An expanded version of this figure is provided as supplemental material (Figure S1).

We evaluated genome-wide expression patterns in lesional (PP) and non-lesional (PN) skin biopsies obtained from 62 patients with chronic plaque psoriasis. For each of 54,675 probe sets (Affymetrix Human Genome 133 Plus 2.0 array), we calculated the difference in gene expression between paired PP and PN skin from individual patients. We then calculated an “inflammation profile” for each patient, based upon PP versus PN expression level differences for “signature transcripts” that are highly expressed within specific cell types in skin or the immune system (Figures 1 and S1) [18]. Among the 62 patients, the most consistent feature was elevated expression of keratinocyte-associated transcripts in PP skin compared with PN skin (significant in 91.9% of subjects; see Figures 1 and S1). Additionally, in PP skin from most subjects, we noted elevated expression of transcripts associated with CD34+ cells (64.5%), CD4+ T-cells (58.1%), γδ T-cells (56.5%) and CD3+ T-cells (56.5%) (Figures 1 and S1). These consistent features of inflammation profiles correspond well with known histological properties of psoriasis lesions [1], and we have used immunohistochemistry to confirm the presence of these and other cell types in PP samples (Figure S2). We did not detect statistically significant outliers among the 62 subjects (Grubb's test, P≥0.45; Figure S3). However, we noted substantial heterogeneity with respect to some cell types (e.g., monocytes, macrophages and dendritic cells), with significant patterns detected in only a fraction (<50%) of the patient cohort (Figures 1 and S1).

The inflammatory signatures from each patient were further analyzed by hierarchical cluster analysis. The clustering solution suggested at least three sub-groups, including 23 patients with strong inflammatory infiltrate signatures (blue labels; Figures 1, S1 and S4), 24 subjects with moderate signatures (red labels; Figures 1, S1 and S4), and 15 subjects with weak signatures (green labels; Figures 1, S1 and S4). The exact number of sub-groups detected by this approach depends upon the “height” or inter-signature distance at which the dendrogram tree is “cut” (Figures 1 and S1). As a guide for choosing this height, we analyzed an independent dataset from the study of Yao et al. [11], which consisted of PP and PN expression profiles from 28 patients (Figure S5) [11]. These data also suggested the presence of three patient sub-groupings with characteristics resembling those of the strong, moderate and weak inflammatory groups identified in our dataset of 62 patients. This indicated that a three-group partitioning of inflammatory patterns can be replicated in an external patient cohort, and along these lines, we chose a dendrogram “cut” that yielded three sub-groups (Figure S5).

The 23 patients with strong inflammatory signatures were characterized by robust trends among cell types consistently significant in all inflammation profiles (e.g., keratinocytes, γδ T-cells, CD4+ T-cells, CD3+ T-cells and CD34+ cells), but in addition, exhibited strong trends with respect to NK-cells, monocytes, macrophages, dendritic cells and bone-marrow progenitor cells (Figures 1, S1 and S4). The 24 patients with moderate inflammatory signatures, in contrast, exhibited weaker trends with respect to this latter group of cell types (Figures 1, S1 and S4). There was a strong distinction between the 15 weak inflammatory signatures and those from the other two groups (Figures 1, S1 and S4). Among patients with weak signatures, even the most consistent features among subjects were notably attenuated (e.g., CD4+ T-cells, γδ T-cells, NK-cells, CD34+ cells and progenitor cells), and there was quantitatively depressed elevation of transcripts associated with cell types from the monocyte-macrophage and monocyte-DC lineages (Figures 1, S1 and S4).

Infiltrating immune cells drive keratinocyte responses in psoriasis [1], [22], [23], but there was no strong tendency for weaker inflammatory signatures to associate with attenuated keratinocyte responses. However, the proportion of keratinocyte signature transcripts elevated in PP samples relative to PN samples varied from 64.3% (95% confidence interval: 61.3%–67.3%) to 94.0% (92.5%–95.5%) (Figure S6A). Among CD4+ T-cell and DC-associated transcripts, the proportion of PP-elevated transcripts varied from 55% to 90%, with weak and moderately strong inflammatory signatures mainly present in the lower tail of the distribution (Figures S6B and S6C).

### Psoriasis lesions from 62 patients can be classified into two sub-groups (IL-13-strong and IL-13-weak) based upon expression patterns of cytokine-responsive transcripts

**Figure 2:**
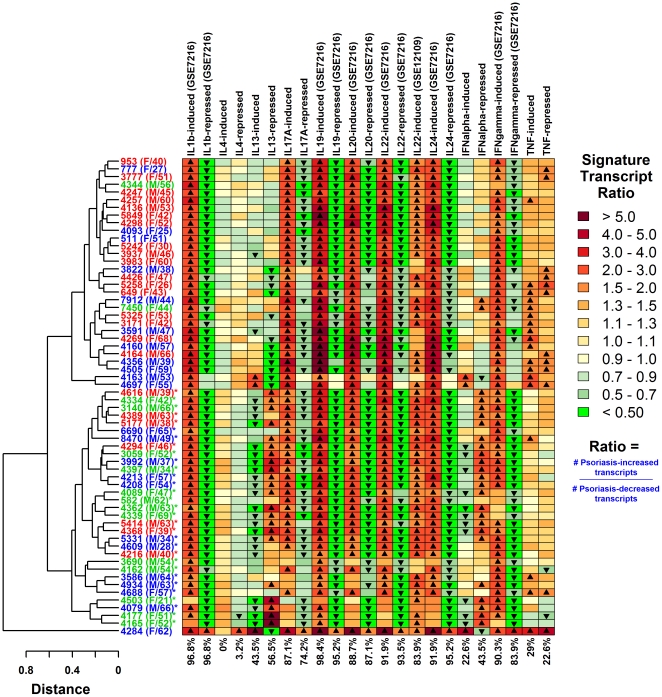
Sub-division of psoriasis lesions into IL-13-strong and IL-13-weak groups based on genome-wide expression profiles. We identified 32 sets of 1000 cytokine-responsive transcripts based upon *in vitro* exposure of keratinocytes to cytokines. For each set (columns) and subject (rows), chart colors denote the ratio of PP-increased to PP-decreased transcripts (see scale and Methods). Up-triangles indicate significant bias towards PP-increased expression (FDR-adjusted P<0.05 and ratio value >1.50). Down-triangles indicate significant bias towards PP-decreased expression (FDR-adjusted P<0.05 and ratio value <0.667). Percentage values (bottom) denote the total fraction of subjects with significant patterns in either direction. Asterisk symbols identify subjects associated with the IL-13-weak group (bottom of dendrogram; 31/62 subjects). All other subjects are associated with IL-13-strong patterns (top of dendrogram; 31/62 subjects). Subject label colors are consistent with those in Figure 1 (strong, moderate and weak inflammatory groups). An expanded version of this figure is provided as supplemental material (Figure S7).

Dermal infiltration by inflammatory cells facilitates development of a cytokine environment that reinforces inflammatory cascades and contributes to keratinocyte hyper-proliferation [16]. This cytokine environment is one factor accounting for *in vivo* transcriptome differences between lesional (PP) and non-lesional (PN) skin samples obtained from a given patient [13], [24].We expected that the *in vivo* abundance and activity of cytokines would be linked to characteristic gene expression responses, which could be used as a transcriptional readout to infer upstream signaling activity associated with individual cytokines (e.g., see algorithm presented by Shimoni et al. [19]). We therefore analyzed PP versus PN differences in terms of cytokine activity signatures, which were calculated using cytokine-responsive transcripts identified from cultured keratinocytes exposed to cytokines (Figures 2 and S7).

A total of 32 signatures were considered, where each signature was based upon PP versus PN expression differences for the 1000 transcripts most strongly induced or repressed by a given cytokine (Figures 2 and S7). For 14 of the 32 signatures, signature scores among patients were significantly correlated with fold-change estimates that reflect the PP vs. PN difference in steady state mRNA level of the associated cytokine (Table S1). However, trends associated with cytokine-encoding mRNA levels did not always correspond with those discerned from cytokine-responsive transcripts. IL-13-induced transcripts, for instance, were largely decreased in PP skin of subjects for which expression of IL-13 mRNA was elevated (*r_s_* = −0.66); conversely, IL-13-repressed transcripts were largely increased in PP skin from those subjects with elevated IL-13 mRNA (*r_s_* = 0.67) (Table S1).

Transcripts induced or repressed by cytokines *in vitro* exhibited clear directional trends with respect to expression level differences between PP and PN samples (Figures 2 and S7). We identified seven cytokines for which gene expression signatures were most consistently associated with PP versus PN expression differences, including IL-1β, IL-17A, IL-19, IL-20, IL-22, IL-24 and IFN-γ. In each of these cases, for more than 85% of subjects, either cytokine-induced transcripts were disproportionately elevated in PP relative to paired PN samples, and/or cytokine-repressed transcripts were disproportionately decreased in PP samples (Figures 2 and S7). The IL-19-induced cytokine signature, for instance, was the most consistent among subjects (Figures 2 and S7). Among the 1000 transcripts induced by IL-19 treatment *in vitro*, 75.8% were, on average, elevated in lesional skin as compared to non-lesional skin, and this percentage was significantly large in all but one patient from the cohort (i.e., 98.4% of patients). These robust trends related to IL-1β, IL-17A, IL-19, IL-20, IL-22, IL-24 and IFN-γ were replicated with respect to an independent dataset with 28 patients (Figure S8) [11].

Only a fraction of cytokine activity signatures followed consistent trends among subjects. We clustered cytokine signatures using hierarchical methods and identified two patient sub-groups (Figures 2, S7 and S9). One sub-group included 31 subjects and exhibited an IL-13-strong pattern, with consistent expression level differences (PP versus PN) among transcripts responsive to keratinocyte treatment with IL-13 (also IFN-α, TNF, IL1-α, IL-17A, and IFN-γ; see Figures 2 and S7, upper branch of dendrogram, no asterisk; Figure S9). The other sub-group included 31 subjects with an IL-13-weak pattern (along with weaker IL1-α, IL-17A, and IFN-γ responses) and attenuation of transcriptional response patterns associated with these cytokines (Figures 2, S7 and S9). Three of the 62 subjects were identified as outliers (i.e., 4284, 4697 and 4163; Grubb's test, P = 1.95×10^−12^) (Figure S10), but each of these tended to follow an IL-13-strong pattern (Figures 2, S7 and S9). Analysis of an independent dataset identified similar trends with respect to IL-13, with 21 subjects approximating the IL-13-weak pattern and 7 approximating the IL-13-strong pattern (Figure S8). However, the IL-13-related patterns were not as consistently linked with corresponding trends for other cytokines (e.g., IFN-α, TNF, IL1-α, IL-17A, and IFN-γ), and the clustering pattern did not identify two patient sub-groups as shown in Figures 2 and S7.

**Figure 3:**
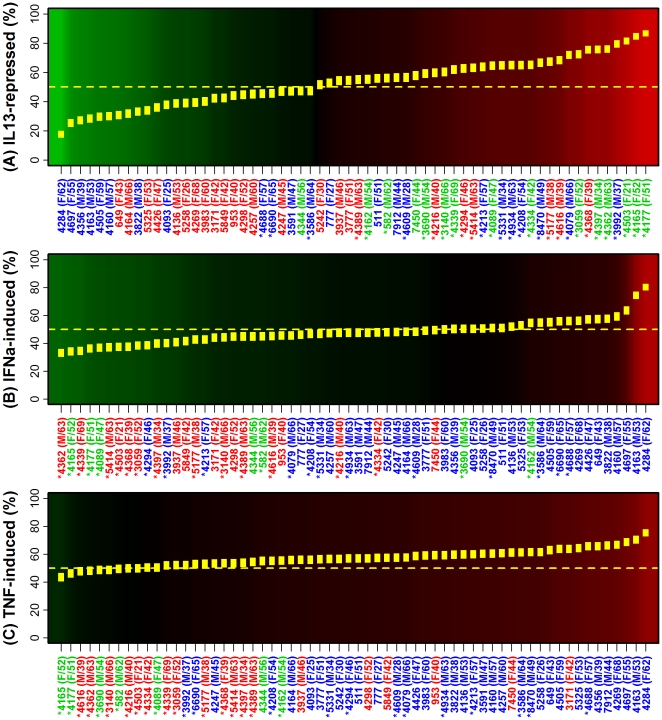
Distribution of IL-13, IFN-α and TNF gene expression signatures among lesional skin samples from 62 patients. We identified sets of 1000 transcripts that were (A) repressed by IL-13 treatment of cultured keratinocytes, (B) induced by IFN-α treatment of keratinocytes and (C) induced by TNF treatment of keratinocytes. For each set and patient, we calculated the percentage of transcripts elevated in lesional (PP) samples as compared to paired non-lesional (PN) samples. In (A)–(C), subjects are ordered according to the estimated proportion of cytokine-responsive transcripts elevated in PP skin relative to PN skin. Yellow boxes outline a 95% confidence interval for this proportion. Label colors are consistent with those in Figure 1 (strong, moderate or weak inflammatory groups). Asterisk symbols identify subjects with IL-13-weak patterns (Figure 2).

**Figure 4:**
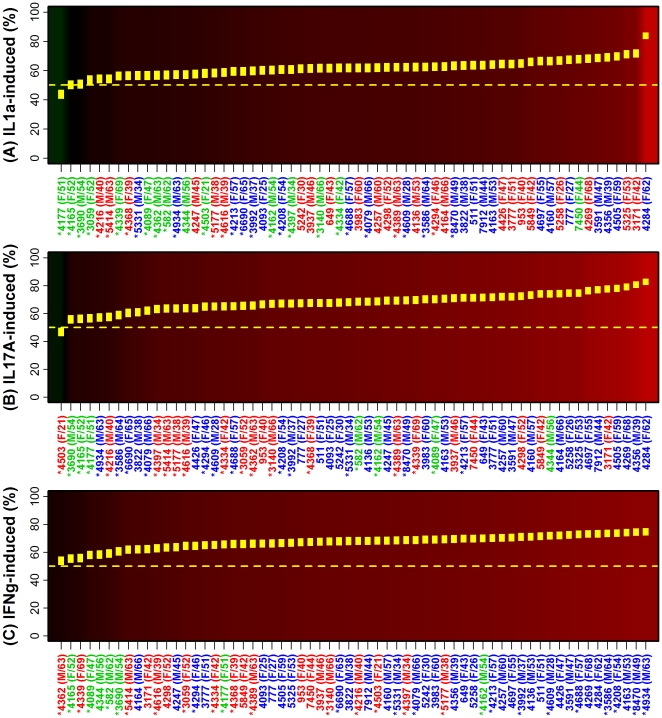
Distribution of IL1-α, IL-17A and IFN-γ gene expression signatures among lesional skin samples from 62 patients. We identified three sets of 1000 transcripts that were (A) induced by IL1-α treatment of cultured keratinocytes, (B) induced by IL-17A treatment of keratinocytes and (C) induced by IFN-γ treatment of keratinocytes. For each set and patient, we calculated the percentage of transcripts elevated in lesional (PP) samples as compared to paired non-lesional (PN) samples. In (A)–(C), subjects are ordered according to the estimated proportion of cytokine-responsive transcripts elevated in PP skin relative to PN skin (as described in the Figure 3 legend).

Local accumulation of key cytokines is both a driver and consequence of inflammatory events, and this connection between cytokine and inflammatory networks was discernible from our analysis (Figures 3 and 4). The behavior of IL-13-repressed transcripts, for instance, was the most variable among the 62 patients (Figure 3A). In one extreme case, only 17.6% (15.2%–20.0%) of IL-13-repressed transcripts were elevated in lesional skin (subject 4284; Figure 3A), and yet at the opposite extreme, 86.8% (84.7%–88.9%) of IL-13-repressed transcripts were elevated in lesional skin from another patient (subject 4177; Figure 3A). This latter type of pattern was closely associated with weak inflammatory signatures (Figure 3A). A comparable trend also emerged among cytokine-induced transcripts (e.g., IFN-α-, TNF-α-, IL1-α-, IL17A- and IFN-γ-induced), with subjects assigned to weak/moderate inflammatory sub-groups exhibiting the weakest elevation of cytokine-induced transcripts in lesional skin samples (Figures 3B, 3C, 4A–4C). To further assess inflammatory-cytokine relationships, we scanned all 1312 two-way combinations between gene expression signatures associated with the 41 cell types and signatures associated with the 32 sets of cytokine-responsive transcripts. This identified 949 cases in which there was significant correlation between an inflammatory and cytokine signature (Spearman rank correlation, *n* = 62 subjects; P<0.05; Figure S11). For example, subjects with elevated expression of TNF-induced transcripts in PP skin also tended to have elevated expression of dendritic cell-associated transcripts in PP skin (*r_s_* = 0.75, P = 1.92×10^−12^; Figure S12A). Likewise, subjects with decreased expression of TNF-repressed transcripts in PP skin tended to have increased expression of monocyte-associated transcripts in PP skin (*r_s_* = −0.57, P = 1.24×10^−6^; Figure S12B).

### Heightened inflammatory and cytokine signatures are only weakly associated with increased psoriasis susceptibility loci risk allele frequency (*REL*, *TRAF3IP2*, *NOS2 and FBXL19*; *n* = 62 patients)

Our results show that psoriasis lesions can be assigned to sub-groups based upon the inflammatory infiltrate or cytokine activity signatures embedded within genome-wide expression profiles (see Table S2 summary). We evaluated whether abundance of psoriasis risk alleles differed between patients assigned to these sub-groups (Figures S13 and S14). Risk alleles for two single nucleotide polymorphism (SNP) markers (rs13017599/*REL* and rs842636/*REL*) were more frequent among the 23 subjects belonging to the strong inflammatory signature group, as compared to the 15 subjects belonging to either the moderate or weak inflammatory group (Cliff's delta = 0.37; P<0.039; FDR-adjusted P = 0.46; Figure S13). However, one SNP marker followed the opposite trend (rs610604/*TNFAIP3*), with elevated risk allele burden among subjects assigned to the weak inflammatory sub-group (delta = −0.382; P = 0.017; FDR-adjusted P = 0.46; Figure S13). A cumulative risk score was calculated by fitting a logistic regression model to risk allele burden scores across 44 psoriasis susceptibility loci (see values in right margin of Figure S13). On average, this cumulative genetic risk score was lower among subjects assigned to the strong inflammatory group, but this difference was of threshold significance (54.5±2.4 versus 62.4±3.4; P = 0.063, Wilcoxon rank-sum test; P = 0.067, two-tailed t-test).

These analyses were repeated with respect to the IL-13-weak (*n* = 31) and IL-13-strong (*n* = 31) sub-groups (Figure S14). We identified three loci for which risk alleles were more frequent in IL-13-strong subjects, including rs13210247/*TRAF3IP2* (Cliff's delta = −0.226; P = 0.012; FDR-adjusted P = 0.363), rs4795067/*NOS2* (Cliff's delta = −0.302, P = 0.025, FDR-adjusted P = 0.363) and rs12924903/FBXL19 (Cliff's delta = −0.289; P = 0.029; FDR-adjusted P = 0.363) (Figure S14). However, with respect to two other psoriasis susceptibility loci (rs1008953/*SDC4* and rs2082412/*IL12B*), risk alleles were enriched in subjects with IL-13-weak patterns (Cliff's delta ≥0.25; P≤0.045; FDR-adjusted P≤0.40) (Figure S14). On average, subjects associated with IL-13-strong and IL-13-weak patterns had similar cumulative genetic risk scores (59.1±2.7 versus 58.7±2.3; P = 0.91; two-tailed t-test).

## Discussion

Treatment protocols for chronic plaque psoriasis do not, at present, draw upon patient-specific genomic data, and there is limited basis for predicting patient responses to systemic therapies. Based upon transcriptome data from 62 patients, we have shown that 24% of lesional skin samples are associated with a weakened inflammatory infiltrate signature, suggestive of decreased infiltration primarily by macrophages and dendritic cells along with monocytes, T-cells and NK cells. Furthermore, half of lesional samples exhibit an “IL-13-weak” signature that is broadly consistent with localized attenuation of the cytokine network. 20% of lesions, moreover, were associated with both groups, with combined diminution of inflammatory and cytokine signatures (e.g., see subjects 4177, 4165, 4089, 3690 and 4162 in Figures 1 and 2). Psoriasis lesions, therefore, may look very similar clinically, with their hallmark features, and likewise, standard immunohistochemistry may not uncover distinctions between lesional skin samples. We have shown, however, that high dimensional transcriptome data can be exploited to effectively uncover a level of “hidden heterogeneity”, which has not been previously described for chronic plaque psoriasis. This provides a new method for stratifying lesional samples according to inflammatory and cytokine activity, which can be developed further to provide a *bona fide* clinical application for patient-specific transcriptome data, with the aim of predicting patient responses to treatment.

Psoriasis plaque formation is very complex and incompletely understood. It involves an interaction between inflammatory and resident tissue cells with both positive and negative feedback cycles, primarily mediated by various growth factors and cytokines [1], [23]. Interactions between T-cells, dendritic cells, macrophages and keratinocytes appear to play a key role, particularly through the production of TNF-α, IFN-γ and IL-17 [1], [22], [24], [25]. This model has guided development of often effective therapies that block interactions between T-cells and antigen presenting cells, along with anti-cytokine treatments, such as anti-TNF and anti-p40 (ustekinumab) [23]. However, short-term (10–12 weeks) response rates to these therapies vary from 50–80% [26], prompting the question of whether a “one-size-fits-all” pathogenic model will be sufficient for understanding the complete spectrum of chronic plaque psoriasis. In our cohort of 62 subjects, a significant TNF cytokine signature was detected in only 18 patients (29%) (Figures 2 and S7). Moreover, among 1000 transcripts induced by TNF in cultured keratinocytes, the proportion of such transcripts elevated in lesional skin varied from 43.4% (subject 4165; 95% CI: 40.3–45%) to 75.3% (subject 4284; 95% CI: 72.6%–78.0%) (Figure 3C). Subjects exhibiting the weakest TNF signatures were associated with the IL-13-weak cytokine group, consistent with a broader attenuation of the cytokine network within this group that is not necessarily limited to the TNF cytokine alone (Figure 3C). This suggests that in many patients, absence of a strong TNF signature may not reflect reduced abundance of TNF *per se*, but rather an overall attenuation of amplifying effects that other cytokines have on the sensitivity of resident cells to TNF signals [24]. More work is needed to determine whether such subjects are particularly non-responsive or apt to develop worsened disease status upon treatment with anti-TNF treatments [3], [4].

We noted strong variability with respect to the behavior of IL-13-responsive transcripts in lesional skin samples (Figure 3A), and overall, IL-13-responsive transcripts best differentiated the two main groups of cytokine signatures in our cohort (Figure 2). IL-13 is a Th2-type cytokine produced by activated T-cells, which can have anti-inflammatory effects in certain cell types [27], but which also stimulates IL-6 production in cultured keratinocytes [28]. The role of IL-13 in the initiation or maintenance of psoriasis lesions is not yet clear. Initial studies showed that IL-13 mRNA levels do not differ significantly between lesional and non-lesional skin samples from psoriasis patients [29], [30]. Subsequent studies confirmed this result, but also showed that mRNA encoding the α1 chain of the IL-13 receptor is over-expressed in lesional skin relative to normal skin from healthy controls [31], and further demonstrated that IL-13 is elevated in synovial fluid of patients with psoriatic arthritis [32]. Moreover, a large genome-wide association study, including 3523 cutaneous-only psoriasis cases, 1755 psoriatic arthritis cases, and 5942 unaffected controls, reported that both cutaneous-only psoriasis and psoriatic arthritis were associated with an exonic IL-13 locus on chromosome 5q31 (rs20541) [33]; however, recent analyses suggest that this association is stronger with respect to psoriatic arthritis, or even specific to psoriatic arthritis with no significant association to cutaneous-only psoriasis [34], [35].

We have identified a range of inflammatory and cytokine activity phenotypes associated with the clinical presentation of chronic plaque psoriasis. Factors that account for this heterogeneity, however, are not completely understood [6], [7]. Potentially, some heterogeneity we identified may reflect distinct developmental stages of psoriasis lesions, with some lesions biopsied during an early initiation stage and others biopsied at a late resolving stage [1], [23]. One report, for instance, has commented that intensity of immunohistochemical IL-13Rα1 staining tends to be stronger in psoriasis lesions that are larger in size [31], which potentially, may reflect differential activation of IL-13-sensitive pathways across stages of plaque development. In addition, the range of molecular phenotypes we observed may be associated with the anatomic region from which lesional biopsies were obtained (e.g., abdomen versus arms), thickness of individual plaques, or the specific region of a plaque that is biopsied (e.g., edge versus center). More extensive studies will thus be necessary to determine whether variability between patients, as documented by our analysis, is in excess of the intra-individual variation attributable to these sources, and more generally to assess how well a single biopsy can represent the individual patient. There is, however, ample evidence to support the genetic basis of psoriasis, along with marked clinical disparities among patients in terms of severity, clinical course and treatment responses [3]–[7]. Despite the need for further data, therefore, we expect that patient-specific factors at least partially contribute to the range of molecular phenotypes observed in psoriasis lesions. These factors may relate to the unique genetic characteristics of individual patients, but may also stem from other (non-genetic) sources of variation influencing systemic or local intensity of inflammatory and cytokine responses, such as body mass index or smoking history [36], [37].

We thus anticipate that methods developed in this study can, in further work, be applied to larger datasets to provide a more fine-scale characterization of heterogeneity within patient cohorts, with the aim of better pinpointing the specific inflammatory and cytokine factors that drive and maintain disease states in specific patient cohorts. Additionally, it will be of interest to determine whether, in larger patient samples, heterogeneity of inflammatory and cytokine gene expression patterns is significantly correlated with response patterns of patients to conservative or biologic therapies, since this would facilitate proactive disease management in clinical settings and better targeting of therapies to the unique characteristics of individual patients. Future work in this direction can provide insights into how patient-specific genetic and environmental factors combine to shape disease mechanisms that underlie this inflammatory skin disorder.

## Materials and Methods

### Ethics statement

All procedures were conducted according to the Declaration of Helsinki principles. Informed written consent was obtained from human subjects under protocols approved by the University of Michigan institutional review board (HUM00037994).

### Patient cohort and sample collection

Sample collection and processing methods followed in this study have been summarized in a previous report [12]. A total of 62 patients were included in the main cohort (30 males and 32 females), where each patient had one or more psoriasis plaques not limited to the scalp area. For cases in which only one plaque was present, a patient was admitted when that plaque occupied more than 1% of the body surface area. 6 mm punch biopsies of lesional (PP) and non-lesional (PN) regions were obtained from each patient under local anesthesia, with all non-lesional biopsies obtained from the buttocks and at least 10 cm away from the nearest active psoriasis plaque. For lesional samples, biopsies were preferentially taken near the central region of active plaques, except in cases where the center was poorly defined due to an irregular boundary. Regions near the edge of individual plaques were avoided to ensure that uninvolved skin was not included within the lesional sample. We utilized non-lesional PN samples from psoriasis patients as controls for identifying transcripts with increased or decreased expression in lesional skin. This approach provides a matched non-lesional sample for each psoriasis patient, which we have used to control for variation with respect to patient-specific factors (e.g., age, sex, gender, etc.). We note, however, that gene expression patterns associated with uninvolved skin from psoriasis patients are expected to differ from those of normal skin from healthy individuals without psoriasis [12].

Among all 62 patients, the average total body surface area covered with psoriasis lesions was 14.2%±1.7% (minimum: 1%; maximum 62%; including palms, head, limbs and trunk). Average surface area did not differ significantly among those with weak, moderate and strong inflammatory patterns (P = 0.96, ANOVA), and likewise, the average surface area did not differ among those with IL-13-weak and IL-13-strong cytokine signatures (P = 0.56, ANOVA). All subjects were between 21 and 69 years of age (average: 49.2±1.5 years), and age did not differ significantly between inflammatory or cytokine groups (P≥0.36, ANOVA). Patients were advised not to use systemic medications for at least 2 weeks prior to sample biopsies, and not to apply topical treatments for at least 1 week prior to biopsies. Our cohort included patients that had previously been treated with the systemic medications Methotrexate (*n* = 8), Enbrel (*n* = 6), Cyclosporine (*n* = 2), Humira (*n* = 2), Soriatane (*n* = 2), Raptiva (*n* = 1), Remicade (*n* = 1), and/or Stelara (*n* = 1). The expected half-life of each medication is less than 2 weeks [38]–[45], and we noted few cases in which prior treatment history was significantly associated with inflammatory or cytokine signature scores (Figure S15).

### Microarray analysis of gene expression

RNA was extracted from lesional (PP) and non-lesional (PN) skin biopsies and processed according to standard Affymetrix protocols [46]. Genome-wide expression in all samples was evaluated using the Human Genome U133 2.0 oligonucleotide array platform (54613 probe sets per array). Annotation for this array platform was obtained from the NetAffx database (Release 31) [47]. The MIAME compliant raw data has been deposited in the Gene Expression Omnibus database and is available under accession GSE13355. We have also analyzed an independent dataset available under accession GSE14905 (Figures S5 and S8) [11], which included PP and PN hybridizations involving the same Affymetrix Human Genome U133 2.0 array platform. The RMA algorithm was used to calculate normalized expression scores for each dataset and for data used to generate inflammation profiles and cytokine activity signatures (see below) [48]. Normalized expression scores for PP and PN samples available from GSE13355 were adjusted for sex and batch effects prior to further analyses. All analyses were conducted using the R statistical software package (with extension packages: ClassDiscovery, fpc, limma, mvoutlier, orddom, outliers, proxy, RColorBrewer, RSQLite, sciplot) [49].

### Calculation of inflammation profiles

Inflammation profiles (Figures 1, S1 and S5) were calculated following the methodology described by Swindell et al. [18]. In brief, we identified “signature transcripts” for each of 354 human cell populations, based upon array samples obtained from the Gene Expression Omnibus database (Human Genome U133 2.0 platform). A selected fraction of these cell populations was used to generate the columns in Figures 1, S1 and S5 (e.g., blood-derived CD4+ T-cells, B-cells, keratinocytes; selection criteria are described below). Because most cell types shown in Figures 1, S1 and S5 represent infiltrating immune cells, we have referred to these patterns as “inflammatory infiltrate signatures” and “inflammation profiles”, consistent with terminology used previously [18]. We note, however, that some populations we considered are in fact resident cell types (e.g., fibroblasts, keratinocytes and epidermis) or sources of immune cells that harbor or give rise to infiltrating cell types but do not directly infiltrate lesional psoriatic skin (e.g., bone marrow).

Signature transcripts for each cell population were identified based upon a two-sample comparison between replicate array hybridizations involving a given cell population (*n* = 4.43 replicates per cell population on average) and a reference set of 21 array samples hybridized with cDNA generated from human skin tissue (see GEO accession ids GSE7307, GSE6281, GSE16161, GSE17539 for description of reference samples). To identify signature transcripts highly expressed within each cell population (relative to human skin), we first used empirical Bayes methods to calculate p-values for all transcripts [50]. Transcripts were then filtered to include only those with higher expression (on average) in the cell population relative to normal skin, and from among this filtered set of transcripts, the top 2000 probe sets with the most significant p-values were isolated. These 2000 transcripts were then ranked according to the fold-change estimate (i.e., average expression in the cell population of interest/average expression in reference skin samples), and the top 1000 of these transcripts (with highest fold-change) were identified and designated as signature transcripts for that cell population. Supplemental data include two example lists of signature transcripts that were generated using this methodology (associated with keratinocytes and blood-derived CD4+ T-cells) (Tables S3 and S4).

Inflammation profiles were next calculated for individual patients based upon the 1000 signature transcripts identified for each of the 354 cell populations. For each patient and cell population, we calculated the fraction of signature transcripts elevated in lesional skin as compared to non-lesional skin (i.e., no. PP-elevated signature transcripts/no. PP-decreased signature transcripts). Significance of this ratio was evaluated according to two criteria [18]. First, we tested whether each ratio was significantly large based upon Fisher's exact test (with p-values adjusted among all 354 cell populations using the conservative Holm method). Secondly, we tested whether a significantly large ratio was obtained (Fisher's exact test) based upon a subset of the original 1000 probe sets, which had been filtered to exclude any signature transcripts of more highly-ranked cell populations (with larger overall PP-elevated/PP-decreased ratios). A ratio was considered significant only if both criteria were met (i.e., black dots in Figures 1, S1 and S5) [18].

Columns in Figures 1, S1 and S5 display results for only a subset of the 354 cell populations we considered (due to space limitation). For instance, with respect to the category “T-cells (Blood, CD3+)” (first column of Figures 1, S1 and S5), a total of six different cell populations were evaluated. In this case, and similarly in other cases, we calculated the average signature transcript ratio across all patients for each of the six populations (*R*_1_, *R*_2_, *R*_3_, *R*_4_, *R*_5_ and *R*_6_) as well as the grand average of these six ratio values (R*). If R* was larger than 1, this indicated an overall trend towards large ratios for this cell population category, and thus as a representative to display we chose the cell population with the largest average signature transcript ratio across patients (i.e., max(*R*_1_, *R*_2_… *R*_6_)). If R* was less than 1, this indicated an overall trend towards smaller ratios for this cell population category, and thus as a representative to display we chose the cell population with the smallest average signature transcript ratio across patients (i.e., min(*R*_1_, *R*_2_, …, *R*_6_)).

### Cytokine activity signatures

Cytokine activity signatures (Figures 2, S7 and S8) were generated based upon 32 sets of cytokine-responsive transcripts identified from *in vitro* experiments in which keratinocytes had been exposed to cytokines. In each experiment, genome-wide expression in exposed and non-exposed keratinocytes was evaluated using the Affymetrix Human Genome U133 2.0 platform. Signatures for TNF, IL-17A, IL-13, IL4, IFN-α and IFN-γ were generated based upon data from our laboratory. These data will be submitted to Gene Expression Omnibus following MIAME standards prior to publication of this manuscript. All additional data was obtained from Gene Expression Omnibus accessions GSE7216, GSE12109 and GSE9120. For each signature, 1000 cytokine-induced or cytokine-repressed transcripts were identified based upon the two-step ranking procedure described above, where the top 2000 transcripts with lowest p-values were first identified (p-values were generated based upon two-sample comparisons and empirical Bayes methods), and then the top 1000 of these transcripts with the highest (or lowest) fold-change ratio were selected and used to generate cytokine-response signatures. With this procedure, ratios (PP-increased/PP-decreased) represented by the color of each square in Figures 2, S7 and S8 are based upon the same number of transcripts (1000).

### Cluster analyses and outlier diagnostics

The inflammatory and cytokine profiles shown in Figures 1, S1, S5, 2, S7 and S8 were clustered using the complete linkage hierarchical approach and the Euclidean distance metric. Distance between profiles was calculated from log_2_-transformed ratios (i.e., no. PP-increased transcripts/no. of PP-decreased transcripts), which had been standardized for each inflammatory cell population or cytokine signature to have a mean of zero and standard deviation of one. In Figures S13 and S14, psoriasis susceptibility loci were clustered according to the number of risk alleles at each loci among the 62 patients. For these analyses, clustering was performed using the complete linkage hierarchical approach and the Manhattan distance metric. To determine whether any subjects should be considered outliers with respect to either inflammatory or cytokine profiles, we first summarized patterns across cell types (or cytokines) by considering the first two principle component axes (Figures S3 and S10). We then tested for the presence of an outlier with respect to each axis individually using Grubb's test [51]. If this test provided evidence for at least one outlier with respect to either axis, subjects were classified as either outliers or non-outliers in the two dimensional space using a multivariate outlier detection algorithm [52]. This approach identified outliers in the bivariate space using the robust Mahalanobis distance between each subject and the bivariate centroid, such that subjects were classified as outliers if this distance exceeded a critical value [52].

## Supporting Information

Figure S1 **Sub-division of psoriasis lesions into strong, moderate and weak inflammatory groups based on genome-wide expression profiles.** This figure is an expanded version of the heatmap shown in Figure 1. Columns from Figure 1 are a subset of those displayed in Figure S1. In both Figures 1 and S1, the clustering pattern among subjects is identical, and has been generated with respect to the complete range of cell types as shown in Figure S1.
(TIF)

Figure S2 **Immunohistochemical detection of T-cell subsets, antigen presenting cells and mononuclear cells in lesional (PP) skin samples from three patients.** Lesional skin samples from three psoriasis patients were stained using CD3 (T-cell), CD4 (helper T-cells/monocytes), CD56 (NK cell/activated T-cells), CD68 (monocytes/macrophages), CD11c (dendritic cells) and elastase (neutrophil) antibodies. The three patients evaluated are also included as separate rows in the dendrograms from Figures 1 and 2 (i.e., subjects 6690, 7450 and 8470). Top row, with H&E, shown in 10× whereas rest of images are shown in 20× magnification.
(TIF)

Figure S3 **No significant outliers with respect to two principle components derived from inflammatory signature scores.** Two principle components were extracted from the full set of inflammatory signature scores calculated for each of the 62 patients (see Figure S1). The first principle component accounted for 51.2% of the total variance, while the second accounted for 18.0% of the total variance. No significant outlier was identified with respect to either the first or second principle component (Grubb's test: P = 0.45 and P = 0.86, respectively). Additionally, no significant bivariate outlier was detected based upon the robust Mahalanobis distance between each point and the bivariate centroid [52].
(TIF)

Figure S4 **Average gene expression profiles of psoriasis lesions assigned to the strong, moderate and weak inflammatory groups.** 62 psoriasis lesions were assigned to either strong (23/62), moderate (24/62) or weak (15/62) inflammatory groups based upon signature transcripts of immune cell populations and their altered expression in lesional (PP) versus non-lesional (PN) skin (see Figures 1 and S1). For each immune cell population and each subject, we calculated the number of signature transcripts with higher expression in PP skin relative to PN skin, divided by the number of signature transcripts with lower expression in PP skin relative to PN skin. The average of this ratio was calculated for each group of patients and is shown in the figure for each cell population (vertical axis). Error bars correspond to the standard error of the ratio value among all subjects assigned to a given group. The average silhouette width was calculated for each cell population with respect to the three groups of subjects (i.e., strong, moderate and weak groups) (top margin). This is a summary measure of intraclass cohesion and class separation, with values ranging from −1 to 1 [53]. The measure approaches zero or will be negative if subjects are not well grouped with respect to a given cell population signature (i.e., there is large variation within groups with little separation between groups). Conversely, positive values suggest that subjects have been placed in an appropriate group with respect to a given cell population signature (i.e., there is little variation within groups and large separation between groups).
(TIF)

Figure S5 **Analysis of an independent dataset supports sub-division of psoriasis lesions into strong, moderate and weak inflammatory patterns.** Inflammation profiles were calculated for 28 patients based on PP and PN samples from a previously published dataset (GSE14905) [11]. Each patient was assigned to one of three sub-groups, including strong (blue labels; 6/28 subjects), moderate (red labels; 16/28 subjects) and weak inflammatory patterns (green labels; 6/28 subjects). Inflammation profile calculations used to generate this figure are consistent with those used to generate Figures 1 and S1, and are further described in the Methods section.
(TIF)

Figure S6 **Distribution of keratinocyte, CD4+ T-cell and dendritic cell gene expression signatures among lesional skin samples from 62 patients with chronic plaque psoriasis.** We identified sets of 1000 signature transcripts highly expressed in (A) keratinocytes, (B) CD4+ T-cells and (C) dendritic cells, respectively. For each set of 1000 transcripts and each of 62 patients, we calculated the percentage of signature transcripts elevated in lesional (PP) samples as compared to paired non-lesional (PN) samples. In (A)–(C), subjects have been ordered according to the estimated percentage of signature transcripts elevated in PP versus PN samples. Subject label colors are consistent with those in Figures 1 and S1, and denote assignment to strong (blue), moderate (red) or weak (green) inflammatory groups. An asterisk symbol is used to denote subjects with IL-13-weak gene expression signatures (see Figures 2 and S7). The yellow box shown for each subject outlines the 95% confidence interval for the estimated proportion of signature transcripts elevated in the PP sample relative to the PN sample.
(TIF)

Figure S7 **Sub-division of psoriasis lesions into IL-13-strong and IL-13-weak groups based on genome-wide expression profiles.** This figure is an expanded version of the heatmap shown in Figure 2. Columns from Figure 2 are a subset of those displayed in Figure S7. In both Figures 2 and S7, the clustering pattern among subjects is identical, and has been generated with respect to the complete range of cytokine signatures as shown in Figure S7.
(TIF)

Figure S8 **Analysis of an independent dataset identifies IL-13-strong and IL-13-weak psoriasis lesions.** Gene expression signatures associated with the *in vitro* responses of keratinocytes to cytokine exposure were analyzed for 28 patients based on lesional (PP) and non-lesional (PN) samples from a previously published dataset (GSE14905) [11]. Cytokine signatures were calculated and patients were clustered using average linkage and the Euclidean distance metric (see Figures 2 and S7). An asterisk symbol is used to denote 21 subjects for which the IL-13 signature approximated the “IL-13-weak” pattern identified in Figures 2 and S7. All other subjects approximated the “IL-13-strong” pattern identified in Figures 2 and S7. Colors within the chart correspond to the number of cytokine-responsive transcripts with higher expression in PP versus PN skin, divided by the number of cytokine-responsive transcripts with lower expression in PP versus PN skin (see legend). For IL-13-weak lesions (asterisk symbol), this ratio is lower among IL-13-induced transcripts, but higher among IL-13-repressed transcripts. For IL-13-strong lesions (no asterisk), this ratio is higher among IL-13-induced transcripts, but lower among IL-13-repressed transcripts.
(TIF)

Figure S9 **Average gene expression profiles of psoriasis lesions assigned to IL-13-weak and IL-13-strong cytokine groups.** 62 psoriasis lesions were assigned to either IL-13-weak (31/62) or IL-13-strong (31/62) groups based upon cytokine-responsive transcripts and their altered expression in lesional (PP) versus non-lesional (PN) skin (see Figures 2 and S7). For each patient, we calculated the number of cytokine-responsive transcripts with higher expression in PP skin relative to PN skin, divided by the number of signature transcripts with lower expression in PP skin relative to PN skin. The average of this ratio was calculated for each group of patients and is shown in the figure (vertical axis). Error bars correspond to the standard error of the ratio value among all subjects within a given group. The average silhouette width was calculated for each cell population with respect to the two groups of subjects (i.e., IL-13-weak and IL-13-strong groups) (top margin). This is a summary measure of intraclass cohesion and class separation, with values ranging from −1 to 1 for each cytokine signature (see Figure S4 legend) [53].
(TIF)

Figure S10 **Three significant outliers with respect to two principle components derived from cytokine signature scores (Subjects 4284, 4697 and 4163).** Two principle components were extracted from full set of cytokine signature scores calculated for each of the 62 patients (see Figure S7). The first principle component accounted for 60.5% of the total variance, while the second accounted for 14.9% of the total variance. At least one significant outlier was present with respect to the first principle component (Grubb's test: P = 1.95×10^−12^), but no outliers were detected with respect to the second principle component (Grubb's test: P = 0.07). Three significant bivariate outliers were detected based upon the robust Mahalanobis distance between each point and the bivariate centroid (i.e., subjects 4284, 4697 and 4163) [52].
(TIF)

Figure S11 **Spearman correlation coefficients between inflammatory and cytokine signature scores (** ***n*** **= 62 patients).** We calculated inflammatory signature scores with respect to 41 cell types (Figures 1 and S1) and cytokine signature scores with respect to 32 sets of cytokine-responsive transcripts (Figures 2 and S7). To assess the relationship between inflammation- and cytokine-associated patterns, we estimated the Spearman rank correlation for all 1312 two-way combinations of inflammatory and cytokine signature scores. For each pairing, red colors denote cases in which subjects with increased expression of cytokine-responsive transcripts (left margin) in PP skin also tend to have increased expression of transcripts highly expressed in a given inflammatory cell type (top margin). Conversely, green colors denote cases in which subjects with increased expression of cytokine-responsive transcripts (left margin) in PP skin also tend to have *decreased* expression of transcripts highly expressed in a given inflammatory cell type (top margin). As an example, Figure S12 shows two examples including one positive and one negative correlation (i.e., TNF-induced signature versus infected dendritic cell signature, *r_s_* = 0.75; TNF-repressed signature versus infected monocyte signature, *r_s_* = −0.57). Cytokine and inflammatory signature scores in each comparison were, if necessary, adjusted such that each score was based upon a non-overlapping set of transcripts (i.e., any shared transcripts were filtered out prior to calculation of scores for each subject and estimation of the correlation coefficient). Rows and columns of the heatmap have been clustered using complete linkage and the Euclidean distance metric. For each set of cytokine-responsive transcripts (i.e., each row), the largest correlation is outlined in blue while the most negative correlation is outlined in magenta (see legend).
(TIF)

Figure S12 **Shifts in the expression of TNF-responsive transcripts in PP skin (versus PN skin) co-occur with shifts in the expression of transcripts that are highly expressed in dendritic cells and monocytes (** ***n*** **= 62 subjects).** We screened 1312 two-way combinations involving inflammatory and cytokine signatures to determine which were significantly associated among the 62 subjects included in our cohort (Figure S11). With respect to TNF-induced transcripts, the strongest positive association was identified with respect to the signature calculated from transcripts with high expression in infected dendritic cells (*r_s_* = 0.75, part A). With respect to TNF-repressed transcripts, the strongest negative association was identified with respect to the signature calculated from transcripts with high expression in infected monocytes (*r_s_* = −0.57, part B). In both (A) and (B), all 62 subjects included in the cohort are plotted with respect to each cytokine and inflammatory signature. The vertical axis indicates the proportion of TNF-responsive transcripts elevated in PP (versus PN) skin from a given subject. The horizontal axis denotes patterns associated with transcripts that have high expression in (A) infected dendritic cells or (B) infected monocytes, and corresponds to the proportion of such transcripts elevated in PP (versus PN) skin from each subject. In both (A) and (B), transcripts used to calculate the TNF signature for each subject are distinct from those used to calculate the dendritic cell (part A) or monocyte (part B) signature (i.e., any shared transcripts were removed).
(TIF)

Figure S13 **Frequency of risk alleles at 44 psoriasis susceptibility loci in 62 patients clustered according to inflammatory gene expression signatures.** The 62 patients included in our cohort were clustered according to inflammatory gene expression signatures (see Figures 1 and S1; blue, red and green labels correspond to strong, moderate and weak sub-groups, respectively). Colors in the chart indicate whether subjects are homozygous for psoriasis risk alleles (red), heterozygous (orange) or non-carriers (grey), where each column corresponds to a different psoriasis susceptibility locus. Blank (white) regions represent cases where data is not available. Susceptibility loci (columns) have been clustered according to similarity across the 62 subjects (based on the Manhattan distance metric). For each locus, we evaluated whether risk alleles were more frequent in subjects associated with strong inflammatory patterns (blue labels) relative to those associated with weak inflammatory patterns (green labels). Estimates of Cliff's delta (Δ) are listed along the bottom margin of the chart (−1≤Δ≤1). Positive estimates (navy blue font) indicate a higher risk allele frequency in subjects with strong inflammatory patterns, and negative estimates (dark pink font) indicate lower risk allele frequency in subjects with strong inflammatory patterns. Significant estimates of Δ (P<0.05; prior to FDR adjustment) are denoted by three asterisk symbols (***). The right margin of the chart lists cumulative genetic risk scores calculated for each subject. Cumulative genetic risk scores were calculated by first fitting a logistic regression model, with risk allele burden (0, 1 or 2) at the 44 loci as predictors. The model was fit based upon marker data from an external training set of 2568 psoriasis cases and 2525 control subjects. This fitted logistic regression model was then applied to genotype data from our 62 subjects to calculate the genetic-based probability that a subject is a psoriasis case (though all 62 subjects are verified psoriasis cases). Risk scores in the right margin can thus be interpreted as probabilities, with larger values (>50) indicative of a genetic profile more consistent with psoriasis cases (i.e., with respect to the 44 risk loci considered). For calculation of cumulative genetic risk scores, missing data were imputed using the modal number of risk alleles across all subjects for a given locus. Risk scores were not calculated for subjects untyped at more than 15 loci.
(TIF)

Figure S14 **Frequency of risk alleles at 44 psoriasis susceptibility loci in 62 patients clustered according to cytokine-specific gene expression signatures.** The 62 patients included in our cohort were clustered according to gene expression signatures representing the transcriptional response patterns of cultured keratinocytes treated with cytokines (see Figures 2 and S7; IL-13-weak subjects are denoted by an asterisk symbol). The interpretation of heatmap colors and calculated numerical values is consistent with Figure S13. However, in this figure, estimates of Cliff's delta (Δ) (−1≤Δ≤1) (bottom margin) were generated by testing whether risk allele frequency is elevated in IL-13-weak subjects (asterisk symbols). Positive values of Δ (navy blue) denote elevated risk allele frequency in subjects assigned to the IL-13-weak group, while negative values denote decreased risk allele frequency in subjects assigned to the IL-13-weak group. Significant estimates of Δ (P<0.05; prior to FDR adjustment) are indicated by three asterisk symbols (***). Cumulative genetic risk scores calculated for each subject are listed in the right margin (see Figure S13 legend for details).
(TIF)

Figure S15 **Psoriasis treatment history and its relationship with inflammatory and cytokine signatures.** The 62 patients in our cohort were advised not to use systemic medications for at least 2 weeks prior to sample biopsies, and not to apply topical treatments for at least 1 week prior to biopsies. Patients completed a questionnaire in which they listed all therapies previously used to treat their condition. Ten prior treatments were reported among the 62 patients (left margin in A and B). For each treatment, a two-sample t-test was performed with respect to each inflammatory (part A) or cytokine (part B) signature in order to determine whether signature scores differed significantly among the *n* subjects reporting a given treatment history (compared with all other subjects that did not report the same treatment history). Colors correspond to the value of the T statistic generated from each two-sample t-test. Red colors denote a trend towards elevated signature scores among the *n* subjects reporting the treatment history listed in each row (see legend). Green colors denote a trend towards decreased signature scores among the *n* subjects reporting the treatment history listed in each row (see legend). Filled triangles denote significant T statistics based upon FDR-corrected p-values, while open triangles indicate significant T statistics based upon raw p-values prior to multiple test adjustment.
(TIF)

Table S1 **Steady state mRNA levels of cytokines and their association with cytokine signature scores calculated from sets of cytokine-responsive transcripts.** We identified 32 sets of 1000 cytokine-responsive transcripts in cultured keratinocytes (i.e., cytokine-induced or cytokine-repressed transcripts). For each set and each of 62 patients, we calculated a cytokine signature score, equal to the number of transcripts (of 1000) with higher expression in PP skin relative to PN skin, divided by the number of transcripts (of 1000) with lower expression in PP skin relative to PN skin. Additionally, for each set and each patient, we calculated the fold-change in steady state mRNA level between PP and PN skin. This table lists correlations between these fold-change estimates and cytokine signature scores across the 62 patients.
(PDF)

Table S2 **Classification of 62 psoriasis lesions based upon genome-wide expression patterns.** Psoriasis lesions from 62 patients were assigned to strong, moderate or weak inflammatory groups (Figures 1, S1 and S4), as well as to IL-13-strong or IL-13-weak groups (Figures 2, S7 and S9). This table lists the number of patients assigned to each of the inflammatory-cytokine group combinations.
(PDF)

Table S3 **Set of 1000 signature transcripts with high expression in keratinocytes relative to normal skin.** Heatmaps shown in Figures 1, S1 and S5 are based upon the expression patterns of cell type-specific “signature transcripts” in lesional (PP) and non-lesional (PN) skin samples. This table provides an example of the 1000 signature transcripts associated with one cell type (keratinocytes).
(PDF)

Table S4 **Set of 1000 signature transcripts with high expression in (blood-derived) CD4+ T-cells relative to normal skin.** Heatmaps shown in Figures 1, S1 and S5 are based upon the expression patterns of cell type-specific “signature transcripts” in lesional (PP) and non-lesional (PN) skin samples. This table provides an example of the 1000 signature transcripts associated with one cell type (blood-derived CD4+ T-cells).
(PDF)
