# Supplementary figures and images for: Heterogeneity of Inflammatory and Cytokine Networks in Chronic Plaque Psoriasis
Source: PLoS One. 2012 Mar 29;7(3):e34594. doi: 10.1371/journal.pone.0034594 (PMC3315545; doi:10.1371/journal.pone.0034594)

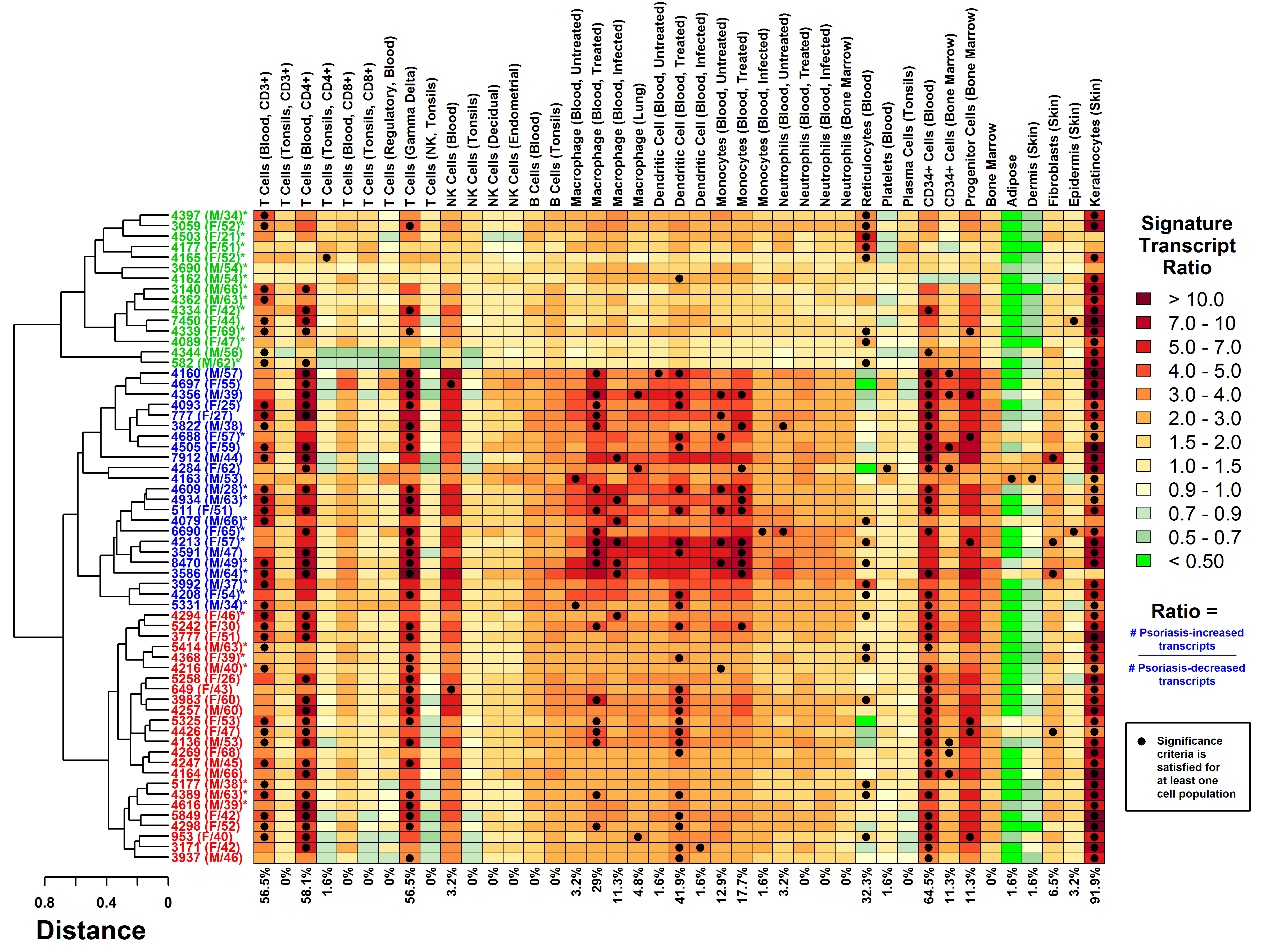

Supplement: Figure S1 — Sub-division of psoriasis lesions into strong, moderate and weak inflammatory groups based on genome-wide expression profiles. This figure is an expanded version of the heatmap shown in Figure 1. Columns from Figure 1 are a subset of those displayed in Figure S1. In both Figures 1 and S1, the clustering pattern among subjects is identical, and has been generated with respect to the complete range of cell types as shown in Figure S1. (TIF) [file pone.0034594.s001.tif]

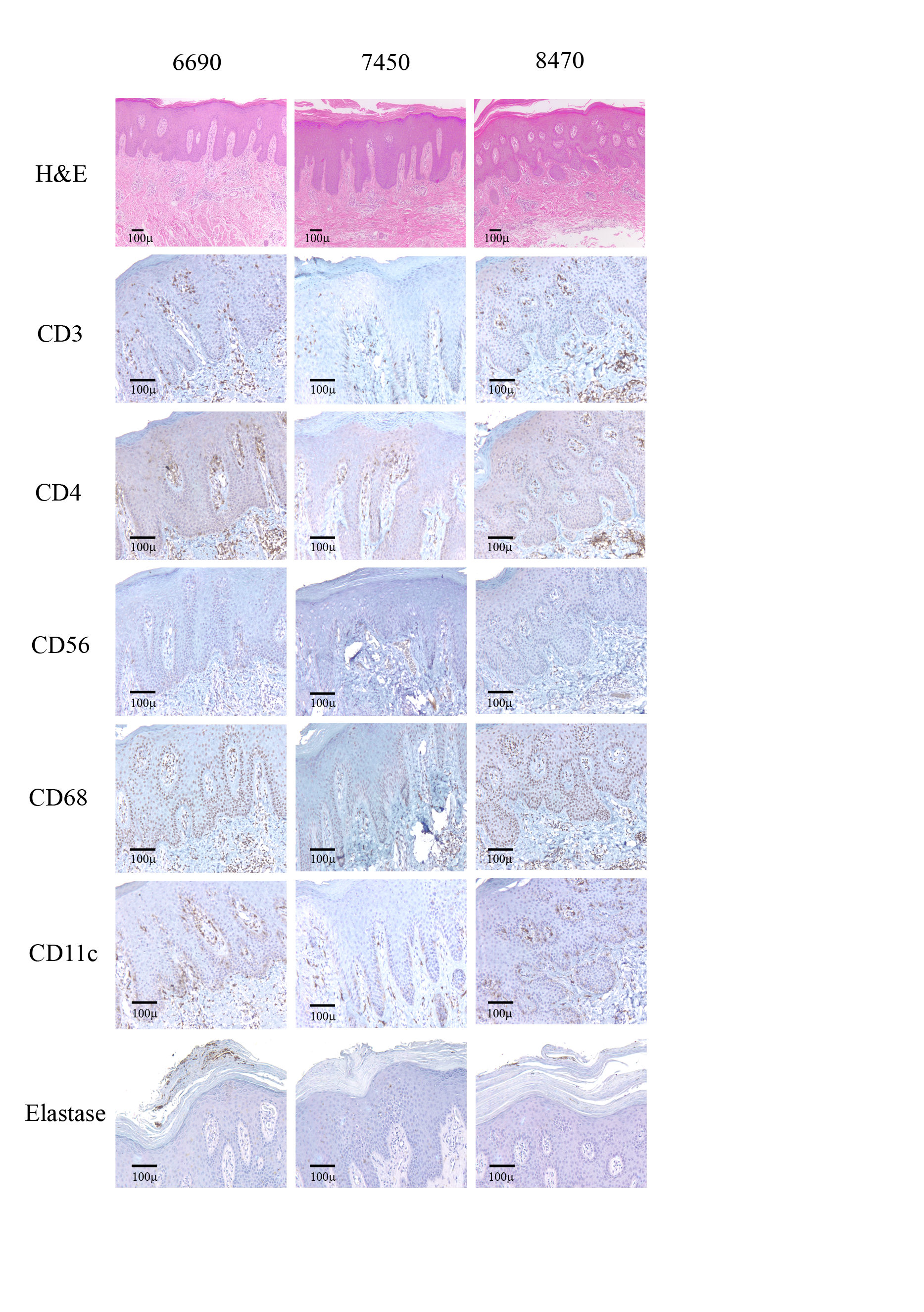

Supplement: Figure S2 — Immunohistochemical detection of T-cell subsets, antigen presenting cells and mononuclear cells in lesional (PP) skin samples from three patients. Lesional skin samples from three psoriasis patients were stained using CD3 (T-cell), CD4 (helper T-cells/monocytes), CD56 (NK cell/activated T-cells), CD68 (monocytes/macrophages), CD11c (dendritic cells) and elastase (neutrophil) antibodies. The three patients evaluated are also included as separate rows in the dendrograms from Figures 1 and 2 (i.e., subjects 6690, 7450 and 8470). Top row, with H&E, shown in 10× whereas rest of images are shown in 20× magnification. (TIF) [file pone.0034594.s002.tif]

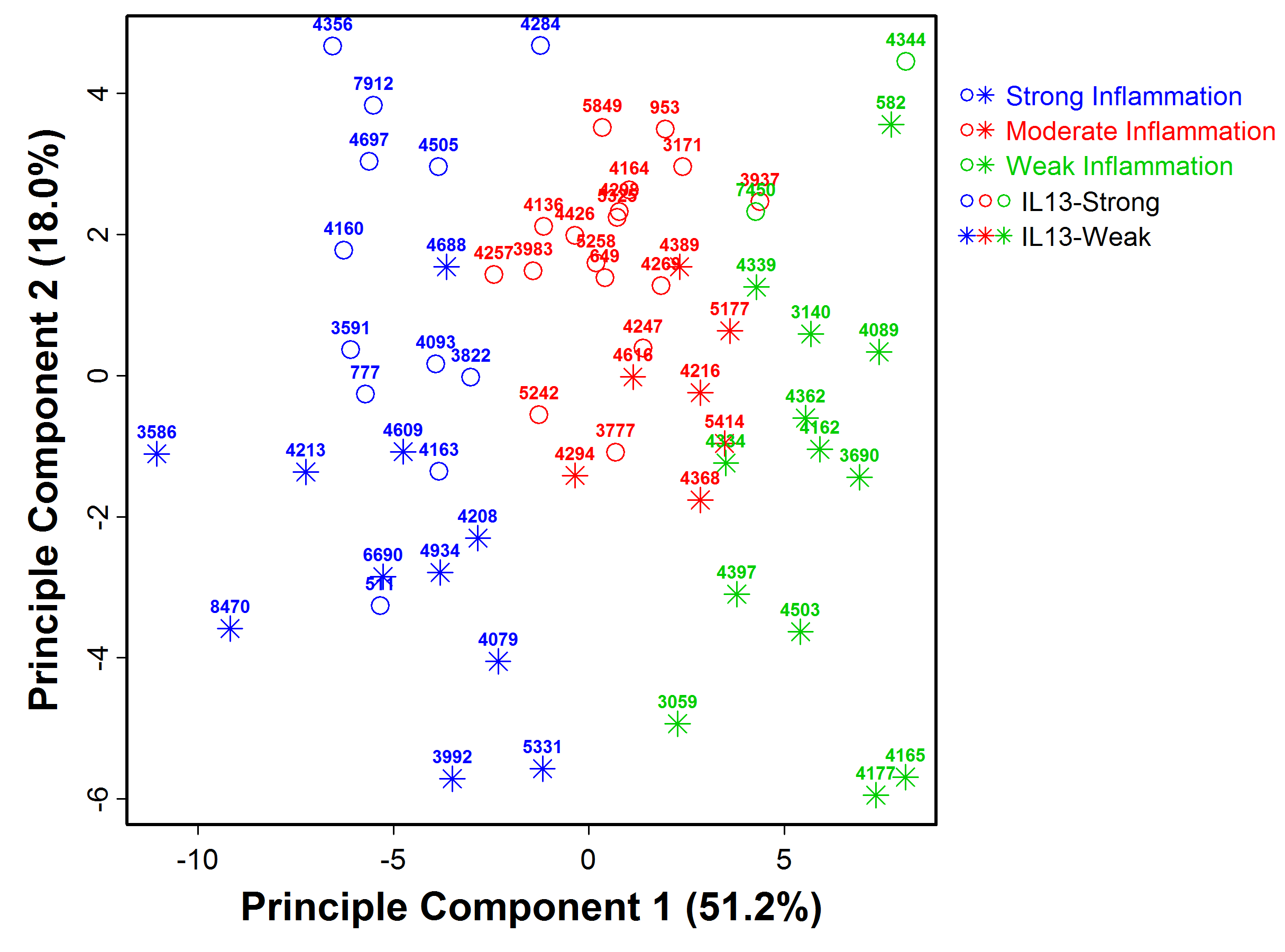

Supplement: Figure S3 — No significant outliers with respect to two principle components derived from inflammatory signature scores. Two principle components were extracted from the full set of inflammatory signature scores calculated for each of the 62 patients (see Figure S1). The first principle component accounted for 51.2% of the total variance, while the second accounted for 18.0% of the total variance. No significant outlier was identified with respect to either the first or second principle component (Grubb's test: P = 0.45 and P = 0.86, respectively). Additionally, no significant bivariate outlier was detected based upon the robust Mahalanobis distance between each point and the bivariate centroid [52]. (TIF) [file pone.0034594.s003.tif]

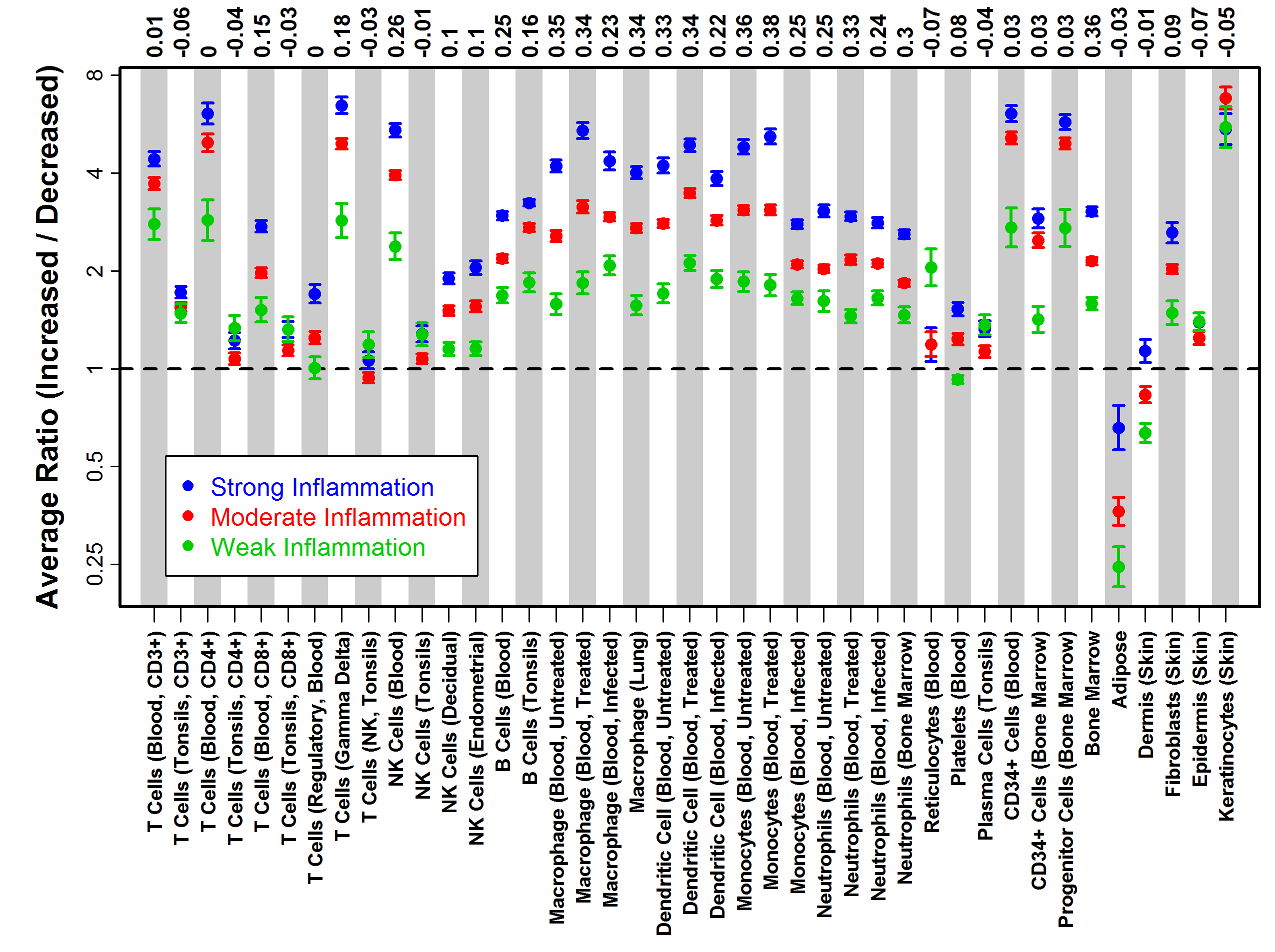

Supplement: Figure S4 — Average gene expression profiles of psoriasis lesions assigned to the strong, moderate and weak inflammatory groups. 62 psoriasis lesions were assigned to either strong (23/62), moderate (24/62) or weak (15/62) inflammatory groups based upon signature transcripts of immune cell populations and their altered expression in lesional (PP) versus non-lesional (PN) skin (see Figures 1 and S1). For each immune cell population and each subject, we calculated the number of signature transcripts with higher expression in PP skin relative to PN skin, divided by the number of signature transcripts with lower expression in PP skin relative to PN skin. The average of this ratio was calculated for each group of patients and is shown in the figure for each cell population (vertical axis). Error bars correspond to the standard error of the ratio value among all subjects assigned to a given group. The average silhouette width was calculated for each cell population with respect to the three groups of subjects (i.e., strong, moderate and weak groups) (top margin). This is a summary measure of intraclass cohesion and class separation, with values ranging from −1 to 1 [53]. The measure approaches zero or will be negative if subjects are not well grouped with respect to a given cell population signature (i.e., there is large variation within groups with little separation between groups). Conversely, positive values suggest that subjects have been placed in an appropriate group with respect to a given cell population signature (i.e., there is little variation within groups and large separation between groups). (TIF) [file pone.0034594.s004.tif]

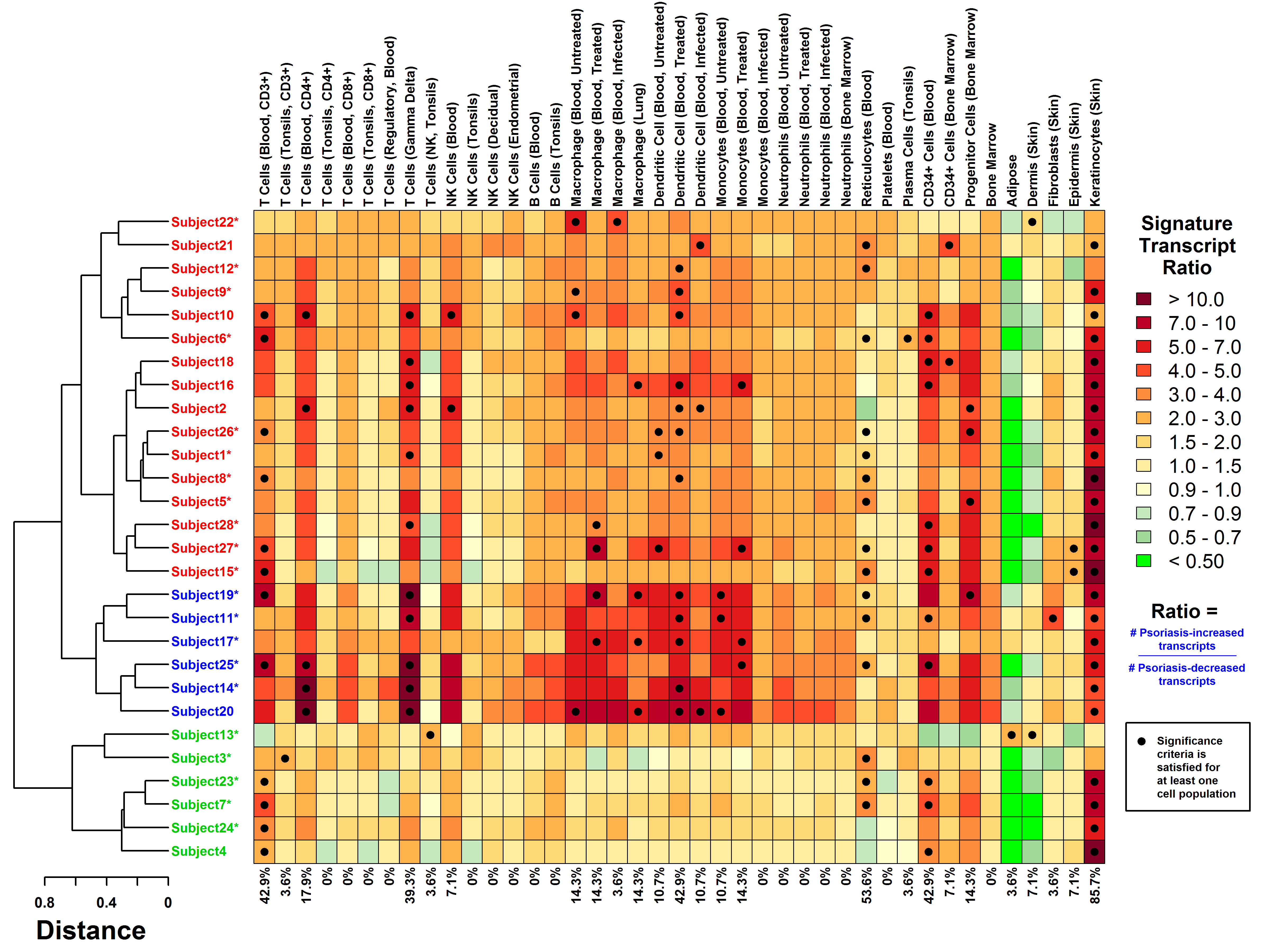

Supplement: Figure S5 — Analysis of an independent dataset supports sub-division of psoriasis lesions into strong, moderate and weak inflammatory patterns. Inflammation profiles were calculated for 28 patients based on PP and PN samples from a previously published dataset (GSE14905) [11]. Each patient was assigned to one of three sub-groups, including strong (blue labels; 6/28 subjects), moderate (red labels; 16/28 subjects) and weak inflammatory patterns (green labels; 6/28 subjects). Inflammation profile calculations used to generate this figure are consistent with those used to generate Figures 1 and S1, and are further described in the Methods section. (TIF) [file pone.0034594.s005.tif]

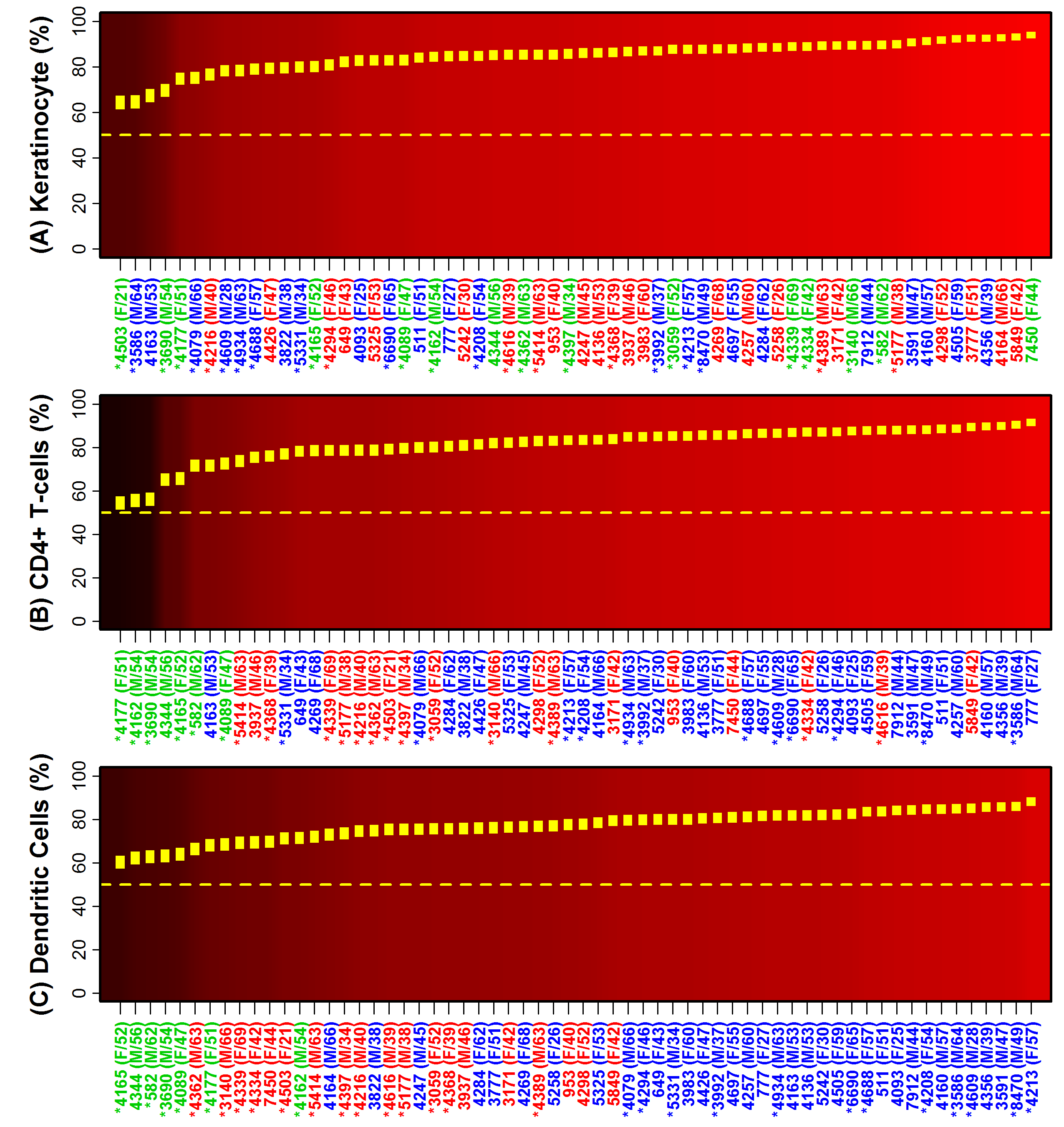

Supplement: Figure S6 — Distribution of keratinocyte, CD4+ T-cell and dendritic cell gene expression signatures among lesional skin samples from 62 patients with chronic plaque psoriasis. We identified sets of 1000 signature transcripts highly expressed in (A) keratinocytes, (B) CD4+ T-cells and (C) dendritic cells, respectively. For each set of 1000 transcripts and each of 62 patients, we calculated the percentage of signature transcripts elevated in lesional (PP) samples as compared to paired non-lesional (PN) samples. In (A)–(C), subjects have been ordered according to the estimated percentage of signature transcripts elevated in PP versus PN samples. Subject label colors are consistent with those in Figures 1 and S1, and denote assignment to strong (blue), moderate (red) or weak (green) inflammatory groups. An asterisk symbol is used to denote subjects with IL-13-weak gene expression signatures (see Figures 2 and S7). The yellow box shown for each subject outlines the 95% confidence interval for the estimated proportion of signature transcripts elevated in the PP sample relative to the PN sample. (TIF) [file pone.0034594.s006.tif]

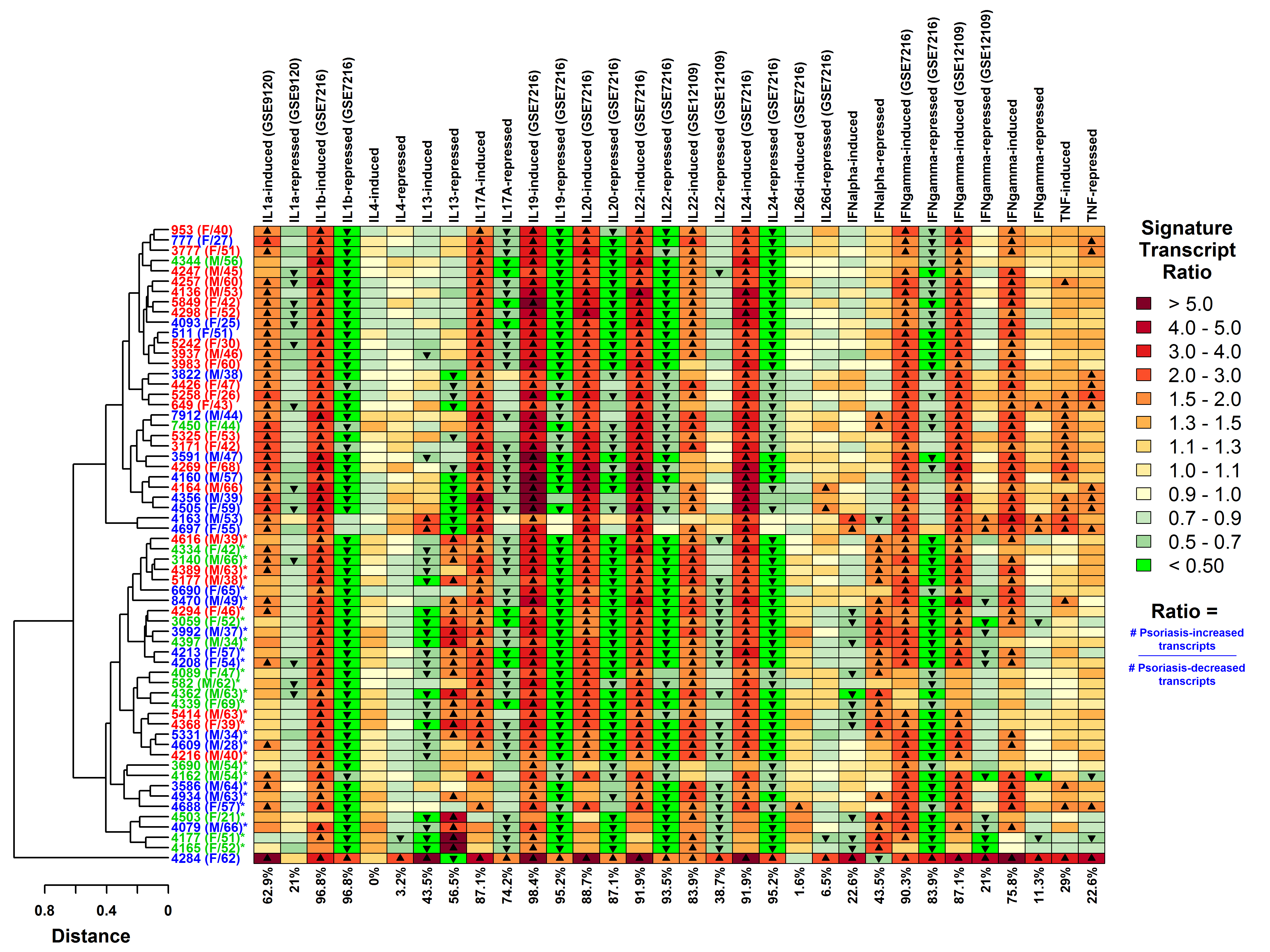

Supplement: Figure S7 — Sub-division of psoriasis lesions into IL-13-strong and IL-13-weak groups based on genome-wide expression profiles. This figure is an expanded version of the heatmap shown in Figure 2. Columns from Figure 2 are a subset of those displayed in Figure S7. In both Figures 2 and S7, the clustering pattern among subjects is identical, and has been generated with respect to the complete range of cytokine signatures as shown in Figure S7. (TIF) [file pone.0034594.s007.tif]

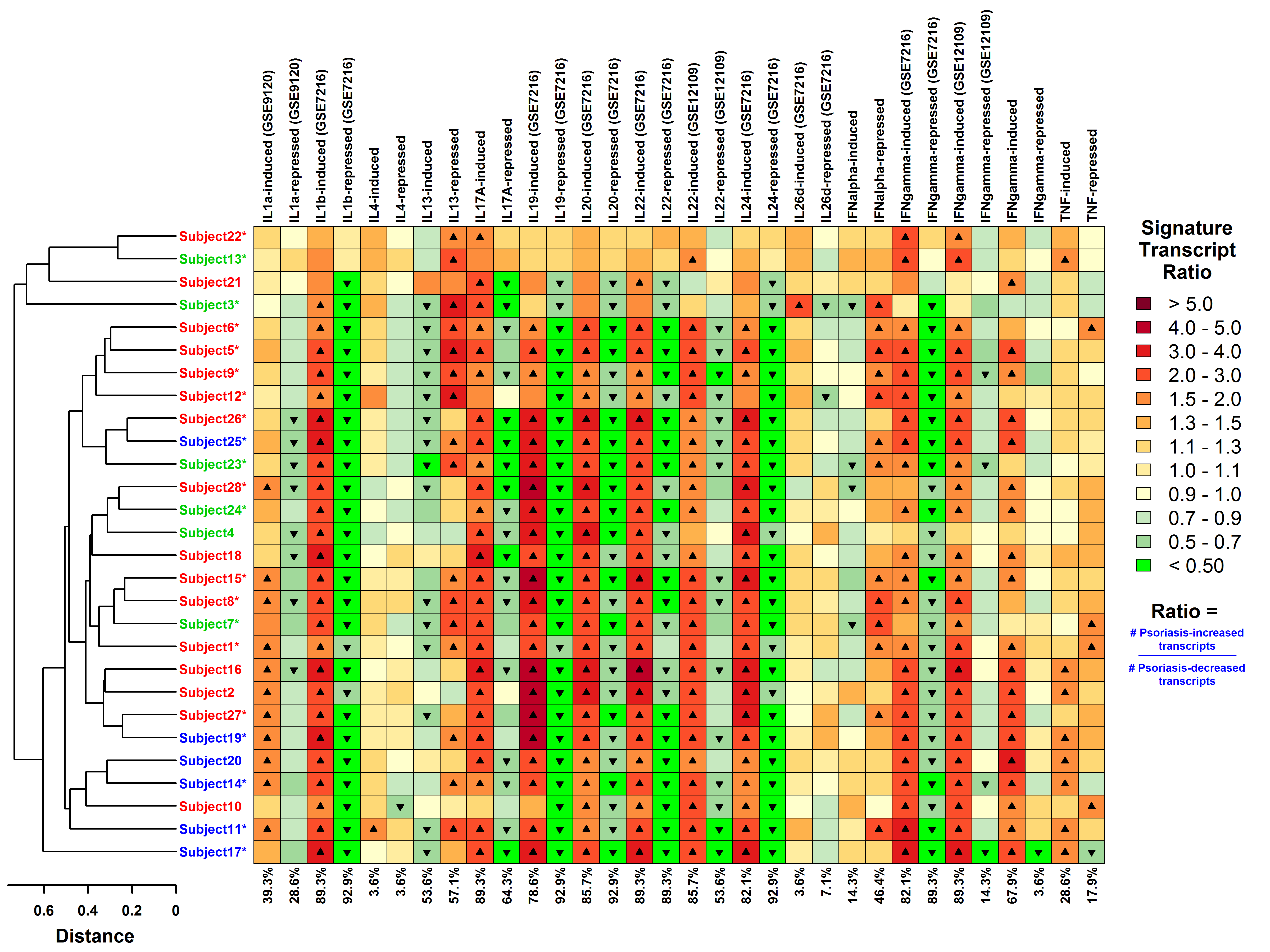

Supplement: Figure S8 — Analysis of an independent dataset identifies IL-13-strong and IL-13-weak psoriasis lesions. Gene expression signatures associated with the in vitro responses of keratinocytes to cytokine exposure were analyzed for 28 patients based on lesional (PP) and non-lesional (PN) samples from a previously published dataset (GSE14905) [11]. Cytokine signatures were calculated and patients were clustered using average linkage and the Euclidean distance metric (see Figures 2 and S7). An asterisk symbol is used to denote 21 subjects for which the IL-13 signature approximated the “IL-13-weak” pattern identified in Figures 2 and S7. All other subjects approximated the “IL-13-strong” pattern identified in Figures 2 and S7. Colors within the chart correspond to the number of cytokine-responsive transcripts with higher expression in PP versus PN skin, divided by the number of cytokine-responsive transcripts with lower expression in PP versus PN skin (see legend). For IL-13-weak lesions (asterisk symbol), this ratio is lower among IL-13-induced transcripts, but higher among IL-13-repressed transcripts. For IL-13-strong lesions (no asterisk), this ratio is higher among IL-13-induced transcripts, but lower among IL-13-repressed transcripts. (TIF) [file pone.0034594.s008.tif]

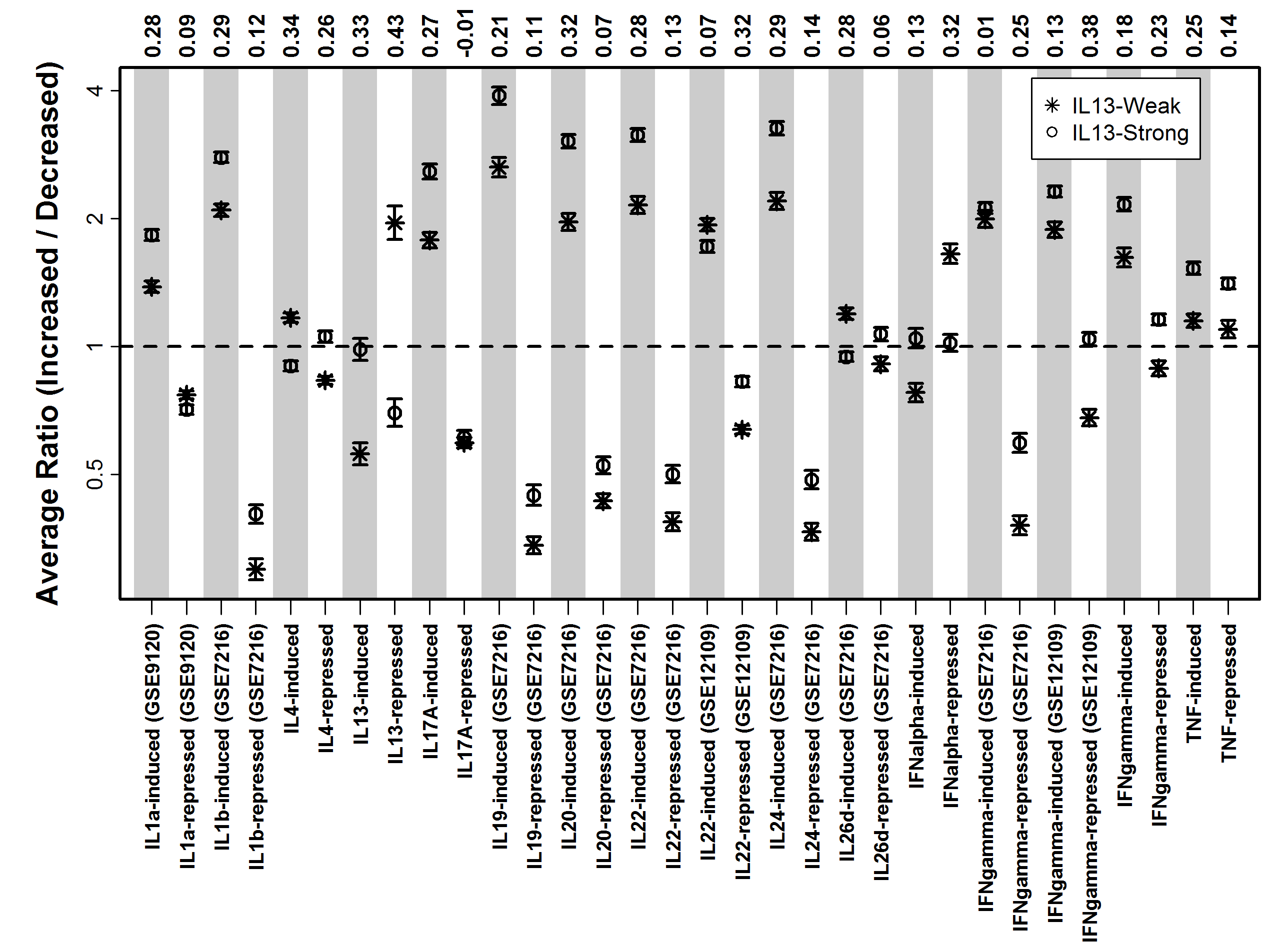

Supplement: Figure S9 — Average gene expression profiles of psoriasis lesions assigned to IL-13-weak and IL-13-strong cytokine groups. 62 psoriasis lesions were assigned to either IL-13-weak (31/62) or IL-13-strong (31/62) groups based upon cytokine-responsive transcripts and their altered expression in lesional (PP) versus non-lesional (PN) skin (see Figures 2 and S7). For each patient, we calculated the number of cytokine-responsive transcripts with higher expression in PP skin relative to PN skin, divided by the number of signature transcripts with lower expression in PP skin relative to PN skin. The average of this ratio was calculated for each group of patients and is shown in the figure (vertical axis). Error bars correspond to the standard error of the ratio value among all subjects within a given group. The average silhouette width was calculated for each cell population with respect to the two groups of subjects (i.e., IL-13-weak and IL-13-strong groups) (top margin). This is a summary measure of intraclass cohesion and class separation, with values ranging from −1 to 1 for each cytokine signature (see Figure S4 legend) [53]. (TIF) [file pone.0034594.s009.tif]

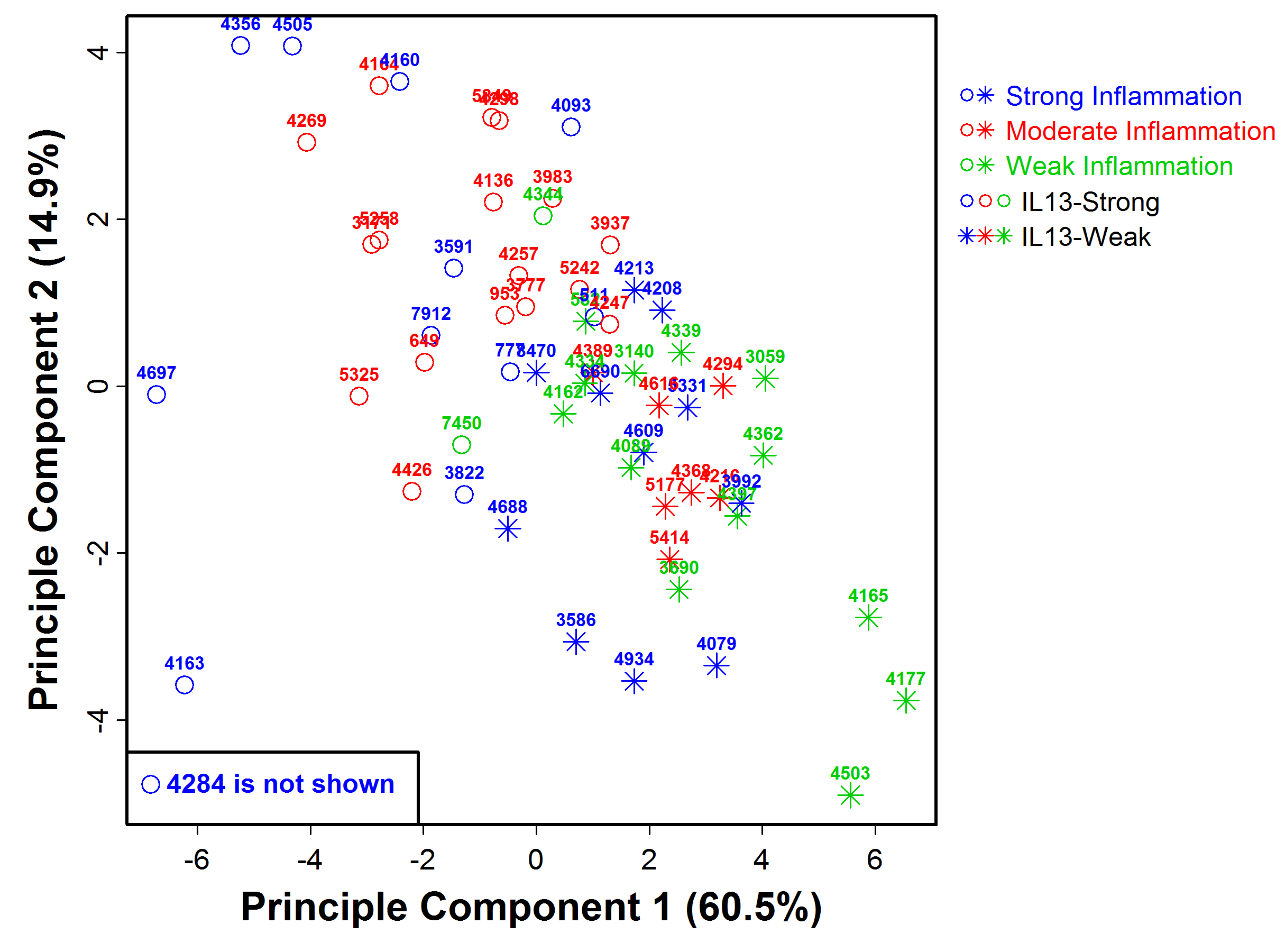

Supplement: Figure S10 — Three significant outliers with respect to two principle components derived from cytokine signature scores (Subjects 4284, 4697 and 4163). Two principle components were extracted from full set of cytokine signature scores calculated for each of the 62 patients (see Figure S7). The first principle component accounted for 60.5% of the total variance, while the second accounted for 14.9% of the total variance. At least one significant outlier was present with respect to the first principle component (Grubb's test: P = 1.95×10^−12^), but no outliers were detected with respect to the second principle component (Grubb's test: P = 0.07). Three significant bivariate outliers were detected based upon the robust Mahalanobis distance between each point and the bivariate centroid (i.e., subjects 4284, 4697 and 4163) [52]. (TIF) [file pone.0034594.s010.tif]

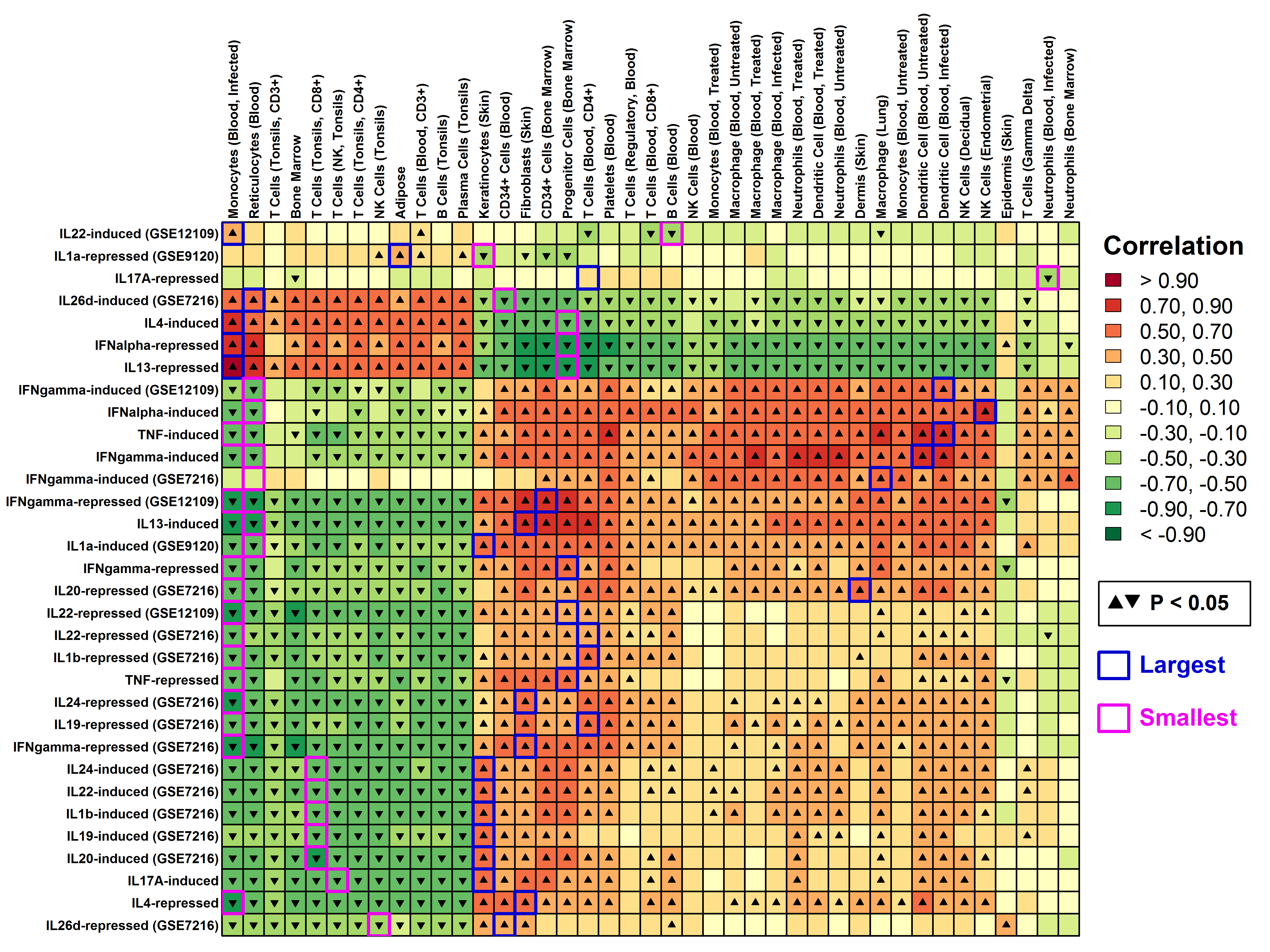

Supplement: Figure S11 — Spearman correlation coefficients between inflammatory and cytokine signature scores ( n = 62 patients). We calculated inflammatory signature scores with respect to 41 cell types (Figures 1 and S1) and cytokine signature scores with respect to 32 sets of cytokine-responsive transcripts (Figures 2 and S7). To assess the relationship between inflammation- and cytokine-associated patterns, we estimated the Spearman rank correlation for all 1312 two-way combinations of inflammatory and cytokine signature scores. For each pairing, red colors denote cases in which subjects with increased expression of cytokine-responsive transcripts (left margin) in PP skin also tend to have increased expression of transcripts highly expressed in a given inflammatory cell type (top margin). Conversely, green colors denote cases in which subjects with increased expression of cytokine-responsive transcripts (left margin) in PP skin also tend to have decreased expression of transcripts highly expressed in a given inflammatory cell type (top margin). As an example, Figure S12 shows two examples including one positive and one negative correlation (i.e., TNF-induced signature versus infected dendritic cell signature, rs = 0.75; TNF-repressed signature versus infected monocyte signature, rs = −0.57). Cytokine and inflammatory signature scores in each comparison were, if necessary, adjusted such that each score was based upon a non-overlapping set of transcripts (i.e., any shared transcripts were filtered out prior to calculation of scores for each subject and estimation of the correlation coefficient). Rows and columns of the heatmap have been clustered using complete linkage and the Euclidean distance metric. For each set of cytokine-responsive transcripts (i.e., each row), the largest correlation is outlined in blue while the most negative correlation is outlined in magenta (see legend). (TIF) [file pone.0034594.s011.tif]

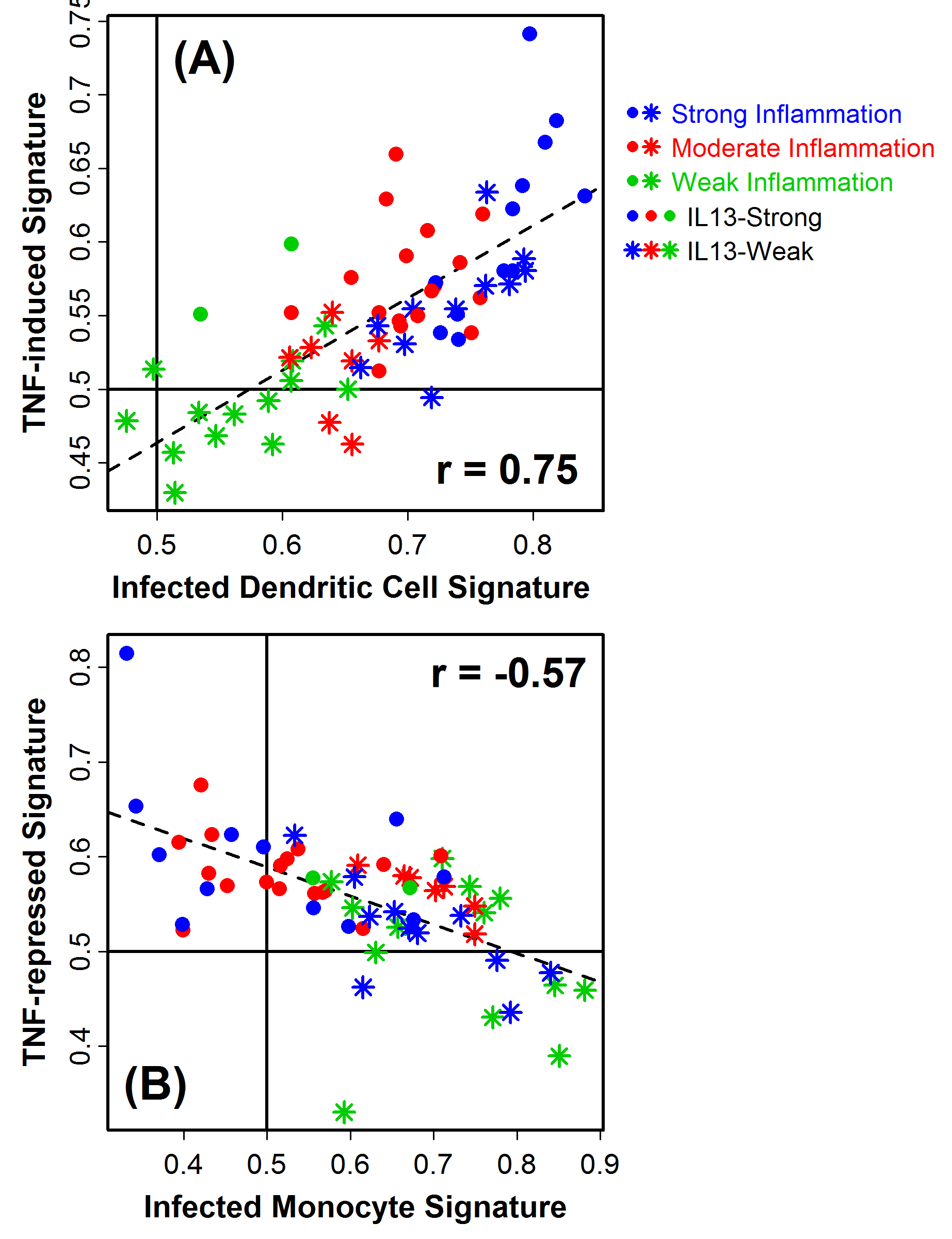

Supplement: Figure S12 — Shifts in the expression of TNF-responsive transcripts in PP skin (versus PN skin) co-occur with shifts in the expression of transcripts that are highly expressed in dendritic cells and monocytes ( n = 62 subjects). We screened 1312 two-way combinations involving inflammatory and cytokine signatures to determine which were significantly associated among the 62 subjects included in our cohort (Figure S11). With respect to TNF-induced transcripts, the strongest positive association was identified with respect to the signature calculated from transcripts with high expression in infected dendritic cells (rs = 0.75, part A). With respect to TNF-repressed transcripts, the strongest negative association was identified with respect to the signature calculated from transcripts with high expression in infected monocytes (rs = −0.57, part B). In both (A) and (B), all 62 subjects included in the cohort are plotted with respect to each cytokine and inflammatory signature. The vertical axis indicates the proportion of TNF-responsive transcripts elevated in PP (versus PN) skin from a given subject. The horizontal axis denotes patterns associated with transcripts that have high expression in (A) infected dendritic cells or (B) infected monocytes, and corresponds to the proportion of such transcripts elevated in PP (versus PN) skin from each subject. In both (A) and (B), transcripts used to calculate the TNF signature for each subject are distinct from those used to calculate the dendritic cell (part A) or monocyte (part B) signature (i.e., any shared transcripts were removed). (TIF) [file pone.0034594.s012.tif]

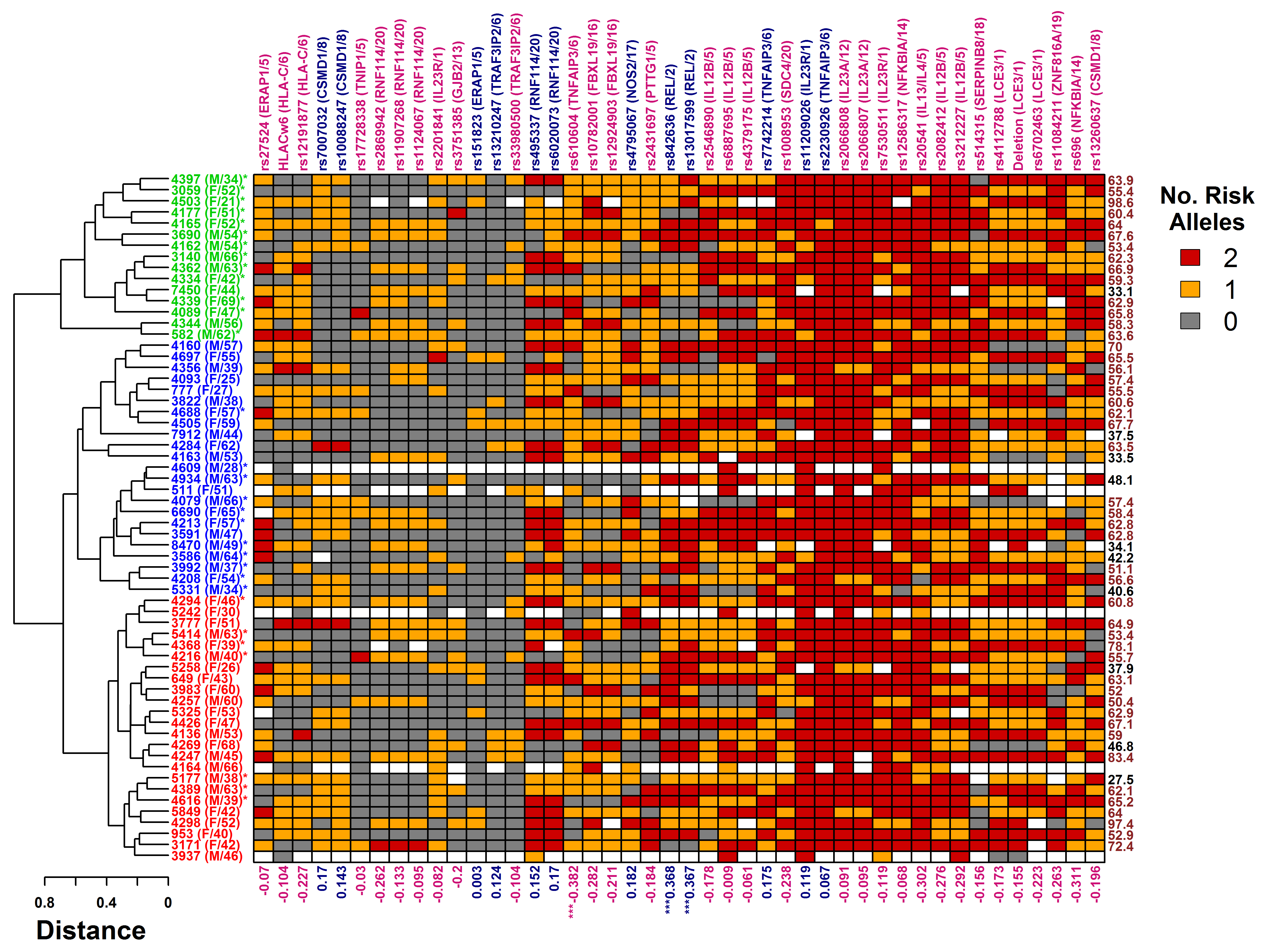

Supplement: Figure S13 — Frequency of risk alleles at 44 psoriasis susceptibility loci in 62 patients clustered according to inflammatory gene expression signatures. The 62 patients included in our cohort were clustered according to inflammatory gene expression signatures (see Figures 1 and S1; blue, red and green labels correspond to strong, moderate and weak sub-groups, respectively). Colors in the chart indicate whether subjects are homozygous for psoriasis risk alleles (red), heterozygous (orange) or non-carriers (grey), where each column corresponds to a different psoriasis susceptibility locus. Blank (white) regions represent cases where data is not available. Susceptibility loci (columns) have been clustered according to similarity across the 62 subjects (based on the Manhattan distance metric). For each locus, we evaluated whether risk alleles were more frequent in subjects associated with strong inflammatory patterns (blue labels) relative to those associated with weak inflammatory patterns (green labels). Estimates of Cliff's delta (Δ) are listed along the bottom margin of the chart (−1≤Δ≤1). Positive estimates (navy blue font) indicate a higher risk allele frequency in subjects with strong inflammatory patterns, and negative estimates (dark pink font) indicate lower risk allele frequency in subjects with strong inflammatory patterns. Significant estimates of Δ (P<0.05; prior to FDR adjustment) are denoted by three asterisk symbols (***). The right margin of the chart lists cumulative genetic risk scores calculated for each subject. Cumulative genetic risk scores were calculated by first fitting a logistic regression model, with risk allele burden (0, 1 or 2) at the 44 loci as predictors. The model was fit based upon marker data from an external training set of 2568 psoriasis cases and 2525 control subjects. This fitted logistic regression model was then applied to genotype data from our 62 subjects to calculate the genetic-based probability that a subject is a psoriasis case (tho [file pone.0034594.s013.tif]

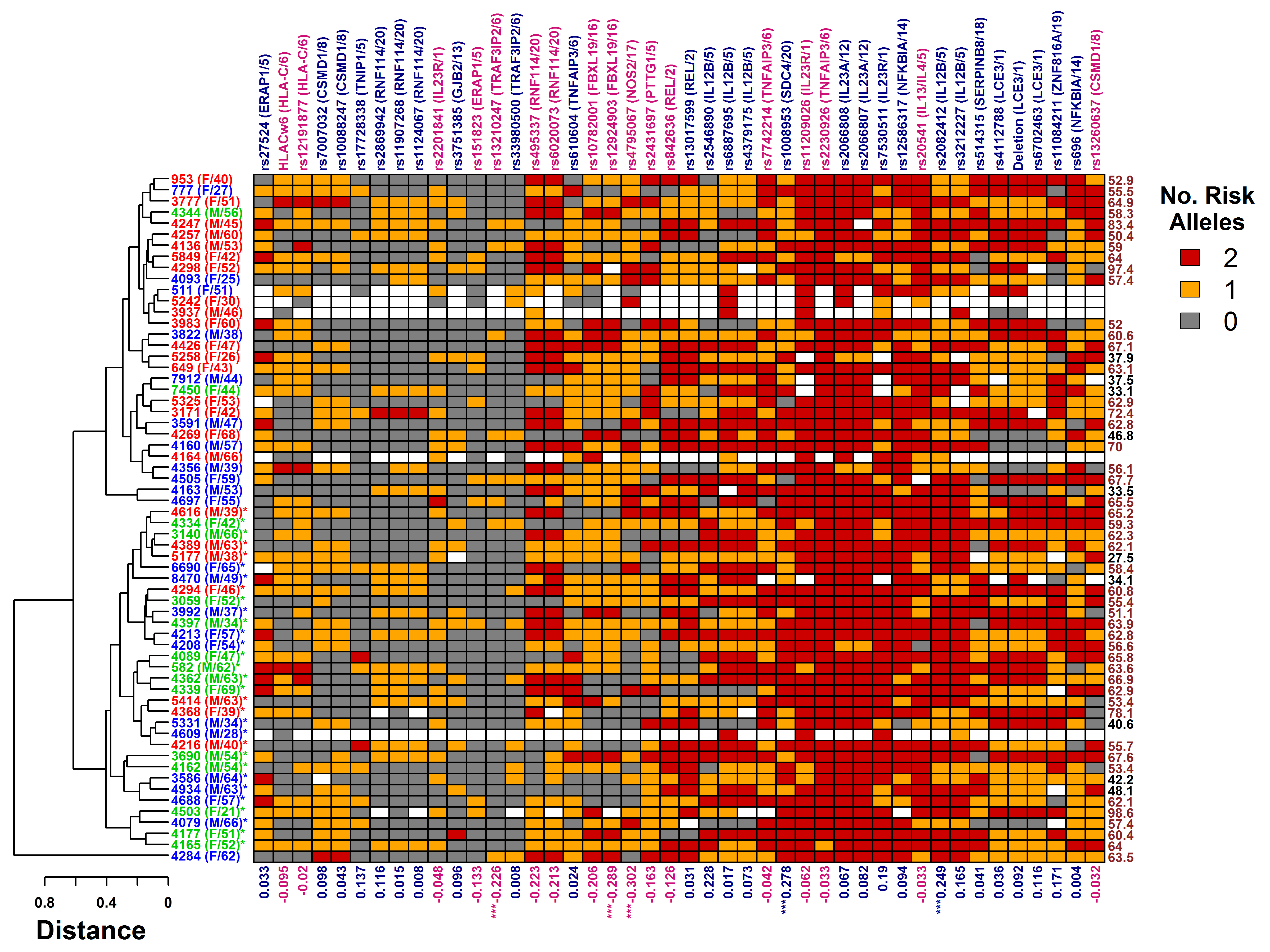

Supplement: Figure S14 — Frequency of risk alleles at 44 psoriasis susceptibility loci in 62 patients clustered according to cytokine-specific gene expression signatures. The 62 patients included in our cohort were clustered according to gene expression signatures representing the transcriptional response patterns of cultured keratinocytes treated with cytokines (see Figures 2 and S7; IL-13-weak subjects are denoted by an asterisk symbol). The interpretation of heatmap colors and calculated numerical values is consistent with Figure S13. However, in this figure, estimates of Cliff's delta (Δ) (−1≤Δ≤1) (bottom margin) were generated by testing whether risk allele frequency is elevated in IL-13-weak subjects (asterisk symbols). Positive values of Δ (navy blue) denote elevated risk allele frequency in subjects assigned to the IL-13-weak group, while negative values denote decreased risk allele frequency in subjects assigned to the IL-13-weak group. Significant estimates of Δ (P<0.05; prior to FDR adjustment) are indicated by three asterisk symbols (***). Cumulative genetic risk scores calculated for each subject are listed in the right margin (see Figure S13 legend for details). (TIF) [file pone.0034594.s014.tif]

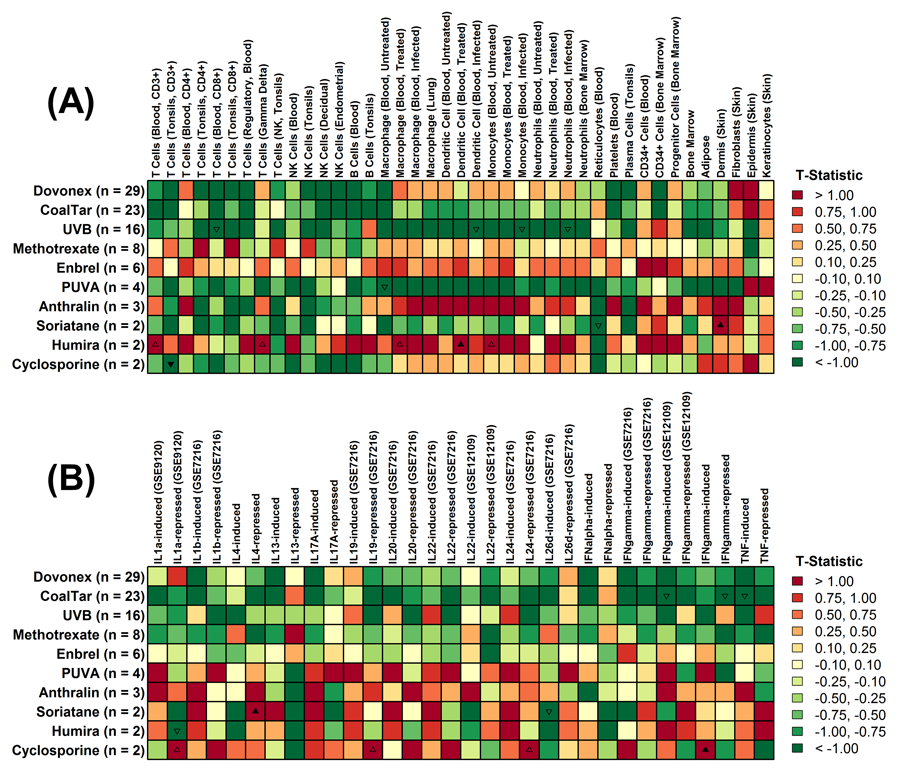

Supplement: Figure S15 — Psoriasis treatment history and its relationship with inflammatory and cytokine signatures. The 62 patients in our cohort were advised not to use systemic medications for at least 2 weeks prior to sample biopsies, and not to apply topical treatments for at least 1 week prior to biopsies. Patients completed a questionnaire in which they listed all therapies previously used to treat their condition. Ten prior treatments were reported among the 62 patients (left margin in A and B). For each treatment, a two-sample t-test was performed with respect to each inflammatory (part A) or cytokine (part B) signature in order to determine whether signature scores differed significantly among the n subjects reporting a given treatment history (compared with all other subjects that did not report the same treatment history). Colors correspond to the value of the T statistic generated from each two-sample t-test. Red colors denote a trend towards elevated signature scores among the n subjects reporting the treatment history listed in each row (see legend). Green colors denote a trend towards decreased signature scores among the n subjects reporting the treatment history listed in each row (see legend). Filled triangles denote significant T statistics based upon FDR-corrected p-values, while open triangles indicate significant T statistics based upon raw p-values prior to multiple test adjustment. (TIF) [file pone.0034594.s015.tif]
