# Supplementary material for: Heterogeneity of Inflammatory and Cytokine Networks in Chronic Plaque Psoriasis
Source: PLoS One. 2012 Mar 29;7(3):e34594. doi: 10.1371/journal.pone.0034594 (PMC3315545; doi:10.1371/journal.pone.0034594)
Supplement: Table S1 — Steady state mRNA levels of cytokines and their association with cytokine signature scores calculated from sets of cytokine-responsive transcripts. We identified 32 sets of 1000 cytokine-responsive transcripts in cultured keratinocytes (i.e., cytokine-induced or cytokine-repressed transcripts). For each set and each of 62 patients, we calculated a cytokine signature score, equal to the number of transcripts (of 1000) with higher expression in PP skin relative to PN skin, divided by the number of transcripts (of 1000) with lower expression in PP skin relative to PN skin. Additionally, for each set and each patient, we calculated the fold-change in steady state mRNA level between PP and PN skin. This table lists correlations between these fold-change estimates and cytokine signature scores across the 62 patients. (PDF) [file pone.0034594.s016.pdf]

Table S1. Steady state mRNA levels of cytokines and their association with cytokine signature scores calculated from sets of cytokine-responsive transcripts. We identified 32 sets of 1000 cytokine-responsive transcripts in cultured keratinocytes (i.e., cytokine-induced or cytokine-repressed transcripts). For each set and each of 62 patients, we calculated a cytokine signature score, equal to the number of transcripts (of 1000) with higher expression in PP skin relative to PN skin, divided by the number of transcripts (of 1000) with lower expression in PP skin relative to PN skin. Additionally, for each set and each patient, we calculated the fold-change in steady state mRNA level between PP and PN skin. The table lists correlations between these fold-change estimates and cytokine signature scores. Positive correlations indicate that, for patients with larger fold-change estimates (PP / PN) for a given cytokine, cytokine-responsive transcripts were more likely to be elevated in PP versus PN skin. Likewise, negative correlations indicate that, for patients with larger fold-change estimates (PP / PN) for a given cytokine, cytokine-responsive transcripts were more likely to have lower expression in PP versus PN skin.

Signature	Cytokine	Spearman r_s	P-value
IL1α-induced (GSE9120)	IL1α (210118_s_at)	0.101	0.434
IL1α-repressed (GSE9120)	IL1α (210118_s_at)	**-0.260**	**0.041**
IL1β-induced (GSE7216)	IL1β (205067_at)	0.194	0.131
IL1β-repressed (GSE7216)	IL1β (205067_at)	**-0.503**	**0.000**
IL4-induced	IL4 (207538_at)	**0.286**	**0.024**
IL4-repressed	IL4 (207538_at)	**-0.318**	**0.012**
IL13-induced	IL13 (207844_at)	**-0.660**	**0.000**
IL13-repressed	IL13 (207844_at)	**0.672**	**0.000**
IL17A-induced	IL17A (208402_at)	**-0.403**	**0.001**
IL17A-repressed	IL17A (208402_at)	-0.007	0.960
IL19-induced (GSE7216)	IL19 (220745_at)	0.186	0.147
IL19-induced (GSE7216)	IL19 (220745_at)	**-0.327**	**0.010**
IL20-induced (GSE7216)	IL20 (224071_at)	0.053	0.685
IL20-repressed (GSE7216)	IL20 (224071_at)	-0.177	0.169
IL22-induced (GSE7216)	IL22 (222974_at)	0.248	0.052
IL22-repressed (GSE7216)	IL22 (222974_at)	0.019	0.883
IL22-induced (GSE12109)	IL22 (222974_at)	0.083	0.520
IL22-repressed (GSE12109)	IL22 (222974_at)	-0.110	0.395
IL24-induced (GSE7216)	IL24 (206569_at)	0.077	0.553
IL24-repressed (GSE7216)	IL24 (206569_at)	**-0.374**	**0.003**
IL26d-induced (GSE7216)	IL26d (221111_at)	-0.126	0.329
IL26d-repressed (GSE7216)	IL26d (221111_at)	0.120	0.351
IFNα-induced	IFNα (208375_at)	**-0.355**	**0.005**
IFNα-repressed	IFNα (208375_at)	**0.352**	**0.005**
IFNγ-induced (GSE7216)	IFNγ (210354_at)	0.191	0.136
IFNγ-repressed (GSE7216)	IFNγ (210354_at)	**-0.280**	**0.028**
IFNγ-induced (GSE12109)	IFNγ (210354_at)	-0.024	0.854
IFNγ-repressed (GSE12109)	IFNγ (210354_at)	**-0.291**	**0.022**
IFNγ-induced	IFNγ (210354_at)	-0.011	0.931
IFNγ-repressed	IFNγ (210354_at)	**-0.261**	**0.040**
TNF-induced	TNF (207113_s_at)	0.113	0.382
TNF-repressed	TNF (207113_s_at)	-0.141	0.274
