# Supplementary material for: Heterogeneity of Inflammatory and Cytokine Networks in Chronic Plaque Psoriasis
Source: PLoS One. 2012 Mar 29;7(3):e34594. doi: 10.1371/journal.pone.0034594 (PMC3315545; doi:10.1371/journal.pone.0034594)
Supplement: Table S2 — Classification of 62 psoriasis lesions based upon genome-wide expression patterns. Psoriasis lesions from 62 patients were assigned to strong, moderate or weak inflammatory groups (Figures 1, S1 and S4), as well as to IL-13-strong or IL-13-weak groups (Figures 2, S7 and S9). This table lists the number of patients assigned to each of the inflammatory-cytokine group combinations. (PDF) [file pone.0034594.s017.pdf]

Table S2. Classification of 62 psoriasis lesions based upon genome-wide expression patterns. Psoriasis lesions from 62 patients were assigned to strong, moderate or weak inflammatory groups based upon the “signature transcripts” of immune cell populations and the altered expression of these transcripts in lesional (PP) versus non-lesional (PN) skin samples (Figures 1, S1 and S4). Likewise, psoriasis lesions were also assigned to IL-13-strong or IL-13-weak groups based upon transcripts responsive to cytokines in cultured keratinocytes and the altered expression of these transcripts in PP versus PN skin (Figures 2, S7 and S9). The table lists the number of patients assigned to each of the inflammatory-cytokine group combinations.

Inflammatory Groups	Cytokine Signature Groups	Number of Subjects (62)
Strong	IL-13-Strong	12 (19%)
Strong	IL-13-Weak	11 (18%)
Moderate	IL-13-Strong	17 (27%)
Moderate	IL-13-Weak	7 (11%)
Weak	IL-13-Strong	2 (3%)
Weak	IL-13-Weak	13 (21%)
