# Supplementary material for: Heterogeneity of Inflammatory and Cytokine Networks in Chronic Plaque Psoriasis
Source: PLoS One. 2012 Mar 29;7(3):e34594. doi: 10.1371/journal.pone.0034594 (PMC3315545; doi:10.1371/journal.pone.0034594)
Supplement: Table S3 — Set of 1000 signature transcripts with high expression in keratinocytes relative to normal skin. Heatmaps shown in Figures 1, S1 and S5 are based upon the expression patterns of cell type-specific “signature transcripts” in lesional (PP) and non-lesional (PN) skin samples. This table provides an example of the 1000 signature transcripts associated with one cell type (keratinocytes). (PDF) [file pone.0034594.s018.pdf]

Table S3. Set of 1000 signature transcripts with high expression in keratinocytes. Heatmaps shown in Figures 1, S1 and S5 are based upon expression patterns of cell type-specific signature transcripts in lesional (PP) and non-lesional (PN) skin samples. This table lists the 1000 signature transcripts associated with keratinocytes. Signature transcripts were identified based upon a two-sample comparison between RNA extracted from keratinocytes (n = 3) and RNA extracted from whole skin (n = 21). The three keratinocyte expression profiles were obtained from GEO series GSE7216 (samples GSM173541, GSM173542, and GSM173543). The 21 whole skin expression profiles were obtained from GEO series GSE7307, GSE6281, GSE16161 and GSE17539. Transcripts were identified using the following three steps. First, transcripts with higher expression on average in keratinocytes were identified. Second, the top 2000 of these transcripts with lowest p-values were isolated. Third, the top 1000 of these transcripts with largest fold-change were selected as signature transcripts (i.e., keratinocyte expression / whole skin expression). These 1000 transcripts are listed in the table below and are sorted according to the estimated fold-change ratio. The fourth column lists the raw p-values obtained from the test of differential expression between keratinocytes and whole skin. The fifth column lists Benjamini-Hochberg-adjusted p-values.

Gene Symbol	Probe Set ID	Fold-Change (Keratinocytes / Whole Skin)	P-value	Adjusted P-Value
S100A12	205863_at	211.15	3.45767E-09	5.04787E-07
S100A7A	232170_at	177.64	2.95737E-16	1.01059E-12
TCN1	205513_at	146.87	1.56117E-17	7.75974E-14
SERPINB4	211906_s_at	146.30	1.19392E-05	0.000290899
MMP1	204475_at	124.67	1.26075E-21	3.44657E-17
DEFB4	207356_at	96.23	5.32415E-09	7.13475E-07
AKR1B10	206561_s_at	85.25	2.41606E-12	1.78511E-09
PI3	203691_at	83.09	6.89551E-07	3.20589E-05
C10orf99	227736_at	61.99	6.94276E-08	5.44614E-06
PI3	41469_at	56.48	8.4459E-08	6.31709E-06
None	227735_s_at	46.40	2.38248E-10	6.31723E-08
KRT16	209800_at	44.26	2.85104E-08	2.63777E-06
SERPINB3	209720_s_at	42.55	1.20435E-05	0.000292788
HTR3A	216615_s_at	37.90	1.9267E-19	1.7557E-15
RPTN	1553454_at	37.84	8.18408E-09	1.00328E-06
GDA	224209_s_at	35.23	5.23084E-10	1.16259E-07
None	210413_x_at	33.79	3.20651E-06	0.000104169
ELF5	220625_s_at	33.58	3.50306E-11	1.4677E-08
IGF2BP3	203819_s_at	32.11	2.24068E-20	3.06272E-16
LCE3D	224328_s_at	30.75	1.92956E-06	7.08044E-05
KLK6	204733_at	30.74	8.17726E-08	6.15829E-06
SPINK7	223720_at	30.48	4.77714E-06	0.000141413
DSC2	204750_s_at	29.42	5.13943E-09	6.98145E-07

RNASE7	234699_at	27.92	2.11889E-11	9.95076E-09
SERPINB13	211361_s_at	27.76	3.04737E-07	1.68783E-05
HPSE	219403_s_at	27.43	1.00998E-09	1.91739E-07
IGF2BP3	203820_s_at	27.30	5.70457E-17	2.23913E-13
LCN2	212531_at	26.95	9.56548E-06	0.000243796
SERPINB3	209719_x_at	26.28	7.85241E-06	0.00021025
LIPG	219181_at	25.41	9.26827E-15	1.55522E-11
RNASE7	234700_s_at	24.75	2.87537E-08	2.65559E-06
S100P	204351_at	24.68	9.89742E-07	4.2322E-05
CCL20	205476_at	24.28	7.65772E-11	2.82896E-08
SERPINB13	216258_s_at	24.21	9.52685E-11	3.33898E-08
SPRR2C	220664_at	23.88	4.21873E-06	0.000128429
OLAH	219975_x_at	23.34	5.04215E-08	4.1833E-06
FGA	205649_s_at	22.81	8.29006E-24	4.53259E-19
SLC7A11	217678_at	22.74	3.48152E-13	3.7324E-10
SLC6A14	219795_at	22.08	5.49727E-06	0.000159281
NEFL	221805_at	22.08	5.69404E-17	2.23913E-13
CDH26	232306_at	21.96	6.73517E-20	7.36491E-16
None	208539_x_at	21.14	0.000135614	0.001868627
None	231033_at	21.07	2.069E-10	5.74226E-08
FGA	205650_s_at	20.65	4.56587E-15	9.1269E-12
None	1570192_at	20.47	9.31609E-15	1.55522E-11
SLC7A11	209921_at	19.40	3.76453E-13	3.81159E-10
SPINK6	1553973_a_at	19.25	1.0478E-12	9.39155E-10
WNT5A	205990_s_at	18.96	1.99326E-11	9.50565E-09
IL1A	210118_s_at	18.85	1.16676E-12	1.02378E-09
FGG	219612_s_at	18.85	8.18717E-15	1.50319E-11
DSC2	204751_x_at	18.79	2.3667E-08	2.25828E-06
AREG	205239_at	17.83	1.43983E-07	9.33839E-06
ELF5	220624_s_at	17.76	2.36504E-14	3.23272E-11
None	221305_s_at	17.42	1.33656E-10	4.12862E-08
ESYT3	1554912_at	17.10	1.87972E-08	1.93023E-06
SDR9C7	1553077_at	16.75	2.75407E-07	1.56202E-05
HTR3A	217002_s_at	16.73	5.23172E-12	3.40529E-09
SERPINB13	211362_s_at	16.72	7.44483E-10	1.49558E-07

OLAH	233126_s_at	16.17	1.28689E-12	1.06755E-09
SPRR3	232082_x_at	16.08	5.63466E-05	0.000957738
KLK12	233586_s_at	15.78	8.44765E-11	3.03892E-08
GJB2	223278_at	15.69	7.12957E-06	0.000195589
ZNF165	206683_at	15.39	1.84761E-11	9.01945E-09
ULBP2	238542_at	15.35	2.28759E-12	1.71334E-09
WNT5A	231227_at	15.27	8.94E-12	5.09161E-09
TGM1	206008_at	15.17	1.05256E-06	4.42001E-05
TREX2	211788_s_at	15.16	1.225E-10	3.94279E-08
SGPP2	244780_at	14.78	1.30534E-05	0.000310168
IL1F9	220322_at	14.56	9.40131E-07	4.08923E-05
MPZL2	203779_s_at	14.11	5.20308E-09	7.04154E-07
MAP2	210015_s_at	14.07	4.13372E-14	5.38121E-11
MMP10	205680_at	13.93	8.24798E-15	1.50319E-11
CDH26	233663_s_at	13.92	1.01059E-12	9.20905E-10
CD24	209772_s_at	13.90	5.64256E-09	7.50625E-07
CD24	208650_s_at	13.70	3.88907E-05	0.000721531
None	239736_at	13.45	1.77279E-16	6.46183E-13
HPGD	211549_s_at	13.32	2.65747E-09	4.08138E-07
WFDC12	1553081_at	13.30	1.29223E-07	8.61619E-06
PPIF	201490_s_at	13.29	2.42688E-10	6.37932E-08
SERPINB13	217272_s_at	13.29	3.28213E-06	0.000106121
NLRP10	1553534_at	12.92	1.71571E-15	3.90861E-12
SERPINA1	202833_s_at	12.85	3.79016E-06	0.000119028
SPRR1A	214549_x_at	12.85	9.76465E-05	0.001456914
SOD2	216841_s_at	12.70	7.73569E-09	9.54738E-07
ALOX12B	207381_at	12.65	4.16976E-06	0.000127721
HINT3	226537_at	12.64	1.40621E-06	5.55134E-05
TMEM45B	230323_s_at	12.61	1.33821E-08	1.47525E-06
None	229635_at	12.52	2.07351E-09	3.34422E-07
KLK10	209792_s_at	12.52	5.89216E-06	0.000167701
A2ML1	1564307_a_at	12.48	0.000123879	0.001739358
UPK1A	214624_at	12.47	2.61852E-06	8.96683E-05
APOB	205108_s_at	12.31	1.45267E-12	1.16236E-09
ATP11B	1554557_at	11.97	1.47601E-08	1.59173E-06

KRT17	205157_s_at	11.96	8.71455E-05	0.001337333
PTGS2	204748_at	11.96	1.09158E-05	0.000271141
RNASE7	233488_at	11.92	9.81565E-05	0.001461747
LYNX1	1554179_s_at	11.92	5.00123E-07	2.48185E-05
GDA	1569555_at	11.76	8.00369E-11	2.91735E-08
KLK13	205783_at	11.71	7.18796E-09	8.99318E-07
CASP14	231722_at	11.59	0.000136959	0.001881468
HIST1H2BC	214455_at	11.55	3.06657E-12	2.17747E-09
PKP1	205724_at	11.54	1.13958E-05	0.00028041
NAMPT	1555167_s_at	11.46	6.13865E-10	1.30596E-07
None	1562921_at	11.36	1.02341E-06	4.34097E-05
JMY	241985_at	11.35	6.5253E-09	8.37491E-07
NANOS1	228523_at	11.32	1.04528E-07	7.35305E-06
CHI3L2	213060_s_at	11.24	1.88872E-06	6.96801E-05
SGPP2	238567_at	11.21	2.99364E-07	1.66916E-05
SQLE	213562_s_at	11.08	7.18506E-07	3.31234E-05
WNT5A	213425_at	11.05	1.62718E-10	4.83511E-08
TLR2	204924_at	11.02	2.162E-10	5.88097E-08
KLK12	234316_x_at	11.01	1.4669E-12	1.16236E-09
MMP9	203936_s_at	10.93	1.21238E-10	3.94279E-08
OTUB2	222878_s_at	10.91	5.41986E-10	1.19008E-07
CHAC1	219270_at	10.91	1.98087E-07	1.21554E-05
HIGD1A	242317_at	10.86	6.99449E-08	5.47885E-06
HIST1H2BD	209911_x_at	10.67	1.33558E-10	4.12862E-08
PITX2	207558_s_at	10.46	1.28868E-12	1.06755E-09
None	208596_s_at	10.42	5.25526E-08	4.32728E-06
KLK12	220782_x_at	10.32	1.11715E-10	3.74726E-08
GGH	203560_at	10.16	2.64613E-06	9.04799E-05
ANKRD13C	1556361_s_at	10.15	6.90923E-10	1.42016E-07
CHRNA9	221107_at	10.15	1.92131E-10	5.4455E-08
SYTL5	242093_at	10.12	9.65309E-15	1.55522E-11
MPZL2	203780_at	9.98	1.18646E-06	4.85859E-05
PNP	201695_s_at	9.98	3.98981E-10	9.35361E-08
LYPD5	236039_at	9.78	7.50564E-07	3.41407E-05
KLK10	215808_at	9.77	1.29121E-10	4.08075E-08

ARSF	214490_at	9.72	1.54196E-10	4.63223E-08
None	215125_s_at	9.66	1.13742E-06	4.70411E-05
ZFY	230760_at	9.63	7.33793E-05	0.00117276
C12orf29	228378_at	9.63	2.68769E-06	9.15571E-05
FLVCR2	219316_s_at	9.51	9.66155E-11	3.36462E-08
CYP2C18	208126_s_at	9.41	5.1152E-09	6.97441E-07
ZDHHC21	241946_at	9.40	9.80182E-08	6.99627E-06
None	214580_x_at	9.39	0.000103667	0.001524189
GLRX	209276_s_at	9.39	1.52506E-05	0.000349882
GDPD3	219722_s_at	9.36	1.11029E-10	3.74724E-08
CYorf15A	236694_at	9.24	1.11492E-07	7.7358E-06
GM2A	215891_s_at	9.23	3.85277E-05	0.000716928
BNIP3	201848_s_at	9.20	1.29648E-08	1.44369E-06
OLAH	222945_x_at	9.19	1.16143E-08	1.32571E-06
None	238710_at	9.16	6.30748E-10	1.33667E-07
LOC344887	241418_at	9.11	9.17887E-08	6.70033E-06
MXD1	228846_at	9.07	6.28247E-06	0.000176241
FAM190B	1554132_a_at	9.04	2.00794E-10	5.60123E-08
LASS3	1554253_a_at	8.96	1.71727E-06	6.46191E-05
CHAC2	235117_at	8.95	7.22419E-10	1.46834E-07
HPGD	203913_s_at	8.92	9.12265E-05	0.001382431
GALNT6	219956_at	8.87	6.26957E-12	3.85156E-09
CAMSAP1L1	212763_at	8.86	6.75329E-12	4.05754E-09
CTH	217127_at	8.83	7.50428E-13	7.32672E-10
ABCG1	204567_s_at	8.78	5.42234E-06	0.000157779
WASL	205809_s_at	8.74	8.12887E-07	3.63406E-05
MLLT11	211071_s_at	8.72	2.52941E-07	1.45881E-05
PTGER3	210834_s_at	8.72	1.25514E-12	1.06755E-09
TWF1	214007_s_at	8.62	5.44891E-05	0.00093693
FAM190B	1554131_at	8.56	1.2196E-17	6.66815E-14
None	242951_at	8.53	2.79225E-10	7.20124E-08
RGS20	210138_at	8.51	3.51518E-10	8.39269E-08
HYAL4	220249_at	8.49	1.59288E-12	1.24415E-09
KRT6B	209126_x_at	8.47	8.90879E-06	0.000232057
EHF	219850_s_at	8.46	6.95037E-05	0.00112496

SCAMP1	206667_s_at	8.45	4.0189E-05	0.000738849
IL1F5	222223_s_at	8.44	1.0976E-05	0.00027216
A2ML1	1553505_at	8.44	2.79196E-08	2.5961E-06
HSPA4L	205543_at	8.42	3.28956E-05	0.000633299
TMEM45B	226226_at	8.40	2.06012E-09	3.33245E-07
PTGS2	1554997_a_at	8.40	3.02366E-06	9.97096E-05
None	1553575_at	8.33	8.25249E-05	0.001284168
AMMECR1	226421_at	8.26	1.26942E-06	5.12975E-05
TMPRSS11D	207602_at	8.26	1.76224E-07	1.09739E-05
C1orf116	219856_at	8.24	1.38404E-08	1.51953E-06
C12orf5	219099_at	8.23	6.04603E-08	4.83992E-06
FLVCR2	222866_s_at	8.22	1.07533E-17	6.53265E-14
PTGR1	228824_s_at	8.16	2.12958E-08	2.11316E-06
None	209093_s_at	8.13	5.70347E-13	5.66976E-10
None	240361_at	8.07	9.39786E-11	3.31502E-08
ARNTL2	220658_s_at	8.04	1.47112E-08	1.58959E-06
TMEM33	222642_s_at	8.03	2.18899E-07	1.30944E-05
FAM83A	238460_at	7.99	1.04236E-07	7.35305E-06
NETO2	222774_s_at	7.97	1.00866E-12	9.20905E-10
None	1565909_at	7.94	1.79088E-13	1.95832E-10
RHBDL2	1552502_s_at	7.93	3.60868E-06	0.000114379
RARRES1	221872_at	7.91	0.000104358	0.001528473
CDK5R1	204995_at	7.88	6.51406E-07	3.06715E-05
ARL5B	242727_at	7.87	6.52273E-10	1.3664E-07
XKRX	230349_at	7.84	4.24637E-07	2.17796E-05
SRXN1	225252_at	7.82	1.92224E-10	5.4455E-08
NQO1	210519_s_at	7.79	2.37321E-05	0.000493741
DSG3	205595_at	7.75	1.37678E-06	5.47457E-05
OAS1	205552_s_at	7.72	8.11602E-10	1.59947E-07
SLC7A5	201195_s_at	7.71	2.41378E-05	0.000499416
PRRC1	221734_at	7.71	5.21649E-08	4.30833E-06
RAB10	222981_s_at	7.68	1.67214E-08	1.76151E-06
TTRAP	202266_at	7.67	6.78847E-05	0.001105358
MBD1	241813_at	7.63	4.89001E-09	6.70078E-07
DIO2	203699_s_at	7.61	7.26623E-06	0.000198244

CYP2C18	215103_at	7.57	5.89274E-09	7.78226E-07
FGFBP1	205014_at	7.56	5.4839E-05	0.000939319
PPP2R2C	223573_s_at	7.54	7.19823E-10	1.46834E-07
SPCS3	218817_at	7.54	3.35187E-06	0.000107866
SLC6A15	206376_at	7.54	1.49119E-14	2.26475E-11
AVPI1	218631_at	7.54	1.7787E-10	5.22852E-08
TMEM86A	242103_at	7.52	8.46105E-10	1.65217E-07
STK17A	202693_s_at	7.52	6.18787E-08	4.90321E-06
G6PD	202275_at	7.52	9.46111E-08	6.79746E-06
FBXO45	225100_at	7.51	4.36193E-06	0.000131907
OASL	205660_at	7.47	4.80885E-10	1.07756E-07
AADAC	205969_at	7.47	7.82463E-07	3.52398E-05
SOD2	215223_s_at	7.46	2.16947E-08	2.12573E-06
PTP4A1	200730_s_at	7.36	0.000108162	0.001570304
OTUB2	219369_s_at	7.35	6.90879E-09	8.76422E-07
SERPINA1	211429_s_at	7.32	3.35292E-05	0.000642628
SLC39A6	1556551_s_at	7.31	2.03899E-07	1.24152E-05
AMMECR1	1553219_a_at	7.30	4.47917E-08	3.83253E-06
None	209372_x_at	7.29	5.30619E-08	4.3482E-06
SERPINB11	1552463_at	7.27	9.00999E-08	6.62125E-06
PAWR	204005_s_at	7.26	0.000114842	0.001641998
CD24	266_s_at	7.23	1.35949E-05	0.000320111
None	240277_at	7.23	8.58423E-06	0.000226298
SDCBP2	233565_s_at	7.22	1.37153E-10	4.21282E-08
FBN2	203184_at	7.20	3.0216E-10	7.57826E-08
VNN3	220528_at	7.15	7.6299E-09	9.43812E-07
FBXL20	235089_at	7.12	6.825E-09	8.6983E-07
ATP12A	207367_at	7.08	4.94268E-08	4.11953E-06
GM2A	33646_g_at	7.08	8.73377E-05	0.001338994
HMGA2	208025_s_at	7.07	1.92611E-09	3.17199E-07
PTGER3	211909_x_at	7.07	1.04617E-06	4.39995E-05
NETO2	218888_s_at	7.07	2.14538E-10	5.86493E-08
C1orf116	228865_at	7.06	6.50003E-07	3.0637E-05
C16orf72	1568954_s_at	7.06	5.65573E-06	0.000162665
ANXA9	210085_s_at	7.05	1.7446E-05	0.000389013

None	235230_at	7.05	4.21607E-06	0.000128429
LOC100286920	236213_at	7.03	1.50377E-08	1.61213E-06
NAMPT	217739_s_at	7.01	8.10841E-08	6.11521E-06
MAP2	225540_at	6.97	3.73489E-05	0.000699093
PHACTR2	244774_at	6.97	6.60396E-07	3.09667E-05
PREPL	212216_at	6.95	2.76211E-06	9.33144E-05
ERO1L	222646_s_at	6.94	1.06398E-06	4.44747E-05
DLX2	207147_at	6.90	5.5474E-14	6.7401E-11
DPH3	225195_at	6.88	1.85494E-06	6.86189E-05
ATP11B	1564064_a_at	6.88	1.68127E-08	1.76151E-06
SEC14L1	202083_s_at	6.87	7.04423E-09	8.89476E-07
BNIP3	201849_at	6.83	2.30577E-07	1.35121E-05
CDA	205627_at	6.81	2.40368E-08	2.27767E-06
None	237563_s_at	6.78	3.03378E-07	1.68453E-05
CAMSAP1L1	217196_s_at	6.77	5.9351E-05	0.000995404
NEFM	205113_at	6.77	1.99936E-11	9.50565E-09
RPL27A	223707_at	6.75	1.21029E-11	6.36277E-09
OBFC2A	233085_s_at	6.75	4.73254E-07	2.37169E-05
CARHSP1	218384_at	6.75	8.02454E-08	6.07676E-06
ZNF770	220608_s_at	6.72	2.57824E-07	1.48541E-05
CLCN3	201733_at	6.70	9.35434E-07	4.07204E-05
None	207126_x_at	6.70	1.37303E-06	5.4716E-05
EHF	224189_x_at	6.67	2.57904E-05	0.000528124
ASPHD2	227014_at	6.65	1.20474E-10	3.94279E-08
SLC39A6	1555460_a_at	6.60	8.11184E-05	0.001268634
ARL11	1552691_at	6.58	3.98196E-08	3.47231E-06
CD24	208651_x_at	6.58	3.65591E-06	0.000115675
SPTLC2	216202_s_at	6.56	1.25463E-09	2.27896E-07
SFT2D2	214838_at	6.56	5.73347E-17	2.23913E-13
None	204532_x_at	6.56	2.56102E-06	8.82316E-05
IRAK2	231779_at	6.45	9.09729E-12	5.12778E-09
SNAP29	218327_s_at	6.44	2.22108E-10	6.01177E-08
NFE2L3	204702_s_at	6.44	8.13864E-05	0.001271009
None	231211_s_at	6.43	1.04364E-07	7.35305E-06
IVL	214599_at	6.42	8.58246E-06	0.000226298

PSPH	205194_at	6.40	3.1458E-09	4.67972E-07
SLC7A8	216604_s_at	6.39	1.83994E-09	3.0719E-07
SERPINB8	206034_at	6.38	9.02327E-08	6.62211E-06
None	206302_s_at	6.38	4.49431E-05	0.00080512
GBAP1	210589_s_at	6.37	2.50106E-09	3.90702E-07
PPIF	201489_at	6.36	1.6497E-07	1.03795E-05
ELMOD2	1553928_at	6.36	5.16418E-05	0.000896638
SAR1B	218254_s_at	6.35	3.69049E-06	0.0001165
FAM40B	231880_at	6.34	3.11442E-11	1.3626E-08
AMDHD1	229596_at	6.34	2.25827E-08	2.19309E-06
CLCN3	201735_s_at	6.33	1.75371E-06	6.5719E-05
DUOXA1	1554648_a_at	6.32	1.83272E-06	6.7981E-05
CDKN2B	236313_at	6.30	4.96329E-05	0.000869974
PDZK1IP1	219630_at	6.29	5.37749E-07	2.62982E-05
HSD17B2	204818_at	6.29	1.29628E-05	0.000308807
UBE2H	221962_s_at	6.28	9.34897E-12	5.21587E-09
ODC1	200790_at	6.28	5.66708E-09	7.52057E-07
LPHN2	206953_s_at	6.28	4.20802E-06	0.000128429
PPP2R2C	223574_x_at	6.27	3.04999E-07	1.68783E-05
HEATR7A	230361_at	6.27	5.09032E-11	2.03148E-08
LMO7	242722_at	6.24	1.60005E-05	0.000362999
SCYL2	221220_s_at	6.22	7.22792E-06	0.000197494
None	230734_x_at	6.21	3.524E-05	0.00066699
HIST1H2AC	215071_s_at	6.21	6.67487E-06	0.000185914
PTGER3	210833_at	6.20	0.000144196	0.001958047
SMPD3	231732_at	6.20	1.21845E-07	8.26533E-06
SQLE	213577_at	6.20	5.31521E-05	0.00091965
KRT78	1553213_a_at	6.18	0.000149283	0.002010855
NEFL	221916_at	6.15	3.69147E-13	3.80814E-10
ZNF426	205964_at	6.15	1.05504E-07	7.40494E-06
PDZK1IP1	1553589_a_at	6.14	1.40624E-06	5.55134E-05
USP38	223288_at	6.13	5.72347E-05	0.000967627
ARNTL2	224204_x_at	6.11	1.73175E-08	1.80693E-06
STEAP4	220187_at	6.08	1.21711E-05	0.000295234
PLBD1	218454_at	6.08	7.46764E-10	1.49558E-07

NAMPT	217738_at	6.08	8.44239E-06	0.000223421
NOD2	220066_at	6.06	2.91296E-08	2.68577E-06
TYMP	204858_s_at	6.06	7.45872E-06	0.000202184
None	238213_at	6.06	9.37082E-14	1.09011E-10
KRT17	212236_x_at	6.06	9.16443E-05	0.001387223
NRBF2	221803_s_at	6.05	1.01635E-05	0.000256195
REEP3	235016_at	6.03	4.70573E-05	0.000832639
ATP11B	1554556_a_at	5.99	3.15234E-10	7.87005E-08
ARL8B	222442_s_at	5.96	1.66033E-05	0.000373729
USP38	223289_s_at	5.96	4.13135E-05	0.000755204
C4orf19	219450_at	5.96	2.83472E-08	2.63138E-06
STIP1	212009_s_at	5.96	5.95594E-07	2.8565E-05
SLC19A2	209681_at	5.92	1.53939E-05	0.000351981
None	239825_at	5.92	8.38609E-16	2.08413E-12
PYDC1	243722_at	5.92	1.66478E-06	6.29473E-05
None	241044_x_at	5.92	5.82436E-05	0.000980741
DSC2	226817_at	5.91	3.9933E-08	3.47665E-06
TMC5	240304_s_at	5.91	1.91369E-05	0.000418215
KYNU	217388_s_at	5.90	1.78724E-05	0.000396581
NQO1	201467_s_at	5.90	7.24082E-07	3.33523E-05
PGD	201118_at	5.89	3.06224E-08	2.78583E-06
TBL1X	201867_s_at	5.89	1.68047E-08	1.76151E-06
ZDHHC21	243550_at	5.87	5.87235E-12	3.64853E-09
ELL2	226099_at	5.87	1.08257E-11	5.80289E-09
UPP1	203234_at	5.86	3.44484E-07	1.85563E-05
RALGPS2	242458_at	5.85	1.27057E-06	5.13059E-05
CYP51A1	216607_s_at	5.82	1.16527E-06	4.80477E-05
None	208579_x_at	5.81	1.38683E-08	1.51953E-06
GLRX	206662_at	5.79	4.20018E-06	0.000128429
SMPD3	219695_at	5.78	3.91195E-06	0.000121803
DIO2	231240_at	5.77	2.22629E-05	0.000470134
ELL2	214446_at	5.76	4.98876E-07	2.48185E-05
SPCS3	222753_s_at	5.74	2.05318E-08	2.05224E-06
KCTD4	239787_at	5.73	7.82465E-08	5.97504E-06
PGM2	225366_at	5.72	5.58754E-06	0.000160873

UBE2F	225783_at	5.71	6.56338E-09	8.40404E-07
CDV3	213548_s_at	5.71	5.70006E-05	0.000964565
None	206094_x_at	5.70	1.48671E-06	5.76598E-05
OAS1	202869_at	5.69	1.96148E-09	3.20132E-07
AREGB	1557285_at	5.69	1.36983E-07	9.01271E-06
GPX2	202831_at	5.68	4.3577E-06	0.000131907
NT5C3	223298_s_at	5.68	5.71978E-07	2.77981E-05
None	230659_at	5.68	6.16075E-06	0.000173897
FCHSD1	226698_at	5.66	1.43478E-07	9.31669E-06
EPRS	200841_s_at	5.66	5.8989E-06	0.000167806
ASCC3	212815_at	5.65	3.60442E-05	0.000679089
ULBP2	221291_at	5.65	1.6516E-10	4.88114E-08
SAR1B	1554482_a_at	5.63	3.19485E-07	1.74679E-05
SLC31A1	203971_at	5.63	3.30143E-05	0.000634913
PTGR1	231897_at	5.62	1.77209E-09	2.9904E-07
NLRP2	221690_s_at	5.62	7.42989E-06	0.000201507
FAM43A	227410_at	5.61	6.10358E-08	4.85893E-06
HIST1H2BD	222067_x_at	5.61	7.93207E-11	2.91064E-08
TTC39A	210652_s_at	5.61	3.73695E-06	0.00011783
SLC45A4	225598_at	5.59	2.12882E-10	5.84892E-08
SLC16A1	209900_s_at	5.59	1.39329E-08	1.52356E-06
ME1	204059_s_at	5.58	4.51169E-06	0.000135686
RIT1	209882_at	5.58	2.19998E-07	1.31314E-05
GK3P	215966_x_at	5.57	7.6097E-09	9.43447E-07
GSDMC	234305_s_at	5.54	1.07683E-10	3.67973E-08
RAB5A	206113_s_at	5.54	1.76943E-05	0.000393106
DCTN5	209231_s_at	5.54	9.15397E-08	6.69519E-06
CD24	216379_x_at	5.53	5.08877E-05	0.000886642
POF1B	1555382_at	5.52	6.04675E-06	0.000171298
SQLE	1557352_at	5.52	1.05033E-10	3.63462E-08
CDH3	203256_at	5.52	3.51733E-06	0.000111938
INA	204465_s_at	5.50	3.38732E-10	8.19476E-08
CD177	219669_at	5.48	8.29146E-10	1.62486E-07
KLK13	217315_s_at	5.47	1.43348E-05	0.000333372
CLCN3	201732_s_at	5.47	1.4154E-06	5.57545E-05

SLC5A1	206628_at	5.46	2.04139E-07	1.24152E-05
GK	207387_s_at	5.46	3.97993E-07	2.07636E-05
C9orf85	238912_x_at	5.44	4.35974E-08	3.75976E-06
SOX2	228038_at	5.43	3.3841E-07	1.83376E-05
IGFL1	239430_at	5.43	1.11389E-08	1.28756E-06
ATP11B	238811_at	5.42	9.43181E-08	6.79033E-06
SLC7A11	207528_s_at	5.42	7.7807E-09	9.5813E-07
PRSS3	207463_x_at	5.39	3.96789E-05	0.000731438
HIST1H2BH	208546_x_at	5.38	2.52854E-09	3.93868E-07
ELAVL2	228260_at	5.36	8.70511E-08	6.46674E-06
None	231042_s_at	5.35	1.94298E-08	1.98195E-06
NFE2L3	236471_at	5.34	3.38642E-05	0.000647159
SLC45A4	225597_at	5.33	5.32485E-08	4.35181E-06
NLRX1	1553695_a_at	5.33	2.85125E-08	2.63777E-06
HMOX1	203665_at	5.32	2.78429E-08	2.5961E-06
AMMECR1	204976_s_at	5.31	3.03647E-05	0.000597456
ATP13A4	1557136_at	5.30	4.00895E-06	0.000124046
None	200776_s_at	5.29	0.00011242	0.00161837
UBE2F	231948_s_at	5.29	3.75043E-07	1.99276E-05
BICD2	1553021_s_at	5.29	7.50237E-07	3.41407E-05
GPLD1	206265_s_at	5.28	1.86314E-09	3.08439E-07
ATP6V1D	208899_x_at	5.27	7.29939E-10	1.47267E-07
STRN	205520_at	5.25	1.32319E-12	1.07978E-09
None	1560848_at	5.25	5.3185E-10	1.17728E-07
RBMS1	225265_at	5.24	1.44338E-05	0.000334937
STAT3	208992_s_at	5.24	8.56737E-10	1.66698E-07
UBE2F	225787_at	5.24	5.34466E-07	2.6208E-05
TLCD2	1557275_a_at	5.23	2.2844E-06	8.08934E-05
PTP4A1	200733_s_at	5.23	5.36359E-07	2.62773E-05
SLC2A1	201250_s_at	5.23	3.11337E-06	0.000101869
LRG1	228648_at	5.23	0.000126063	0.001764142
RMND5A	212479_s_at	5.22	6.87614E-08	5.40577E-06
GPT2	224839_s_at	5.21	1.27276E-05	0.000304811
CD24	209771_x_at	5.19	5.24577E-05	0.000909649
KCNJ15	210119_at	5.19	1.01426E-07	7.17397E-06

NADSYN1	232946_s_at	5.17	4.45122E-09	6.20843E-07
EHF	222932_at	5.15	3.74332E-05	0.000700191
DFNA5	203695_s_at	5.14	9.00157E-07	3.9531E-05
DUSP14	203367_at	5.14	2.75042E-05	0.000553884
TRIM16	204341_at	5.10	2.26616E-06	8.0404E-05
EPPK1	208156_x_at	5.10	1.67426E-05	0.000376245
HIST1H4H	208180_s_at	5.09	1.20919E-08	1.36879E-06
None	1559877_at	5.07	2.94445E-08	2.70567E-06
ATP6V1D	208898_at	5.07	4.00319E-10	9.35361E-08
C9orf84	233504_at	5.06	5.3891E-11	2.13514E-08
IGFBP2	202718_at	5.05	2.76434E-05	0.000555867
TMOD3	223078_s_at	5.04	3.4004E-05	0.000649605
ERC2	213938_at	5.03	1.48411E-14	2.26475E-11
LAMC2	202267_at	5.03	2.26915E-06	8.04577E-05
ATP11B	1564063_a_at	5.02	1.1553E-05	0.000283421
None	233413_at	5.02	9.90029E-07	4.2322E-05
PPPDE2	212527_at	5.01	7.79751E-08	5.96264E-06
SLPI	203021_at	4.98	5.53272E-05	0.000945022
ZDHHC21	235068_at	4.98	2.15866E-08	2.12275E-06
CPA4	205832_at	4.98	3.19875E-05	0.000619744
TMEM54	225536_at	4.97	6.54495E-07	3.0733E-05
KLHL18	1557165_s_at	4.97	1.66455E-08	1.76034E-06
WWTR1	202134_s_at	4.97	2.83414E-10	7.27497E-08
TNPO1	225765_at	4.96	2.98295E-07	1.66591E-05
CDV3	213554_s_at	4.96	1.08536E-10	3.68586E-08
ARG2	203946_s_at	4.95	7.26693E-08	5.63791E-06
PTPN22	206060_s_at	4.95	1.5654E-11	7.92486E-09
CCND2	200951_s_at	4.95	1.46188E-06	5.71058E-05
TMC5	240303_at	4.95	1.9379E-10	5.46158E-08
LRRC20	218550_s_at	4.94	7.25352E-08	5.63791E-06
CFLAR	211317_s_at	4.92	0.0001382	0.001889962
PRSS3	213421_x_at	4.91	2.61637E-05	0.000533571
KIAA1239	230765_at	4.90	9.25058E-11	3.28426E-08
SPATA5	229075_at	4.90	1.99618E-05	0.000432243
STYK1	220030_at	4.90	1.19917E-09	2.20756E-07

OBFC2A	222872_x_at	4.90	1.51741E-06	5.85495E-05
PTPN22	236539_at	4.89	4.08866E-12	2.82971E-09
FAM108B1	220285_at	4.89	2.153E-08	2.12275E-06
HIST1H4H	232035_at	4.88	1.17411E-08	1.33739E-06
ENDOD1	212570_at	4.86	2.55851E-10	6.66127E-08
TLE3	206472_s_at	4.86	6.60442E-09	8.43684E-07
HEATR3	1554478_a_at	4.84	2.56479E-05	0.000525797
TMC5	222904_s_at	4.84	3.46413E-10	8.34366E-08
ASNS	205047_s_at	4.83	1.25654E-07	8.48167E-06
TMEM49	1569003_at	4.82	3.70071E-06	0.000116755
NRBF2	223650_s_at	4.81	1.9103E-05	0.00041779
RALGPS2	232112_at	4.80	2.27197E-06	8.05056E-05
LOC100131262	1558152_at	4.79	9.21094E-08	6.71478E-06
None	235456_at	4.79	2.99791E-06	9.92196E-05
LIG4	227766_at	4.76	1.02749E-05	0.000258409
RHBDL2	1554897_s_at	4.76	1.69097E-07	1.06222E-05
RAB27B	207018_s_at	4.76	3.36881E-06	0.000108219
KCTD4	240512_x_at	4.75	1.45383E-08	1.57403E-06
ORMDL2	218556_at	4.75	1.19314E-09	2.20756E-07
AP1S3	1555731_a_at	4.75	5.23309E-08	4.31553E-06
TP53I3	210609_s_at	4.74	6.03914E-08	4.83992E-06
CTSC	225646_at	4.73	1.271E-05	0.000304522
PHLDA1	217996_at	4.72	0.000116011	0.001653595
MOBKL1B	201298_s_at	4.71	3.46874E-05	0.000658977
N4BP1	32069_at	4.71	6.6886E-08	5.28467E-06
None	239858_at	4.69	3.97293E-07	2.07636E-05
ATXN3	205416_s_at	4.69	1.65982E-08	1.75873E-06
CISD2	226686_at	4.68	3.85981E-05	0.000717319
TLCD2	241359_at	4.68	1.59658E-07	1.01034E-05
HIST1H2BK	209806_at	4.68	2.1292E-07	1.27928E-05
PGLYRP4	220944_at	4.68	5.43812E-08	4.41797E-06
SPRR4	1552620_at	4.68	0.00014781	0.001994949
TMEM86A	227570_at	4.67	2.39251E-08	2.27101E-06
RHOD	209885_at	4.67	2.03744E-05	0.000438351
ITCH	209744_x_at	4.65	4.68123E-11	1.8959E-08

HPSE	222881_at	4.65	2.27136E-05	0.000476541
CYP4F22	244692_at	4.65	9.80078E-05	0.0014605
GAB1	207112_s_at	4.64	2.35716E-08	2.25828E-06
ZBTB7A	213303_x_at	4.64	6.85354E-08	5.39939E-06
STYK1	221696_s_at	4.63	4.97002E-10	1.10913E-07
SHC1	201469_s_at	4.63	2.07984E-05	0.000445942
TJP2	202085_at	4.63	1.17344E-05	0.000287061
VWA1	222723_at	4.62	1.33737E-07	8.84167E-06
C10orf118	229399_at	4.62	9.79277E-07	4.20597E-05
None	216470_x_at	4.60	1.26587E-07	8.49223E-06
ITCH	209743_s_at	4.60	1.93783E-11	9.37619E-09
IL1F8	231755_at	4.59	5.09726E-12	3.39869E-09
INPP4B	205376_at	4.59	4.25598E-07	2.18084E-05
SH3D20	1554594_at	4.59	6.71952E-08	5.30144E-06
CDKN2B	207530_s_at	4.56	3.14819E-07	1.73167E-05
TNFRSF21	214581_x_at	4.55	4.19724E-06	0.000128418
PGM2	223738_s_at	4.55	3.2497E-10	7.98867E-08
EPN3	220318_at	4.54	3.44138E-06	0.000109932
HIGD1A	221896_s_at	4.54	7.413E-09	9.23248E-07
BLZF1	32088_at	4.53	1.75583E-05	0.000390721
SH2D5	230973_at	4.52	4.39378E-08	3.78315E-06
ATP10B	214070_s_at	4.52	9.34802E-08	6.76957E-06
PTHLH	211756_at	4.52	3.04319E-09	4.55853E-07
SLC16A1	202234_s_at	4.50	1.74665E-05	0.000389312
None	1568812_at	4.50	2.88959E-11	1.29499E-08
CLN8	222874_s_at	4.49	1.23699E-05	0.00029838
None	227356_at	4.49	0.000106451	0.001551235
ETNK1	222262_s_at	4.47	3.22318E-06	0.000104648
TGFA	205016_at	4.47	3.05151E-05	0.000600148
PRDM1	217192_s_at	4.47	5.2933E-09	7.11084E-07
PPP2R1B	202886_s_at	4.47	4.14179E-05	0.000756354
LARP4	214155_s_at	4.47	8.41473E-05	0.001304438
DNAJB6	209015_s_at	4.47	1.98166E-06	7.22585E-05
ZNF589	210061_at	4.46	3.54342E-11	1.4677E-08
PTGR1	228825_at	4.46	3.4007E-06	0.000108918

None	236451_at	4.45	3.03639E-07	1.68453E-05
None	239452_at	4.45	2.84192E-11	1.29485E-08
TOP1	208900_s_at	4.44	5.39628E-05	0.000931907
PPIL1	222500_at	4.44	0.000111552	0.001607573
ABCG4	207593_at	4.43	2.12938E-11	9.95076E-09
PAQR5	242871_at	4.42	2.22352E-06	7.90962E-05
ISL1	206104_at	4.40	7.40085E-08	5.72336E-06
LOC283404	1558195_at	4.40	5.73398E-10	1.24407E-07
ZFAND6	221613_s_at	4.38	1.83055E-06	6.79696E-05
FAM108B1	227551_at	4.38	2.1526E-05	0.000458487
TRIM25	206911_at	4.38	2.42012E-11	1.12136E-08
ATP6V1C1	202872_at	4.37	4.12897E-05	0.000755182
SYNCRIP	217834_s_at	4.37	3.43115E-06	0.000109706
OCLN	209925_at	4.36	4.36176E-06	0.000131907
LARP4B	228196_s_at	4.36	1.21698E-05	0.000295234
AGFG1	213926_s_at	4.35	8.9427E-07	3.93357E-05
MTHFD1L	225520_at	4.34	1.33828E-05	0.000315936
UBQLN1	222989_s_at	4.34	2.26682E-05	0.000475954
None	1567076_at	4.34	1.07677E-05	0.000267968
NPR3	219789_at	4.33	4.98885E-06	0.000146412
UBE2D1	214590_s_at	4.32	1.99636E-07	1.22367E-05
NLRX1	219680_at	4.32	3.29248E-08	2.96567E-06
GM2A	209727_at	4.32	1.00352E-07	7.12568E-06
KCTD11	235857_at	4.31	1.888E-06	6.96801E-05
SMOX	210357_s_at	4.31	3.00842E-07	1.67501E-05
EIF5	208708_x_at	4.31	7.22228E-05	0.00115936
EIF4E	201437_s_at	4.31	9.14038E-05	0.00138435
SPIRE1	1554807_a_at	4.30	4.5719E-06	0.000136722
TMEM40	222892_s_at	4.30	2.02297E-05	0.0004364
None	219095_at	4.29	1.34006E-07	8.84878E-06
None	227278_at	4.29	3.35384E-05	0.000642628
ADAP2	222876_s_at	4.26	2.26098E-07	1.33354E-05
C18orf19	235022_at	4.26	7.64344E-07	3.46809E-05
ACRV1	207973_x_at	4.26	5.96696E-10	1.27939E-07
TAF13	205966_at	4.25	5.73345E-12	3.64853E-09

SPIRE1	1559517_a_at	4.25	4.96039E-08	4.128E-06
CDK7	211297_s_at	4.25	9.83998E-06	0.000249768
SLC30A1	228181_at	4.24	8.8585E-05	0.001352901
USP2	207213_s_at	4.24	4.0184E-05	0.000738849
FUT2	210608_s_at	4.23	3.21654E-05	0.000622087
AGFG1	218092_s_at	4.23	3.82691E-05	0.000713386
GJA3	239572_at	4.23	1.45459E-11	7.43271E-09
SPTLC2	203127_s_at	4.22	3.48913E-11	1.4677E-08
RARRES1	206392_s_at	4.22	0.000131754	0.001828338
TGFA	205015_s_at	4.22	6.05525E-08	4.84021E-06
PCSK6	210553_x_at	4.21	5.14591E-09	6.98145E-07
TSLP	235737_at	4.21	1.44268E-07	9.34577E-06
SLC7A8	216603_at	4.20	9.31527E-08	6.76201E-06
GM2A	35820_at	4.20	1.12677E-05	0.000277755
TWF1	214008_at	4.20	9.9641E-08	7.08436E-06
ADAP2	219358_s_at	4.20	6.92472E-07	3.21673E-05
AKIRIN1	222458_s_at	4.20	3.85893E-05	0.000717319
ACRV1	208013_s_at	4.20	1.19955E-08	1.36069E-06
GK	214681_at	4.19	2.7934E-07	1.57941E-05
RASAL1	219752_at	4.19	4.3792E-05	0.000789686
SLAMF7	222838_at	4.19	1.44147E-05	0.00033466
SLC2A1	201249_at	4.19	4.14484E-10	9.6025E-08
MRPL43	230026_at	4.18	9.66377E-05	0.001445995
SLC5A1	242773_at	4.17	3.6715E-07	1.95652E-05
SPIRE1	225018_at	4.17	3.69001E-06	0.0001165
ACAP2	1552472_a_at	4.16	9.15958E-08	6.69519E-06
PSENEN	218302_at	4.16	0.000102546	0.001512062
TARS	201263_at	4.16	3.00384E-08	2.75101E-06
TBC1D12	1557609_s_at	4.16	1.84286E-09	3.0719E-07
MID2	208384_s_at	4.16	8.13263E-10	1.59947E-07
HIST1H2BF	208490_x_at	4.16	9.37349E-10	1.80456E-07
None	236422_at	4.16	1.20905E-05	0.00029374
DHRS1	213279_at	4.15	9.48188E-06	0.000242423
TUBBP5	222361_at	4.15	1.59598E-06	6.11066E-05
None	1561670_at	4.13	2.0085E-08	2.01865E-06

PCP4L1	241382_at	4.13	3.23439E-05	0.000624436
RASEF	1553986_at	4.13	2.51063E-06	8.69341E-05
UBE2G1	209142_s_at	4.13	2.32275E-06	8.20389E-05
MXD1	206877_at	4.13	9.5482E-06	0.000243656
None	231655_x_at	4.12	4.01243E-06	0.000124083
TOX4	217448_s_at	4.12	9.85266E-05	0.001465972
C6orf141	1554314_at	4.12	4.53634E-08	3.85131E-06
FAM83A	238741_at	4.12	2.85138E-05	0.00056856
C2orf49	219662_at	4.12	1.23789E-10	3.94279E-08
KCNK1	204678_s_at	4.11	1.05717E-06	4.43258E-05
GARS	208693_s_at	4.10	2.90748E-09	4.37924E-07
LOC100129895	1560290_at	4.10	2.2344E-08	2.17377E-06
MRPS10	224247_s_at	4.10	1.7014E-07	1.06435E-05
SCAMP1	212417_at	4.09	6.3253E-05	0.001048306
AGTRAP	1555736_a_at	4.08	1.23614E-05	0.00029838
SESN3	235683_at	4.08	0.000103663	0.001524189
TMEM110	213851_at	4.08	3.05979E-09	4.57088E-07
SLC16A1	202236_s_at	4.08	1.18102E-05	0.000288655
HSPH1	206976_s_at	4.08	0.000130817	0.0018181
UBFD1	205687_at	4.08	4.63631E-08	3.91793E-06
NUDT4	206303_s_at	4.07	2.12517E-08	2.11262E-06
DOCK9	215237_at	4.07	2.23978E-07	1.3281E-05
CYP51A1	202314_at	4.07	6.63135E-05	0.001085537
RRAS2	212590_at	4.07	0.000121856	0.001715801
CTSB	213274_s_at	4.07	4.74086E-05	0.000837772
CDV3	228746_s_at	4.06	5.30386E-05	0.000918267
None	243543_at	4.06	5.15342E-05	0.000895624
NUP50	218295_s_at	4.05	4.99362E-08	4.14933E-06
TMEM185A	227880_s_at	4.04	5.04845E-06	0.000148002
DNAJB9	1554462_a_at	4.04	7.90485E-05	0.00124445
RALA	214435_x_at	4.04	8.74711E-08	6.48912E-06
YIPF4	209551_at	4.04	6.12277E-05	0.001020757
LOC729083	1563053_at	4.03	7.52249E-08	5.7847E-06
AMD1	201196_s_at	4.02	5.4403E-06	0.000158108
SORT1	212807_s_at	4.01	1.25621E-08	1.40745E-06

GM2A	212737_at	4.01	2.03364E-05	0.000437753
HIST1H2BG	215779_s_at	4.00	6.17128E-06	0.000174015
LCE1E	1559224_at	4.00	5.08726E-05	0.000886642
HSPA4	211016_x_at	4.00	0.000114367	0.001637515
MAPKAP1	229845_at	4.00	3.25862E-10	7.98867E-08
N4BP1	204601_at	3.99	1.34193E-08	1.47626E-06
CFLAR	211862_x_at	3.99	1.092E-05	0.000271141
PGBD5	219225_at	3.99	1.21283E-07	8.24772E-06
PLXDC2	227276_at	3.99	9.38296E-08	6.77778E-06
LMBRD2	232893_at	3.98	2.39171E-10	6.31723E-08
None	60528_at	3.98	6.54851E-07	3.0733E-05
HIST1H3H	206110_at	3.98	8.5983E-07	3.79429E-05
SEC23B	210293_s_at	3.98	9.61023E-05	0.001441534
RABGGTA	203573_s_at	3.97	1.55575E-09	2.70034E-07
C9orf167	219620_x_at	3.97	1.30707E-09	2.3508E-07
HSPH1	208744_x_at	3.97	9.82585E-07	4.21025E-05
ENAH	1553672_at	3.97	1.24035E-10	3.94279E-08
SEC24A	212902_at	3.96	6.64205E-06	0.000185283
DNAJB9	202843_at	3.96	6.34381E-05	0.0010501
WWC1	213085_s_at	3.96	2.48252E-06	8.60952E-05
LEPROTL1	202595_s_at	3.95	7.16303E-05	0.001152217
CLN8	219340_s_at	3.95	2.55792E-08	2.40971E-06
GALNT6	228303_at	3.95	1.18112E-07	8.08229E-06
GART	217445_s_at	3.94	4.84705E-08	4.05838E-06
CDC73	218578_at	3.94	2.73083E-05	0.000550657
DNAJC3	208499_s_at	3.94	7.15766E-06	0.000196261
C10orf46	227257_s_at	3.93	2.65657E-07	1.52411E-05
IL1F10	224262_at	3.92	1.17967E-12	1.02378E-09
CECR9	1567687_at	3.92	1.0463E-07	7.35305E-06
PGM2	225367_at	3.91	6.10775E-05	0.001018735
MYO5B	225301_s_at	3.91	3.58331E-05	0.000675577
XDH	241994_at	3.91	3.89021E-07	2.04517E-05
VNN3	1553514_a_at	3.91	5.31247E-08	4.3482E-06
ACRV1	206776_x_at	3.89	1.09982E-09	2.05934E-07
KCNK6	223658_at	3.89	0.000102431	0.001511173

SPTY2D1	235440_at	3.89	3.49762E-06	0.000111376
TNFRSF21	218856_at	3.89	1.26254E-07	8.48959E-06
UPK1B	210064_s_at	3.89	1.0131E-06	4.30725E-05
SLAMF7	219159_s_at	3.89	5.61885E-06	0.00016169
None	217682_at	3.88	3.94008E-08	3.44128E-06
FAM40B	1555292_at	3.88	4.14462E-09	5.81044E-07
LASS4	218922_s_at	3.87	2.78782E-05	0.000559559
OASL	210797_s_at	3.87	2.26447E-07	1.33416E-05
GM2A	235678_at	3.87	2.75959E-05	0.00055524
GK	215977_x_at	3.86	1.39749E-08	1.5251E-06
MVK	215649_s_at	3.86	6.03073E-05	0.001008349
C16orf87	226608_at	3.86	7.33258E-05	0.001172516
HIST1H2BI	208523_x_at	3.85	3.31347E-08	2.97967E-06
TNPO1	225766_s_at	3.85	2.46819E-05	0.000508471
PSMD12	202352_s_at	3.85	7.09185E-06	0.00019475
UBE2D1	211764_s_at	3.84	4.48792E-06	0.000135045
TLE3	212769_at	3.84	8.89466E-09	1.0778E-06
TMCC1	213349_at	3.83	6.70652E-10	1.38894E-07
CFLAR	208485_x_at	3.83	1.3548E-05	0.000319145
SDHC	216591_s_at	3.82	4.74876E-05	0.000838582
PPARD	242218_at	3.82	5.95718E-09	7.82954E-07
HSPA4	211015_s_at	3.82	8.99452E-05	0.001368704
STX19	1555173_at	3.81	3.44034E-05	0.00065499
CITED2	209357_at	3.81	0.000143395	0.001951369
KIF1B	225878_at	3.81	1.16875E-06	4.81185E-05
UBE2K	202347_s_at	3.81	7.24341E-08	5.63791E-06
GSTP1	200824_at	3.81	2.39188E-06	8.39385E-05
JMJD1C	241661_at	3.81	3.96743E-05	0.000731438
AFF4	219199_at	3.80	4.69097E-08	3.95801E-06
GRHL1	1552685_a_at	3.80	9.06124E-05	0.001375793
SLC16A1	1557918_s_at	3.79	2.03807E-06	7.39915E-05
F11R	222354_at	3.79	1.3823E-05	0.000323948
MPZL1	210087_s_at	3.78	5.83818E-06	0.000166773
EPS15L1	231926_at	3.77	8.08545E-06	0.00021533
POLB	203616_at	3.76	1.48211E-09	2.59725E-07

EPRS	200842_s_at	3.76	4.28571E-06	0.000129962
MFAP3L	210492_at	3.75	1.39326E-09	2.48376E-07
NFE2	209930_s_at	3.75	0.000110647	0.001598317
LOC401074	1559827_at	3.75	0.000122606	0.001723262
PPARD	37152_at	3.75	5.00228E-07	2.48185E-05
YKT6	217785_s_at	3.75	3.90187E-06	0.000121697
CTH	206085_s_at	3.74	1.54032E-10	4.63223E-08
SPNS2	225671_at	3.74	8.0128E-08	6.07628E-06
SLC27A4	225779_at	3.74	1.14851E-05	0.000282097
INO80D	231152_at	3.74	0.000140032	0.001912627
None	240237_at	3.74	3.54451E-07	1.89993E-05
PGM3	210041_s_at	3.74	1.55889E-06	5.99806E-05
RHBDL2	1554895_a_at	3.73	3.47958E-06	0.000110931
ZNF589	210062_s_at	3.73	1.08486E-09	2.03831E-07
TANK	210458_s_at	3.73	8.78353E-05	0.001344455
FAM83A	242944_at	3.73	4.10882E-06	0.000125995
CLN8	223912_s_at	3.73	1.43567E-05	0.000333739
CHM	207099_s_at	3.73	2.70716E-07	1.54507E-05
AZI2	227905_s_at	3.72	0.000129705	0.001805858
None	228158_at	3.72	1.13104E-08	1.30463E-06
TXNL4B	222748_s_at	3.72	9.40525E-09	1.12185E-06
SEPT8	209000_s_at	3.72	2.63107E-05	0.000536167
CCDC85C	222809_x_at	3.71	1.84437E-06	6.83205E-05
PHF23	1555789_s_at	3.71	2.87024E-10	7.33321E-08
MED8	213696_s_at	3.71	2.31908E-08	2.23626E-06
TMEM40	219503_s_at	3.71	0.000144517	0.001960432
MCL1	214056_at	3.70	8.04372E-05	0.001261952
SERINC2	224762_at	3.70	8.10887E-08	6.11521E-06
None	231231_at	3.70	5.76763E-08	4.64426E-06
MPZL2	230518_at	3.69	0.000116687	0.001660992
None	217553_at	3.69	3.14823E-06	0.000102825
CRIPT	227942_s_at	3.69	0.000121516	0.001713663
SSR1	200889_s_at	3.69	6.00364E-05	0.001004742
GSPT1	201912_s_at	3.69	2.60284E-06	8.93909E-05
TM7SF2	210130_s_at	3.68	5.62759E-05	0.000957637

STARD4	226390_at	3.68	2.10936E-07	1.26875E-05
None	1556773_at	3.68	1.67436E-06	6.3222E-05
SIPA1L2	233587_s_at	3.67	9.5279E-07	4.13115E-05
SGMS2	242963_at	3.67	9.27861E-08	6.7551E-06
PAFAH1B1	200815_s_at	3.66	1.79182E-08	1.85194E-06
FRMD8	227964_at	3.66	9.32787E-07	4.06376E-05
ENSA	221487_s_at	3.66	1.05759E-05	0.000264156
ACOT11	214763_at	3.66	2.21628E-06	7.89415E-05
STAM2	215044_s_at	3.66	3.64938E-07	1.94663E-05
ABHD12	228124_at	3.66	5.95215E-06	0.000168984
PCSK6	207414_s_at	3.65	1.50952E-06	5.83272E-05
PTS	209694_at	3.65	1.00421E-05	0.000254073
None	238973_s_at	3.65	1.76366E-08	1.83324E-06
CARD6	224414_s_at	3.65	4.83736E-06	0.000142964
C6orf141	1552575_a_at	3.65	1.79548E-09	3.02056E-07
TMBIM6	200803_s_at	3.65	3.78266E-06	0.000118929
CCNE1	213523_at	3.64	7.28636E-07	3.35157E-05
STRBP	233252_s_at	3.64	2.09731E-06	7.56401E-05
C1orf104	230256_at	3.64	3.64437E-05	0.000684968
BTG2	201235_s_at	3.63	2.39248E-05	0.000496616
DLX5	213707_s_at	3.63	1.61842E-05	0.000365951
FAM169A	235048_at	3.63	1.08014E-05	0.000268683
SLC7A8	216092_s_at	3.62	9.94569E-09	1.17194E-06
RFTN1	1569787_at	3.62	5.55616E-07	2.70751E-05
KLHDC10	209254_at	3.62	5.15229E-05	0.000895624
MBD1	1555611_s_at	3.62	2.83561E-05	0.000566548
GLRX3	209080_x_at	3.62	2.35244E-05	0.000490354
HN1	217755_at	3.61	8.82424E-06	0.000230734
CSNK2A1	212075_s_at	3.60	5.24302E-07	2.58022E-05
ACRV1	207990_x_at	3.60	1.95352E-08	1.98899E-06
None	224239_at	3.60	5.5672E-07	2.71048E-05
BSPRY	222746_s_at	3.59	5.09387E-05	0.000887248
ERP27	227450_at	3.59	2.41044E-06	8.45353E-05
ARL4A	205020_s_at	3.59	3.89675E-05	0.00072256
BCL2L1	206665_s_at	3.59	1.44757E-05	0.000335648

ZNF561	230205_at	3.58	1.57802E-05	0.000359044
None	231894_at	3.58	4.07513E-05	0.00074678
MELK	204825_at	3.58	3.92352E-09	5.5719E-07
BAMBI	203304_at	3.58	1.53159E-05	0.000350815
TCP11L2	1565525_a_at	3.58	1.27606E-07	8.55006E-06
NCBP1	209520_s_at	3.58	0.000102891	0.001515915
HIST1H2BE	208527_x_at	3.58	1.6059E-09	2.7698E-07
None	239669_at	3.57	1.6396E-07	1.03482E-05
MARCH5	218582_at	3.57	2.48326E-06	8.60952E-05
FAM63A	221856_s_at	3.57	2.88594E-11	1.29499E-08
NPM1	221923_s_at	3.57	1.03763E-06	4.38425E-05
UCHL3	204616_at	3.57	0.000113058	0.001625286
DLX1	242138_at	3.57	2.10209E-06	7.57127E-05
SCRN3	1555595_at	3.57	3.42822E-09	5.03865E-07
PSME3	200987_x_at	3.56	1.56751E-05	0.000357099
GTPBP4	218239_s_at	3.56	4.93428E-07	2.46174E-05
ARF3	200734_s_at	3.55	1.10729E-08	1.28265E-06
ALOXE3	207708_at	3.55	8.75875E-05	0.00134179
DDX10	204977_at	3.55	1.04962E-09	1.98574E-07
GNB5	207124_s_at	3.55	5.57117E-06	0.000160487
CCDC88C	215343_at	3.55	3.32035E-08	2.98095E-06
STAMBP	202811_at	3.55	1.83117E-06	6.79696E-05
None	226706_at	3.55	7.46789E-08	5.7508E-06
TPM4	1567107_s_at	3.54	9.82523E-08	7.00384E-06
HSPB8	221667_s_at	3.54	6.74964E-06	0.000187518
None	1562979_at	3.54	3.61114E-07	1.92837E-05
SF3B3	200687_s_at	3.54	2.23888E-07	1.3281E-05
KPNA6	212102_s_at	3.53	1.13148E-06	4.68663E-05
IL8	202859_x_at	3.53	1.58032E-05	0.000359248
SERPINE1	202628_s_at	3.52	3.19353E-05	0.00061939
C9orf167	233589_x_at	3.51	1.81774E-09	3.04863E-07
None	221079_s_at	3.51	8.20535E-05	0.001278506
FAM199X	227133_at	3.51	0.000111363	0.001605691
TMEM165	218095_s_at	3.51	4.5498E-06	0.000136396
CXCL17	226960_at	3.51	0.000123215	0.001731373

KIAA1199	212942_s_at	3.51	7.47408E-06	0.0002024
CPEB4	224829_at	3.51	0.000135809	0.001870372
SEC23B	201583_s_at	3.50	1.35797E-07	8.94542E-06
SSR3	237817_at	3.50	4.03881E-11	1.64792E-08
AMACR	209424_s_at	3.49	3.78409E-08	3.33165E-06
SOS1	212777_at	3.49	7.29821E-07	3.35157E-05
MACC1	1566764_at	3.49	8.60699E-06	0.000226462
CHD2	1554014_at	3.49	5.11696E-06	0.00014985
SLC39A9	217859_s_at	3.48	4.20561E-05	0.000765198
KCNG1	214595_at	3.48	1.29755E-05	0.000308852
STXBP6	226462_at	3.48	7.20623E-08	5.62858E-06
RC3H2	220201_at	3.48	7.7972E-08	5.96264E-06
LZIC	226087_at	3.48	4.21298E-06	0.000128429
CASP4	209310_s_at	3.47	2.67219E-05	0.000542727
OPN3	219032_x_at	3.47	5.51263E-06	0.000159433
DENND2D	221081_s_at	3.47	2.9409E-06	9.78065E-05
TOLLIP	217930_s_at	3.47	1.1473E-05	0.000281926
CLCN5	206704_at	3.46	1.39228E-09	2.48376E-07
NADSYN1	218840_s_at	3.46	1.65009E-06	6.26804E-05
ARG1	231663_s_at	3.46	9.58933E-07	4.14777E-05
CARS	240983_s_at	3.46	4.70656E-06	0.000139626
SAR1A	201543_s_at	3.45	3.12641E-06	0.000102235
CA5BP	238435_at	3.45	4.09228E-06	0.00012577
ACE2	219962_at	3.45	2.43882E-06	8.51483E-05
UBE2J1	222435_s_at	3.44	1.40254E-05	0.000327429
LNPEP	207904_s_at	3.44	2.47741E-07	1.43488E-05
ITCH	217094_s_at	3.43	1.38724E-06	5.50019E-05
None	236069_at	3.43	1.58719E-05	0.00036053
FBLIM1	1554795_a_at	3.43	1.99279E-09	3.24273E-07
CDS1	226187_at	3.42	5.72553E-06	0.000164241
CLPX	204809_at	3.42	7.20188E-05	0.001156765
GAB1	225998_at	3.42	7.16481E-07	3.30667E-05
PSME3	209853_s_at	3.42	2.907E-05	0.000577119
GLRX3	214205_x_at	3.41	5.0807E-09	6.94469E-07
TOMM5	225036_at	3.41	3.23269E-05	0.000624436

DEFB124	1568377_x_at	3.41	2.27092E-05	0.000476541
KIAA0146	228325_at	3.41	8.23599E-07	3.66696E-05
SLC16A1	202235_at	3.41	1.55982E-05	0.000355644
BNIP3L	221479_s_at	3.41	8.13045E-06	0.000216318
RBM33	1554095_at	3.40	3.44466E-09	5.04787E-07
HIST1H2BC	236193_at	3.40	2.72334E-09	4.14759E-07
TNFSF9	206907_at	3.40	1.69741E-07	1.06307E-05
DHRS7	210788_s_at	3.40	3.23372E-05	0.000624436
TRY6	215395_x_at	3.40	1.24129E-08	1.39645E-06
C17orf42	219146_at	3.40	9.73964E-06	0.000247566
SURF4	222979_s_at	3.40	0.000112294	0.001616976
MALT1	208309_s_at	3.38	0.000138096	0.001889521
HIST1H3E	236278_at	3.38	6.16955E-09	8.03336E-07
PHLDA1	217997_at	3.38	4.36158E-05	0.000787547
DCUN1D5	223151_at	3.38	3.77493E-08	3.32894E-06
SIPA1L2	225056_at	3.37	8.68249E-08	6.45871E-06
UBE2G1	209141_at	3.37	3.9272E-06	0.000122069
PRDM1	228964_at	3.37	1.5219E-05	0.000349328
PLA2G12A	221027_s_at	3.37	6.84761E-05	0.001113252
ANO10	218910_at	3.37	2.69745E-09	4.13117E-07
NIPAL4	230188_at	3.37	6.05625E-05	0.001011379
ROD1	224618_at	3.36	0.000136271	0.001875787
LOC728431	229678_at	3.36	2.69225E-05	0.000544803
PLA2G4D	1554914_at	3.36	3.95209E-12	2.77027E-09
HTR7	236281_x_at	3.36	9.70037E-07	4.17612E-05
USO1	201831_s_at	3.36	1.68038E-05	0.00037731
ACRV1	207991_x_at	3.36	2.53749E-10	6.63814E-08
ACOX1	213501_at	3.36	0.000133149	0.001843012
CCRN4L	1554283_at	3.35	7.09616E-09	8.91914E-07
TMEM170A	228505_s_at	3.35	2.19372E-07	1.31084E-05
HIGD1A	217845_x_at	3.35	5.70083E-07	2.77307E-05
COCH	1554241_at	3.35	5.28876E-05	0.000916233
FAM160A1	242687_at	3.34	1.33158E-07	8.82475E-06
YARS	212048_s_at	3.34	2.91211E-07	1.63135E-05
KYNU	210663_s_at	3.34	5.1228E-07	2.53474E-05

TMEM151B	1564066_at	3.34	1.9584E-05	0.00042516
HIC2	212964_at	3.33	4.52121E-07	2.28463E-05
CSNK2A1	206075_s_at	3.33	4.12692E-05	0.000755182
FBXO10	1558062_at	3.33	9.67406E-08	6.94133E-06
MAPKAPK3	202787_s_at	3.32	1.63244E-06	6.22412E-05
CYP27B1	205676_at	3.32	1.64109E-06	6.24403E-05
PTGR1	242775_at	3.32	6.33377E-05	0.001048755
SMOX	1555680_a_at	3.32	2.37931E-08	2.2662E-06
NIT1	202891_at	3.32	1.22844E-10	3.94279E-08
WDR77	201420_s_at	3.31	7.33132E-11	2.74548E-08
CDKN2A	207039_at	3.31	3.86535E-09	5.53242E-07
ACRC	238825_at	3.31	2.8313E-06	9.49703E-05
ATP2C1	209934_s_at	3.30	6.92889E-05	0.00112248
None	236388_at	3.30	2.54445E-06	8.77445E-05
GTPBP2	223789_s_at	3.30	6.53249E-05	0.001071598
PSMB2	201404_x_at	3.30	2.03089E-05	0.000437649
PGLYRP3	1553059_at	3.30	8.02566E-05	0.001260203
ESRP2	219395_at	3.30	1.99865E-05	0.000432605
PFKFB2	209992_at	3.30	5.88489E-08	4.73171E-06
EXOSC3	227916_x_at	3.29	2.74111E-05	0.000552211
CNDP2	217752_s_at	3.29	5.546E-06	0.00015993
C20orf24	224376_s_at	3.29	1.86728E-09	3.08439E-07
OSTM1	243287_s_at	3.29	1.98461E-08	2.0057E-06
PGGT1B	206288_at	3.28	3.07031E-06	0.000100895
FBXO10	227222_at	3.28	4.4275E-07	2.25815E-05
OVOL1	206604_at	3.28	7.79674E-06	0.000209169
LTB4R	236172_at	3.28	2.79064E-05	0.000559715
None	213189_at	3.28	4.57367E-06	0.000136722
HTATIP2	207180_s_at	3.27	2.80508E-06	9.42833E-05
FKBP15	76897_s_at	3.27	1.18462E-06	4.85525E-05
LOC643008	229740_at	3.27	5.53648E-07	2.70033E-05
CKAP4	200998_s_at	3.27	2.87247E-05	0.00057214
IFRD1	202147_s_at	3.27	1.29431E-05	0.000308807
FGFR2	211399_at	3.27	2.18232E-06	7.79858E-05
NMNAT1	223692_at	3.27	3.05523E-05	0.000600448

CFLAR	209508_x_at	3.27	0.000139093	0.001901081
MAPK14	211561_x_at	3.26	4.09529E-06	0.000125792
TM9SF4	212194_s_at	3.26	8.81133E-05	0.001346449
DDA1	218260_at	3.26	2.67743E-05	0.000543589
ETFDH	205530_at	3.26	2.71553E-06	9.22906E-05
HSPA4	208815_x_at	3.26	7.39838E-07	3.37487E-05
CTTN	214073_at	3.25	6.4869E-07	3.06015E-05
C1orf31	225638_at	3.25	7.26974E-08	5.63791E-06
SLMO2	229835_s_at	3.25	2.06601E-07	1.25093E-05
CXCL1	204470_at	3.24	1.21629E-07	8.26096E-06
STK4	205411_at	3.24	8.38992E-08	6.28382E-06
HECTD1	1557100_s_at	3.24	2.15475E-05	0.000458764
CRIPT	222702_x_at	3.24	6.97667E-05	0.001127506
FBXO22	225737_s_at	3.24	3.01548E-06	9.96202E-05
PYCARD	221666_s_at	3.23	5.44936E-05	0.00093693
HOOK3	235114_x_at	3.23	0.000128359	0.00179077
UBE3C	1554794_a_at	3.23	4.90439E-06	0.000144481
STYXL1	218321_x_at	3.23	2.88419E-07	1.61759E-05
PTHLH	206300_s_at	3.23	2.24566E-07	1.32881E-05
USP32	211702_s_at	3.22	7.53643E-09	9.36487E-07
PDE12	1554915_a_at	3.22	1.07448E-05	0.00026752
KLHL18	212882_at	3.22	2.24932E-07	1.32953E-05
ACRV1	207969_x_at	3.21	6.18183E-08	4.90321E-06
TUSC3	209228_x_at	3.21	7.16985E-06	0.0001963
ALAS1	205633_s_at	3.21	1.3941E-05	0.000325875
MALT1	210018_x_at	3.21	8.99236E-05	0.001368704
AMACR	209426_s_at	3.21	4.6243E-07	2.33026E-05
ACOX1	209600_s_at	3.20	1.4925E-05	0.000344169
BTBD10	223174_at	3.20	1.1958E-06	4.88279E-05
TRIB3	218145_at	3.19	4.1569E-07	2.14277E-05
MYCBP	203360_s_at	3.19	2.72224E-05	0.00054922
PTPN22	208010_s_at	3.18	1.19711E-09	2.20756E-07
DNAJC5	224613_s_at	3.18	1.47685E-06	5.75529E-05
ARID5B	241969_at	3.18	1.16749E-09	2.17859E-07
TUBG1	201714_at	3.18	2.0369E-06	7.39915E-05

RRAS2	208456_s_at	3.18	1.04924E-05	0.00026267
UBB	217144_at	3.17	3.03673E-05	0.000597456
PLA2G3	220780_at	3.17	4.31629E-05	0.000781434
ATP6V0A1	205095_s_at	3.17	1.47969E-06	5.76202E-05
ATP13A4	1559571_a_at	3.16	1.02426E-08	1.19661E-06
BCL2L1	215037_s_at	3.16	7.51916E-05	0.001196479
C9orf40	222781_s_at	3.16	3.26364E-09	4.82269E-07
TMPRSS13	223659_at	3.15	7.87686E-05	0.001242907
C1orf55	1553338_at	3.15	2.73626E-06	9.27206E-05
NSDHL	209279_s_at	3.15	1.03027E-05	0.000258869
PLCXD1	218951_s_at	3.15	0.000133882	0.001850353
EIF2S1	201144_s_at	3.15	3.93529E-08	3.44128E-06
None	234120_at	3.15	2.36378E-08	2.25828E-06
KPNA2	211762_s_at	3.15	4.524E-08	3.8494E-06
PLAU	211668_s_at	3.15	2.88575E-06	9.66187E-05
ASPHD2	227015_at	3.14	2.05356E-09	3.33171E-07
ACOT7	208002_s_at	3.14	2.0084E-05	0.000434545
UBE2D3	200669_s_at	3.14	2.5704E-05	0.000526551
SEPSECS	1553167_a_at	3.14	1.02655E-05	0.000258292
WDR77	201421_s_at	3.14	2.16876E-07	1.29876E-05
KIAA0513	204546_at	3.14	1.48E-07	9.48638E-06
PRSS2	205402_x_at	3.14	1.76549E-06	6.59795E-05
TPM4	212481_s_at	3.14	9.78392E-07	4.20547E-05
P2RY1	207455_at	3.13	1.02823E-07	7.2634E-06
ACACA	214358_at	3.13	3.59186E-08	3.18807E-06
KPNA1	202058_s_at	3.13	4.69638E-07	2.35789E-05
FAM43A	1558174_at	3.13	9.29175E-07	4.05269E-05
ZFP42	243161_x_at	3.13	1.30157E-10	4.08985E-08
KIAA0494	201777_s_at	3.12	1.59556E-05	0.00036213
ACE2	222257_s_at	3.12	7.3396E-09	9.16193E-07
SGPL1	208381_s_at	3.12	2.27067E-07	1.33483E-05
HERC4	208055_s_at	3.12	0.000115397	0.001648632
None	214236_at	3.12	4.02882E-07	2.09388E-05
EIF4E2	213571_s_at	3.12	2.23108E-07	1.32778E-05
SLCO4A1	219911_s_at	3.12	2.68694E-05	0.000544509

DCUN1D1	222678_s_at	3.12	9.35613E-06	0.00024005
VTA1	224437_s_at	3.12	6.87033E-05	0.001114975
None	1559667_at	3.12	4.36122E-05	0.000787547
None	235193_at	3.11	0.000115786	0.001652037
GLRX3	216532_x_at	3.11	7.45042E-08	5.74608E-06
AFF4	243487_at	3.11	8.82214E-06	0.000230734
TMEM185A	1554105_at	3.10	7.51324E-06	0.000203058
KIAA0513	1554440_at	3.10	1.22005E-06	4.97437E-05
WASL	205810_s_at	3.10	1.76711E-06	6.59949E-05
GSPT1	215438_x_at	3.10	9.72148E-08	6.96621E-06
HMOX2	218120_s_at	3.09	1.63472E-08	1.7355E-06
TAC4	1553094_at	3.09	4.85806E-05	0.00085489
SPAG9	206748_s_at	3.09	5.89148E-06	0.000167701
C12orf56	236840_at	3.09	4.42426E-07	2.25815E-05
RER1	202297_s_at	3.09	1.19502E-07	8.14685E-06
MAP7	215471_s_at	3.09	1.66104E-05	0.000373733
EPB41L5	229292_at	3.09	3.97455E-05	0.000732172
SNAP29	239084_at	3.09	4.97031E-05	0.00087072
LLGL2	1554006_a_at	3.08	2.09441E-06	7.55853E-05
LOC727916	1559322_at	3.08	1.8707E-07	1.15833E-05
CPSF2	233208_x_at	3.08	3.08406E-06	0.000101274
HOMER3	215489_x_at	3.08	1.44423E-06	5.66857E-05
SLC7A8	217248_s_at	3.08	1.76428E-06	6.59795E-05
TXK	206828_at	3.08	2.99734E-06	9.92196E-05
TUBB2A	204141_at	3.08	2.7294E-06	9.25744E-05
NCRNA00189	1553608_a_at	3.08	6.01284E-09	7.88374E-07
TUBB3	213476_x_at	3.07	1.37689E-05	0.000322957
MARVELD2	235955_at	3.07	1.42677E-07	9.29781E-06
SERPINE1	202627_s_at	3.07	0.000128753	0.001795808
BNC1	206581_at	3.07	9.92138E-05	0.001474053
SPAG17	233516_s_at	3.07	3.91473E-06	0.00012182
