# Supplementary material for: Heterogeneity of Inflammatory and Cytokine Networks in Chronic Plaque Psoriasis
Source: PLoS One. 2012 Mar 29;7(3):e34594. doi: 10.1371/journal.pone.0034594 (PMC3315545; doi:10.1371/journal.pone.0034594)
Supplement: Table S4 — Set of 1000 signature transcripts with high expression in (blood-derived) CD4+ T-cells relative to normal skin. Heatmaps shown in Figures 1, S1 and S5 are based upon the expression patterns of cell type-specific “signature transcripts” in lesional (PP) and non-lesional (PN) skin samples. This table provides an example of the 1000 signature transcripts associated with one cell type (blood-derived CD4+ T-cells). (PDF) [file pone.0034594.s019.pdf]

Table S4. Set of 1000 signature transcripts with high expression in CD4+ T-cells isolated from blood. Heatmaps shown in Figures 1, S1 and S5 are based upon expression patterns of cell type-specific signature transcripts in lesional (PP) and non-lesional (PN) skin samples. This table lists the 1000 signature transcripts associated with CD4+ T-cells isolated from peripheral blood. Signature transcripts were identified based upon a two-sample comparison between RNA extracted from CD4+ T-cells (*n* = 2) and RNA extracted from whole skin (*n* = 21). The two CD4+ T-cell expression profiles were obtained from GEO series GSE14596 (samples GSM364917 and GSM364918). The 21 whole skin expression profiles were obtained from GEO series GSE7307, GSE6281, GSE16161 and GSE17539. Transcripts were identified using the following three steps. First, transcripts with higher expression on average in CD4+ T-cells were identified. Second, the top 2000 of these transcripts with lowest p-values were isolated. Third, the top 1000 of these transcripts with largest fold-change were selected as signature transcripts (i.e., CD4+ T-cell expression / whole skin expression). These 1000 transcripts are listed in the table below and are sorted according to the estimated fold-change ratio. The fourth column lists the raw p-values obtained from the test of differential expression between CD4+ T-cells and whole skin. The fifth column lists Benjamini-Hochberg-adjusted p-values.

Gene Symbol	Probe Set ID	Fold-Change (CD4+ T-cells / Whole Skin)	P-value	Adjusted P-Value
IL2	207849_at	601.77	9.74978E-28	5.33069E-23
MIR155HG	229437_at	429.90	1.44724E-19	6.4472E-16
IFNG	210354_at	306.71	1.00271E-17	1.95796E-14
GZMB	210164_at	177.94	1.48898E-10	1.40604E-08
CCL4	204103_at	165.77	2.24516E-13	7.35055E-11
LTA	206975_at	145.60	1.10629E-15	1.0252E-12
None	205114_s_at	143.53	4.78293E-14	2.16121E-11
CLEC2D	220132_s_at	141.43	1.89998E-21	3.0157E-17
TNFRSF9	207536_s_at	139.89	1.00181E-17	1.95796E-14
CXCL13	205242_at	124.66	2.70532E-24	7.39568E-20
ZBED2	219836_at	121.36	9.87376E-16	9.47102E-13
None	214567_s_at	116.83	6.23874E-12	1.09328E-09
None	236198_at	112.78	1.53294E-19	6.4472E-16
BCL2A1	205681_at	91.53	1.10175E-13	4.06472E-11
IL2RA	211269_s_at	89.83	1.36413E-18	3.72918E-15
CD28	206545_at	88.57	1.4018E-17	2.64287E-14
CCL20	205476_at	85.46	7.29109E-14	2.97493E-11
XCL1	206366_x_at	82.13	1.22962E-11	1.88891E-09
IL3	207906_at	81.81	4.74248E-15	3.20118E-12
ITK	211339_s_at	79.88	1.29689E-10	1.255E-08
GPR171	207651_at	78.13	6.17956E-11	6.79813E-09

IL2RA	206341_at	75.42	1.83943E-16	2.51428E-13
None	236787_at	70.69	3.9228E-16	4.53882E-13
CRTAM	206914_at	67.65	7.62281E-20	4.16777E-16
CLEC2D	233500_x_at	66.11	7.55466E-17	1.18015E-13
CTLA4	236341_at	62.56	3.161E-18	7.51424E-15
TNF	207113_s_at	61.68	1.0979E-19	5.45708E-16
ICOS	210439_at	61.64	1.13783E-14	6.91231E-12
SAMSN1	220330_s_at	59.37	7.07738E-08	2.16176E-06
CD28	211861_x_at	57.72	2.20627E-21	3.0157E-17
SH2D1A	210116_at	57.26	1.64651E-13	5.69764E-11
None	236099_at	55.80	6.33038E-14	2.652E-11
CD69	209795_at	55.44	3.81995E-08	1.29403E-06
CD2	205831_at	53.52	3.87788E-12	7.43941E-10
TNFSF14	207907_at	49.39	2.58113E-12	5.30538E-10
CCND2	200951_s_at	48.70	5.2132E-14	2.29864E-11
None	211796_s_at	48.58	3.15502E-08	1.10224E-06
AIM2	206513_at	44.67	1.44236E-11	2.13138E-09
PTPN22	206060_s_at	43.12	8.03027E-15	5.04661E-12
SAMSN1	1555638_a_at	42.07	5.83382E-15	3.79719E-12
SLAMF1	206181_at	41.86	1.87154E-18	4.87269E-15
CHAC2	235117_at	41.41	1.05117E-12	2.49889E-10
NAMPT	1555167_s_at	40.90	1.06724E-10	1.0687E-08
C6orf142	224533_s_at	40.39	6.51872E-19	2.09653E-15
TNFRSF4	214228_x_at	40.17	4.54123E-15	3.10365E-12
CHEK1	205394_at	39.68	1.6605E-17	2.92863E-14
None	216191_s_at	39.49	7.2745E-09	3.30618E-07
CD200	209582_s_at	39.38	5.15766E-19	1.76247E-15
None	210972_x_at	38.75	1.3789E-10	1.32034E-08
CHEK1	205393_s_at	38.55	1.03147E-18	2.96818E-15
None	211902_x_at	37.52	1.83483E-09	1.07523E-07
None	210915_x_at	37.23	2.99343E-09	1.60015E-07
NKG7	213915_at	37.22	3.82893E-10	3.03401E-08
LCK	204891_s_at	36.93	6.82595E-09	3.14679E-07
TIPIN	219258_at	36.89	2.06433E-12	4.42617E-10
RAC2	207419_s_at	36.84	5.28469E-08	1.70668E-06

TNFRSF9	211786_at	36.63	2.86064E-19	1.0427E-15
None	213193_x_at	36.30	4.42116E-09	2.20957E-07
TRAT1	217147_s_at	36.00	2.57234E-13	8.26266E-11
DHX9	212105_s_at	35.66	1.36137E-09	8.47752E-08
None	221648_s_at	34.95	9.19538E-16	9.14104E-13
CCR7	206337_at	34.65	5.52821E-11	6.19231E-09
LCK	204890_s_at	34.52	7.94557E-10	5.45759E-08
CENPV	226611_s_at	34.45	2.22427E-15	1.71284E-12
None	217394_at	34.42	2.10405E-16	2.80582E-13
NR4A3	207978_s_at	34.31	5.89123E-16	6.19429E-13
CD3D	213539_at	34.10	3.74417E-10	2.9798E-08
ETS1	1555355_a_at	33.71	6.62336E-10	4.71527E-08
None	1559263_s_at	33.65	7.68186E-09	3.46254E-07
GLS	223079_s_at	33.55	5.65743E-08	1.80783E-06
CYTIP	209606_at	32.86	1.39621E-07	3.83414E-06
CD226	207315_at	32.68	7.33624E-21	6.83853E-17
STIP1	212009_s_at	32.66	3.83894E-09	1.97083E-07
PTPN22	236539_at	32.17	3.23797E-14	1.62418E-11
TMPO	209754_s_at	32.04	3.27566E-08	1.13857E-06
CTLA4	234362_s_at	31.68	2.28287E-12	4.8006E-10
INPP4B	205376_at	30.69	3.18376E-12	6.30696E-10
LTB	207339_s_at	30.31	1.29136E-08	5.25336E-07
F5	204714_s_at	30.23	4.70519E-13	1.30587E-10
RP6-213H19.1	224407_s_at	30.02	7.37055E-12	1.2554E-09
None	209671_x_at	29.54	6.8411E-10	4.83252E-08
CAMK4	241871_at	28.99	9.53365E-14	3.72323E-11
PTPN22	208010_s_at	28.90	5.40726E-18	1.20573E-14
None	242388_x_at	28.69	6.2E-11	6.80693E-09
CCND2	200952_s_at	28.20	1.10771E-13	4.06472E-11
KLHL6	228167_at	28.17	7.93049E-13	2.01674E-10
None	235735_at	28.14	4.21926E-12	7.84346E-10
NAA15	222837_s_at	27.22	2.04576E-11	2.76861E-09
CD48	204118_at	26.55	2.91486E-08	1.03152E-06
RAB8B	219210_s_at	26.55	2.07413E-11	2.79317E-09
CSF2	210229_s_at	26.31	6.50948E-16	6.59085E-13

CD27	206150_at	26.11	6.79022E-14	2.81254E-11
TRA@	234849_at	25.89	5.42464E-14	2.37274E-11
TRA@	217412_at	25.61	6.40631E-13	1.70032E-10
TFRC	237215_s_at	25.53	3.33267E-11	4.13183E-09
DUSP2	204794_at	25.43	1.74141E-08	6.70976E-07
EXOSC3	223490_s_at	25.36	8.09425E-09	3.61221E-07
CDK6	235287_at	25.27	2.15459E-11	2.87323E-09
CORO1A	209083_at	25.24	3.03777E-08	1.07086E-06
FASLG	211333_s_at	25.03	2.6343E-12	5.39439E-10
SLAMF1	1555626_a_at	24.89	4.03706E-14	1.90787E-11
TRD@	234013_at	24.75	1.94326E-12	4.23298E-10
DUSP4	204014_at	24.50	1.18589E-07	3.3422E-06
NR4A3	209959_at	24.38	1.63693E-13	5.69764E-11
GART	217445_s_at	24.29	2.68321E-15	1.98249E-12
IRF4	204562_at	23.99	3.72828E-16	4.43139E-13
MGC40069	243602_at	23.95	1.96976E-12	4.25678E-10
DUSP4	204015_s_at	23.81	8.47503E-11	8.80935E-09
IL21R	221658_s_at	23.31	2.80473E-12	5.67959E-10
IL12RB2	206999_at	22.94	8.8014E-15	5.46837E-12
KBTBD8	239835_at	22.85	1.08077E-16	1.64142E-13
CCDC58	235244_at	22.80	3.0875E-13	9.75774E-11
LRRN3	209840_s_at	22.77	1.72429E-09	1.02474E-07
None	214995_s_at	22.74	1.37216E-12	3.10011E-10
MDFIC	217599_s_at	22.52	4.22894E-09	2.133E-07
CD200	209583_s_at	22.44	5.1911E-10	3.90125E-08
IL2RG	204116_at	22.23	1.26747E-08	5.19092E-07
CKS2	204170_s_at	22.21	2.62125E-08	9.48488E-07
ANP32A	201043_s_at	22.02	6.8753E-08	2.11184E-06
SH2D2A	207351_s_at	21.71	8.88568E-14	3.52047E-11
LYAR	223413_s_at	21.65	2.50025E-12	5.19777E-10
LRRN3	209841_s_at	21.63	5.24641E-10	3.90269E-08
RAB27A	210951_x_at	21.58	2.22331E-11	2.95313E-09
None	217143_s_at	21.53	2.22112E-07	5.61702E-06
LEF1	221558_s_at	21.47	5.25719E-11	6.00077E-09
ASNS	205047_s_at	21.41	2.62618E-11	3.4339E-09

C4orf46	235088_at	21.18	1.90854E-11	2.64176E-09
TRAC	209670_at	21.01	3.09735E-11	3.89305E-09
CDC42SE2	1552613_s_at	20.98	1.38763E-07	3.81632E-06
KIAA0748	222920_s_at	20.76	7.80036E-09	3.51016E-07
FASLG	210865_at	20.58	6.85612E-13	1.7682E-10
STAT1	AFFX-HUMISGF3A/M97935_5_at	20.57	1.77575E-11	2.4831E-09
LARP4	214155_s_at	20.57	4.29455E-08	1.43524E-06
CENPV	226610_at	20.37	2.76123E-11	3.56904E-09
FCF1	219927_at	20.21	5.57286E-13	1.5084E-10
MTHFD1L	225520_at	20.16	7.92921E-09	3.54481E-07
CTLA4	221331_x_at	20.10	3.38415E-15	2.40297E-12
CD3G	206804_at	20.00	6.69415E-09	3.09385E-07
SHMT2	214437_s_at	19.97	3.82564E-09	1.96585E-07
RAB27A	209514_s_at	19.96	4.99863E-12	9.07973E-10
RAB39B	230075_at	19.82	7.50456E-21	6.83853E-17
SFXN1	230069_at	19.77	6.51892E-07	1.37668E-05
POU2AF1	205267_at	19.59	1.07635E-08	4.54786E-07
TRAF1	205599_at	19.35	2.90075E-16	3.57284E-13
DTL	222680_s_at	19.30	1.06572E-15	1.00462E-12
PTPN7	204852_s_at	19.18	1.65744E-14	9.06204E-12
GABPB1	206173_x_at	19.15	4.54025E-14	2.07455E-11
CD247	210031_at	19.14	1.61945E-08	6.3065E-07
None	238443_at	19.07	1.73115E-11	2.45209E-09
BCL2L11	1558143_a_at	19.01	1.02088E-10	1.02811E-08
LOC439949	232001_at	18.80	1.93191E-10	1.75752E-08
CD3E	205456_at	18.72	8.02452E-11	8.43732E-09
CENPN	219555_s_at	18.68	6.92214E-15	4.45256E-12
RAB39B	238695_s_at	18.63	1.34151E-20	1.04781E-16
IL22	222974_at	18.57	2.14794E-19	8.38846E-16
EPRS	200841_s_at	18.55	5.92227E-08	1.87384E-06
C1orf228	242520_s_at	18.52	1.54828E-15	1.36536E-12
DDX3Y	205001_s_at	18.47	1.04596E-07	3.0004E-06
TAF1A	206613_s_at	18.46	1.58969E-14	8.77944E-12
CDC6	203967_at	18.39	7.47369E-17	1.18015E-13
CTLA4	231794_at	18.38	2.7317E-12	5.57297E-10

TRD@	215796_at	18.36	7.89236E-15	5.01761E-12
MCM10	220651_s_at	18.35	2.9422E-15	2.14487E-12
UBE2T	223229_at	18.27	2.39159E-11	3.14327E-09
TRD@	234964_at	18.09	1.25181E-16	1.80112E-13
GTF2H2	223758_s_at	18.06	5.43298E-11	6.12471E-09
CD28	211856_x_at	18.03	1.32687E-12	3.02278E-10
GZMH	210321_at	18.02	1.2602E-07	3.52256E-06
CTH	217127_at	17.87	2.56352E-15	1.92E-12
PRPS1	208447_s_at	17.86	1.86278E-09	1.08695E-07
SEH1L	223225_s_at	17.83	1.20837E-08	5.00894E-07
CD7	214551_s_at	17.82	1.33009E-09	8.30165E-08
SLA	203760_s_at	17.72	1.60977E-09	9.6932E-08
SHMT2	214095_at	17.67	1.59355E-13	5.5851E-11
WARS	200628_s_at	17.59	6.15779E-13	1.64233E-10
LOC374443	240572_s_at	17.58	4.52534E-11	5.32092E-09
MTHFD1L	231094_s_at	17.16	8.13824E-18	1.71138E-14
HSF5	230718_at	16.99	2.74812E-16	3.49427E-13
AGFG1	213926_s_at	16.67	1.4129E-11	2.10492E-09
CDK6	243000_at	16.67	3.0899E-11	3.89264E-09
None	229629_at	16.66	7.06489E-12	1.21089E-09
TAGAP	1552542_s_at	16.63	5.34554E-11	6.05109E-09
None	223038_s_at	16.62	2.28176E-07	5.74643E-06
GBP5	238581_at	16.59	4.48253E-11	5.28195E-09
HSPH1	208744_x_at	16.58	2.56039E-10	2.22205E-08
MRPL50	225580_at	16.53	3.36224E-07	7.91349E-06
HNRNPU	216855_s_at	16.48	6.54736E-11	7.14524E-09
IL17A	216876_s_at	16.44	6.2704E-16	6.46857E-13
CACYBP	210691_s_at	16.43	6.33531E-08	1.9782E-06
RPAP3	1557984_s_at	16.37	3.12942E-11	3.92144E-09
ZAP70	1555613_a_at	16.30	4.16627E-08	1.39578E-06
NOLC1	211949_s_at	16.29	8.09132E-13	2.04812E-10
LEF1	210948_s_at	16.20	5.51317E-18	1.20573E-14
FKBP11	219118_at	16.16	3.15937E-13	9.80738E-11
TFAM	203176_s_at	16.08	1.01124E-10	1.02199E-08
ST8SIA4	242943_at	16.05	1.27105E-08	5.19782E-07

STAT4	206118_at	16.03	1.11857E-10	1.11601E-08
WDR89	235025_at	15.98	2.30793E-10	2.04515E-08
None	234396_at	15.96	2.66123E-16	3.46436E-13
TMEM170A	228505_s_at	15.95	1.38452E-11	2.08536E-09
TNFSF8	207216_at	15.92	8.83824E-11	9.01814E-09
PPP1R16B	41577_at	15.88	4.42601E-08	1.47287E-06
NOLC1	205895_s_at	15.80	1.54005E-11	2.23348E-09
SFXN1	218392_x_at	15.77	4.50644E-14	2.07455E-11
CCL5	1555759_a_at	15.76	7.04838E-07	1.47538E-05
LMAN1	203293_s_at	15.74	4.64268E-07	1.03862E-05
MAD2L1	203362_s_at	15.73	5.87981E-07	1.26367E-05
STK4	211085_s_at	15.69	6.64579E-08	2.05287E-06
RAC2	213603_s_at	15.65	7.3984E-07	1.53864E-05
SOCS1	210001_s_at	15.53	1.41368E-09	8.73665E-08
GPR18	210279_at	15.51	9.79608E-13	2.36992E-10
WDR36	238677_at	15.50	4.37413E-11	5.18776E-09
ODC1	200790_at	15.48	2.1403E-10	1.92152E-08
BTLA	236226_at	15.41	1.98069E-15	1.55064E-12
NOP16	203023_at	15.29	6.54239E-10	4.66978E-08
TMPO	209753_s_at	15.29	1.26823E-11	1.92612E-09
EXOSC3	227916_x_at	15.23	1.11452E-09	7.16246E-08
MAD2L1	1554768_a_at	15.22	1.5419E-10	1.44602E-08
LRP8	205282_at	15.20	6.69145E-12	1.15777E-09
CD5	230489_at	15.13	3.70218E-14	1.77559E-11
CREM	228092_at	14.97	4.87718E-08	1.59295E-06
None	226034_at	14.93	1.6171E-08	6.30184E-07
HSPA4	211016_x_at	14.91	1.02537E-07	2.95366E-06
TRNT1	1552625_a_at	14.81	3.21472E-13	9.81928E-11
TNFRSF18	223851_s_at	14.78	5.61948E-10	4.1352E-08
MTHFD2	201761_at	14.77	2.94324E-07	7.06726E-06
BAX	208478_s_at	14.63	1.75276E-09	1.03827E-07
TRAF3IP3	213888_s_at	14.62	1.83432E-08	7.03306E-07
RNF6	210932_s_at	14.60	1.34534E-09	8.38728E-08
UTP23	238562_at	14.59	2.74528E-08	9.84118E-07
ME2	210154_at	14.55	2.93297E-08	1.03659E-06

EED	209572_s_at	14.52	4.43461E-08	1.47393E-06
TIGIT	240070_at	14.51	1.55802E-13	5.49578E-11
IRF8	204057_at	14.49	1.35879E-07	3.75291E-06
SEPT1	227552_at	14.49	4.75507E-09	2.34642E-07
YRDC	222703_s_at	14.49	2.00551E-10	1.81542E-08
None	238950_at	14.44	3.76381E-15	2.63829E-12
NETO2	218888_s_at	14.40	3.87457E-11	4.71809E-09
SLA	203761_at	14.39	6.62832E-09	3.06861E-07
C5orf22	1552660_a_at	14.29	2.95117E-09	1.58602E-07
NETO2	222774_s_at	14.24	1.49729E-14	8.35349E-12
CCR4	208376_at	14.18	2.15603E-13	7.14431E-11
HEATR1	1556348_at	14.18	6.94817E-14	2.85633E-11
IL21R	219971_at	14.16	3.41784E-14	1.66849E-11
None	215524_x_at	14.02	1.7337E-14	9.38514E-12
DIMT1L	210802_s_at	13.97	3.85629E-08	1.30391E-06
MDFIC	1559942_at	13.93	1.71781E-15	1.41962E-12
UHRF1	225655_at	13.90	1.14665E-09	7.34113E-08
ASXL1	244519_at	13.84	1.03311E-12	2.47742E-10
TNFRSF18	224553_s_at	13.82	1.29993E-09	8.17881E-08
BACH2	221234_s_at	13.80	2.74671E-08	9.84118E-07
AKIRIN1	222458_s_at	13.80	2.45963E-07	6.11553E-06
SCLT1	1569190_at	13.77	2.82517E-20	1.80689E-16
RHOF	1554539_a_at	13.71	9.29335E-10	6.15896E-08
GINS2	221521_s_at	13.71	4.10833E-12	7.77242E-10
FEN1	204768_s_at	13.70	4.39337E-14	2.05306E-11
SRPRB	222532_at	13.70	1.19759E-10	1.18406E-08
AP1S3	1555731_a_at	13.67	1.87268E-15	1.50572E-12
RFC3	204128_s_at	13.66	1.76191E-07	4.62915E-06
FARSA	216602_s_at	13.65	4.13766E-09	2.09861E-07
SFRS6	206108_s_at	13.64	1.96098E-07	5.06935E-06
GPATCH4	224634_at	13.64	5.91197E-08	1.8733E-06
LMAN1	203294_s_at	13.58	1.0875E-08	4.59142E-07
CTDSPL2	1555106_a_at	13.55	1.05203E-07	3.01308E-06
C1orf107	214193_s_at	13.55	1.03071E-07	2.96445E-06
ZWILCH	218349_s_at	13.53	1.22012E-07	3.42981E-06

CXCR3	207681_at	13.49	1.21936E-15	1.11114E-12
BCCIP	227896_at	13.47	5.04955E-12	9.14185E-10
RRAS2	208456_s_at	13.40	4.39958E-09	2.20281E-07
C10orf78	238794_at	13.36	1.6706E-08	6.48261E-07
DDHD1	225970_at	13.35	1.90152E-07	4.94434E-06
PDSS1	220865_s_at	13.28	6.05219E-11	6.68492E-09
C12orf24	204521_at	13.27	8.20995E-13	2.05908E-10
PPPDE2	212527_at	13.27	5.1912E-09	2.51845E-07
MCM4	212142_at	13.25	1.84868E-09	1.08219E-07
CHEK1	238075_at	13.19	1.41735E-13	5.06494E-11
BCAT1	214452_at	13.15	8.94964E-13	2.18447E-10
IL27RA	222062_at	13.13	2.0843E-11	2.79998E-09
POLR3K	218866_s_at	13.08	2.97402E-14	1.51967E-11
LOC374443	238790_at	13.04	4.55319E-14	2.07455E-11
GALM	235256_s_at	13.04	3.17552E-13	9.80738E-11
IL21	221271_at	13.01	3.33674E-14	1.65636E-11
BATF	205965_at	13.00	3.33844E-10	2.71218E-08
UBE2S	202779_s_at	12.92	5.32839E-09	2.56452E-07
ZBTB32	220118_at	12.86	2.17743E-08	8.10974E-07
ACSL6	229725_at	12.85	2.94061E-16	3.57284E-13
CDC42SE2	1552612_at	12.81	4.05844E-08	1.36383E-06
IL23A	220054_at	12.79	6.42705E-10	4.59947E-08
CISH	223961_s_at	12.77	6.25893E-10	4.49681E-08
TRAF3IP3	205804_s_at	12.75	3.46129E-08	1.19323E-06
TSR1	221987_s_at	12.73	1.56693E-10	1.46447E-08
AGPS	205401_at	12.73	1.27278E-07	3.55048E-06
TDP1	219715_s_at	12.72	5.6717E-14	2.42266E-11
TMEM48	218073_s_at	12.72	6.03873E-09	2.84138E-07
RAB33A	206039_at	12.70	1.25664E-12	2.89901E-10
TRBV7-3	234883_x_at	12.65	1.22991E-11	1.88891E-09
FAM54A	228069_at	12.64	8.771E-11	9.01375E-09
PPP1R16B	212750_at	12.62	2.10924E-08	7.90423E-07
SEPT6	212415_at	12.61	3.6321E-10	2.91181E-08
AMICA1	228094_at	12.59	2.82021E-08	1.00518E-06
CREM	209967_s_at	12.55	5.20106E-10	3.90125E-08

CD7	214049_x_at	12.54	9.40843E-09	4.07612E-07
CTPS	202613_at	12.50	1.28016E-10	1.24542E-08
None	216236_s_at	12.48	4.78233E-08	1.57041E-06
SLC16A1	1557918_s_at	12.47	1.27043E-09	8.03944E-08
LAG3	206486_at	12.41	9.74047E-12	1.58973E-09
PRF1	214617_at	12.41	6.18869E-07	1.31917E-05
NCBP1	209520_s_at	12.38	9.51438E-09	4.11272E-07
IRF4	216986_s_at	12.35	4.31754E-09	2.16769E-07
SLC43A3	210692_s_at	12.34	6.82506E-10	4.82743E-08
RHOH	204951_at	12.33	1.1641E-11	1.81952E-09
DTL	218585_s_at	12.32	7.53362E-10	5.22716E-08
None	236301_at	12.26	4.39142E-13	1.23763E-10
ETS1	214447_at	12.26	4.24213E-12	7.84346E-10
C1orf174	238010_at	12.24	4.54771E-10	3.50206E-08
TSR1	218156_s_at	12.22	2.55981E-07	6.31578E-06
C12orf5	219099_at	12.20	2.21154E-07	5.60575E-06
TMEM194A	212619_at	12.19	4.36391E-11	5.18689E-09
EEF1E1	204905_s_at	12.18	2.19449E-07	5.57027E-06
RAD18	224200_s_at	12.14	1.72104E-10	1.58949E-08
BCL2L1	215037_s_at	12.14	3.33335E-09	1.75241E-07
RAB27A	222294_s_at	12.12	5.57481E-07	1.20953E-05
SLAIN1	225619_at	12.10	1.45188E-08	5.77318E-07
TBX21	220684_at	12.08	1.35004E-08	5.43162E-07
SLAMF7	222838_at	12.07	1.36763E-07	3.77081E-06
C1orf107	204700_x_at	12.07	2.52372E-07	6.25464E-06
STIP1	213330_s_at	11.97	5.97846E-09	2.81787E-07
UTP15	228050_at	11.95	5.46902E-09	2.62297E-07
NEFH	33767_at	11.95	1.46765E-12	3.30221E-10
IL8	202859_x_at	11.94	3.90848E-07	8.99773E-06
PVRIG	219812_at	11.93	7.84742E-09	3.51975E-07
CDC40	203377_s_at	11.93	2.70569E-08	9.73886E-07
SH2D1A	211210_x_at	11.92	2.54822E-10	2.21501E-08
CDC6	203968_s_at	11.92	9.49053E-14	3.72323E-11
GFI1	206589_at	11.91	4.05893E-09	2.06631E-07
RFC5	203210_s_at	11.88	3.1226E-13	9.80738E-11

METT10D	220797_at	11.85	6.60065E-10	4.70522E-08
PIM2	204269_at	11.84	5.18218E-11	5.95243E-09
ST8SIA4	230261_at	11.81	8.97935E-08	2.64091E-06
WIPF1	202663_at	11.78	5.77385E-07	1.2458E-05
ITGA4	205885_s_at	11.77	7.31423E-11	7.82594E-09
CREM	207630_s_at	11.72	1.16545E-11	1.81952E-09
PNO1	203622_s_at	11.70	3.01767E-11	3.8281E-09
NCBP2	201521_s_at	11.69	1.81952E-09	1.0697E-07
PNPT1	225291_at	11.67	3.32732E-10	2.70716E-08
C11orf82	228281_at	11.67	2.8947E-14	1.49309E-11
NPM3	205129_at	11.66	1.08807E-12	2.57533E-10
BYSL	203612_at	11.64	1.17144E-13	4.2699E-11
SLC4A5	234976_x_at	11.61	1.76366E-11	2.47887E-09
CD6	213958_at	11.61	5.5447E-09	2.65229E-07
UTP14A	221514_at	11.60	6.5107E-09	3.01927E-07
None	217185_s_at	11.58	4.0747E-12	7.73556E-10
RASSF5	1554834_a_at	11.57	3.08102E-08	1.08123E-06
IL17F	234408_at	11.56	9.39777E-09	4.07473E-07
PGAM5	1555943_at	11.53	3.29728E-15	2.37209E-12
CD6	211893_x_at	11.53	8.86735E-08	2.61219E-06
C4orf43	218513_at	11.51	7.96137E-07	1.64198E-05
None	237753_at	11.49	3.15161E-11	3.93412E-09
CTH	206085_s_at	11.48	1.42693E-14	8.12681E-12
POLR1E	231041_at	11.47	1.63299E-15	1.37816E-12
LYAR	223414_s_at	11.47	5.47893E-13	1.49035E-10
None	236280_at	11.46	5.67635E-10	4.17143E-08
TRAF1	235116_at	11.45	5.54277E-14	2.39229E-11
DNMT3A	244428_at	11.42	2.02968E-11	2.76232E-09
IL2RB	205291_at	11.41	4.44406E-09	2.21899E-07
WDHD1	216228_s_at	11.35	3.70002E-14	1.77559E-11
PSMB10	202659_at	11.34	3.11044E-08	1.08736E-06
RQCD1	1554080_at	11.32	1.90654E-08	7.26413E-07
CFLAR	210563_x_at	11.26	3.40004E-09	1.78404E-07
PDCD2L	224467_s_at	11.25	2.00527E-12	4.31646E-10
RQCD1	1553510_s_at	11.21	3.2747E-13	9.89194E-11

ZAP70	214032_at	11.18	5.47455E-11	6.14623E-09
WARS	200629_at	11.18	3.4612E-12	6.7586E-10
LPXN	216250_s_at	11.17	3.57985E-09	1.86408E-07
C16orf87	226608_at	11.15	1.85666E-08	7.1043E-07
MFNG	204153_s_at	11.14	5.08422E-09	2.48213E-07
PAK1IP1	218886_at	11.14	1.24779E-11	1.911E-09
GBP5	229625_at	11.12	8.57506E-10	5.76681E-08
LSM12	212529_at	11.12	4.44086E-08	1.47512E-06
FERMT3	223303_at	11.10	7.85115E-10	5.40632E-08
GPATCH4	224632_at	11.09	2.36283E-09	1.33046E-07
POLE2	205909_at	11.06	1.33092E-10	1.27888E-08
GART	230766_at	11.03	5.56956E-15	3.66886E-12
NDUFAF4	219006_at	11.03	4.60325E-11	5.37814E-09
ATP13A3	219558_at	11.03	5.2637E-10	3.9072E-08
None	231042_s_at	11.02	5.943E-10	4.32092E-08
PSPC1	222611_s_at	11.01	6.54082E-08	2.02617E-06
MRPS23	223156_at	11.00	4.49081E-07	1.01085E-05
WDR4	241937_s_at	10.98	2.95556E-09	1.58602E-07
MAGOHB	218894_s_at	10.94	2.92931E-08	1.03597E-06
ST6GAL1	214971_s_at	10.92	4.93259E-11	5.70167E-09
CSTF2	238821_at	10.91	1.63842E-15	1.37816E-12
CYFIP2	215785_s_at	10.91	6.41594E-09	2.98039E-07
SLAMF7	219159_s_at	10.90	5.73975E-08	1.82666E-06
AGK	218568_at	10.89	3.4175E-07	8.02972E-06
BAX	211833_s_at	10.82	5.2946E-11	6.03088E-09
None	231894_at	10.80	6.29693E-09	2.94261E-07
POMP	222402_at	10.77	4.8394E-07	1.07646E-05
SLC25A33	223296_at	10.75	2.75091E-08	9.84331E-07
IKZF1	216901_s_at	10.73	6.85963E-11	7.44147E-09
WDR4	226882_x_at	10.66	2.60454E-10	2.24966E-08
FIGNL1	1552921_a_at	10.65	1.28543E-10	1.24832E-08
None	212563_at	10.57	3.07384E-10	2.56187E-08
UCHL5	220083_x_at	10.55	5.19579E-10	3.90125E-08
GART	212378_at	10.51	1.04579E-09	6.82321E-08
FXN	205565_s_at	10.51	1.51729E-11	2.21221E-09

TWISTNB	214729_at	10.49	8.47394E-13	2.10597E-10
BCLAF1	201101_s_at	10.44	5.65951E-07	1.22645E-05
SLC2A3	202498_s_at	10.41	2.58765E-07	6.37584E-06
NUP50	218295_s_at	10.38	1.05334E-08	4.46791E-07
FKBP4	200894_s_at	10.37	6.23318E-09	2.9153E-07
None	204896_s_at	10.37	5.79399E-10	4.23511E-08
LRRC8C	223533_at	10.37	3.67299E-08	1.25356E-06
None	230399_at	10.36	1.67237E-07	4.44515E-06
HSPE1	205133_s_at	10.35	2.57076E-08	9.3331E-07
PPP2R1B	202886_s_at	10.35	3.47832E-07	8.15162E-06
OGFOD1	225106_s_at	10.35	9.64363E-11	9.76418E-09
LARP4	238960_s_at	10.34	2.97187E-11	3.79642E-09
ITGB1	1561042_at	10.34	1.30258E-16	1.82611E-13
STK4	205411_at	10.31	2.89973E-13	9.2176E-11
LIF	205266_at	10.30	1.96786E-12	4.25678E-10
RUVBL1	201614_s_at	10.28	1.29723E-12	2.98008E-10
PGGT1B	206288_at	10.28	3.38822E-13	1.01786E-10
GEMIN6	219539_at	10.27	1.61678E-11	2.32624E-09
IL23R	1552912_a_at	10.26	9.10991E-12	1.50479E-09
BRIX1	219177_at	10.26	3.40927E-08	1.17976E-06
BUB1	209642_at	10.22	2.7503E-11	3.56333E-09
F5	204713_s_at	10.20	1.10266E-08	4.64468E-07
C1orf55	1553338_at	10.20	1.51003E-09	9.26608E-08
SH2D1A	211211_x_at	10.16	4.07814E-07	9.33328E-06
MAGOH	210093_s_at	10.15	3.2978E-07	7.78865E-06
KIF2A	203086_at	10.14	1.23611E-08	5.09686E-07
TXNL4B	222748_s_at	10.12	1.12088E-11	1.76104E-09
G3BP2	206383_s_at	10.11	2.57448E-08	9.34039E-07
CYorf15A	236694_at	10.06	3.13143E-07	7.46668E-06
GNAS	214157_at	10.05	3.19289E-13	9.80738E-11
AGPAT5	218096_at	10.02	6.04657E-08	1.90655E-06
MPP6	205429_s_at	10.01	1.29052E-14	7.42728E-12
ORC1L	205085_at	10.01	2.031E-11	2.76232E-09
GABPA	210188_at	10.00	2.14152E-08	8.00325E-07
NUDCD1	225439_at	9.99	4.50127E-09	2.24345E-07

FAM119A	235177_at	9.99	7.69057E-11	8.13619E-09
CD6	208602_x_at	9.98	6.43006E-07	1.36119E-05
TNIP3	220655_at	9.97	6.14743E-14	2.60551E-11
STK17B	217503_at	9.96	1.08285E-07	3.0868E-06
TSEN15	225400_at	9.94	2.8429E-12	5.71455E-10
PTPN11	205868_s_at	9.94	4.43456E-08	1.47393E-06
RBL1	1559307_s_at	9.93	5.53825E-11	6.19231E-09
PVT1	1558290_a_at	9.90	9.42399E-09	4.07962E-07
DDX20	224315_at	9.87	2.9969E-09	1.60015E-07
MCM6	201930_at	9.87	1.77215E-07	4.65158E-06
RBM14	1555639_a_at	9.87	2.35586E-08	8.6914E-07
PHB	200658_s_at	9.86	3.0353E-10	2.53367E-08
CD96	1555120_at	9.85	1.22232E-13	4.39673E-11
STAT1	AFFX-HUMISGF3A/M97935_MA_at	9.81	2.66989E-09	1.46122E-07
RRP9	204133_at	9.81	1.56204E-09	9.49141E-08
BCL2L11	208536_s_at	9.81	2.38439E-09	1.33867E-07
MYO1G	227799_at	9.81	2.33823E-07	5.85427E-06
CDC23	223651_x_at	9.79	5.5932E-11	6.24099E-09
PNP	201695_s_at	9.79	3.80813E-08	1.29082E-06
NOP56	200875_s_at	9.78	5.55674E-10	4.11116E-08
SH2D1A	211209_x_at	9.74	5.4402E-07	1.1855E-05
None	232687_at	9.73	2.23585E-09	1.27074E-07
PHLDA1	217997_at	9.73	4.68664E-09	2.31481E-07
HAUS2	229181_s_at	9.72	1.60178E-09	9.65571E-08
NFKB2	207535_s_at	9.72	3.09805E-07	7.39999E-06
C17orf96	228066_at	9.71	8.87333E-10	5.92368E-08
MFNG	204152_s_at	9.71	2.32977E-10	2.06117E-08
BCLAF1	214499_s_at	9.70	4.08159E-07	9.33728E-06
FIP1L1	1554424_at	9.70	3.11439E-10	2.57609E-08
PLAGL2	202924_s_at	9.69	4.22133E-10	3.29716E-08
GLS	203158_s_at	9.67	7.0696E-09	3.23186E-07
PSMA7	216088_s_at	9.64	1.12768E-08	4.73184E-07
None	230966_at	9.64	4.75347E-10	3.63491E-08
KLHL6	1555275_a_at	9.61	9.93438E-14	3.84087E-11
ACP1	201629_s_at	9.61	7.24501E-07	1.51232E-05

None	235573_at	9.61	7.0584E-11	7.62684E-09
NUDT21	202697_at	9.59	3.63565E-07	8.46589E-06
C6orf129	225723_at	9.55	4.30458E-09	2.16317E-07
None	209881_s_at	9.54	4.38532E-11	5.18977E-09
TIMM8A	205217_at	9.54	3.3627E-14	1.65636E-11
CFLAR	209939_x_at	9.53	3.50581E-10	2.82298E-08
ALG13	219015_s_at	9.52	1.48427E-07	4.02541E-06
YDD19	37079_at	9.52	6.55989E-12	1.13861E-09
TMEM33	222642_s_at	9.51	6.78444E-07	1.42614E-05
HAUS6	233655_s_at	9.47	1.23402E-10	1.21568E-08
RAB27A	209515_s_at	9.40	5.46081E-08	1.75217E-06
GRPEL2	238427_at	9.40	9.32296E-12	1.53073E-09
GUF1	218884_s_at	9.40	1.60394E-08	6.26408E-07
ZNF267	219540_at	9.39	7.84351E-07	1.62072E-05
RANBP1	202483_s_at	9.38	2.45916E-09	1.37059E-07
EZH2	203358_s_at	9.36	2.804E-07	6.79258E-06
HSPA9	200690_at	9.31	2.67235E-08	9.63773E-07
SRM	201516_at	9.30	4.07967E-09	2.07491E-07
PRMT1	206445_s_at	9.29	2.4344E-08	8.93295E-07
GSR	205770_at	9.29	5.2551E-11	6.00077E-09
MCM2	202107_s_at	9.28	2.24261E-08	8.31766E-07
SUV39H2	1554572_a_at	9.27	2.73583E-09	1.49134E-07
UHRF1BP1L	1554292_a_at	9.27	2.37738E-15	1.80532E-12
SEPT6	1555526_a_at	9.26	8.5789E-09	3.76446E-07
FKBP11	219117_s_at	9.23	2.36254E-10	2.08051E-08
LRP8	208433_s_at	9.23	1.28735E-08	5.25176E-07
GTPBP4	218239_s_at	9.22	1.57126E-09	9.53479E-08
IL8	211506_s_at	9.21	6.79661E-08	2.09001E-06
PTPN11	205867_at	9.19	5.58612E-10	4.12174E-08
CLEC2D	235522_at	9.18	1.79275E-11	2.50047E-09
SNRPD1	202691_at	9.18	1.5619E-11	2.25321E-09
SENP1	1552812_a_at	9.16	1.30478E-11	1.97615E-09
None	218982_s_at	9.16	2.43547E-11	3.19327E-09
AK2	205996_s_at	9.15	1.55938E-09	9.49141E-08
CARS	240983_s_at	9.15	3.67722E-08	1.25422E-06

None	227458_at	9.10	6.86894E-10	4.83968E-08
CDC25A	1555772_a_at	9.09	5.27901E-16	5.77259E-13
UTP11L	218235_s_at	9.08	1.70077E-10	1.57343E-08
WDR12	218512_at	9.08	4.67654E-11	5.4402E-09
VMA21	242474_s_at	9.07	2.12307E-12	4.53434E-10
CPSF2	233208_x_at	9.07	4.50534E-10	3.47923E-08
PRMT5	1564520_s_at	9.05	1.54075E-10	1.44602E-08
UTP23	238561_s_at	9.04	1.30786E-09	8.21922E-08
None	218680_x_at	9.04	1.29006E-08	5.25197E-07
GK	207387_s_at	9.03	1.36715E-08	5.4843E-07
MTAP	211363_s_at	9.02	3.06354E-11	3.87729E-09
None	206785_s_at	9.02	2.08895E-08	7.85511E-07
ACSL6	211207_s_at	9.00	2.46769E-13	7.98349E-11
None	243154_at	9.00	4.75262E-13	1.31237E-10
CDKN2AIPNL	235006_at	8.99	6.42063E-11	7.02096E-09
THEMIS	1558972_s_at	8.99	1.25214E-10	1.2247E-08
KLRD1	210606_x_at	8.98	1.60397E-08	6.26408E-07
CXCR3	217119_s_at	8.97	5.79357E-08	1.84058E-06
INPP5F	230363_s_at	8.97	1.62292E-10	1.51422E-08
SP140	207777_s_at	8.97	2.47124E-10	2.1653E-08
UCK2	209825_s_at	8.96	4.21855E-11	5.04702E-09
ITGA4	205884_at	8.93	2.14682E-11	2.86987E-09
PDE12	1554915_a_at	8.91	6.34639E-08	1.98053E-06
STARD4	226390_at	8.91	1.92812E-09	1.11895E-07
SIKE1	204666_s_at	8.91	2.33277E-09	1.31624E-07
PYHIN1	240413_at	8.90	1.41416E-09	8.73665E-08
SFXN4	229236_s_at	8.90	5.8518E-11	6.50299E-09
FEN1	204767_s_at	8.89	1.20175E-13	4.35137E-11
AP4S1	210277_at	8.87	2.8781E-09	1.55801E-07
MRTO4	220688_s_at	8.87	2.72038E-10	2.32039E-08
MIR17HG	232291_at	8.85	5.1512E-08	1.67047E-06
GSPT1	201912_s_at	8.85	1.50446E-08	5.94337E-07
MTP18	223172_s_at	8.84	4.8531E-08	1.58812E-06
SYNCRIP	217834_s_at	8.84	3.68171E-07	8.56221E-06
LARP4	1555384_a_at	8.82	1.00457E-09	6.60158E-08

SKP2	210567_s_at	8.82	6.76676E-09	3.12477E-07
ABCF2	207622_s_at	8.80	4.06433E-12	7.73556E-10
NIP7	219031_s_at	8.80	4.16137E-07	9.49306E-06
ERAP2	1554273_a_at	8.79	1.71311E-12	3.77679E-10
ZNF239	206261_at	8.77	3.13428E-11	3.92144E-09
RBL1	1555004_a_at	8.77	6.9583E-10	4.89007E-08
UBFD1	205687_at	8.76	1.15513E-09	7.3781E-08
HSPA9	200692_s_at	8.76	6.26989E-08	1.96001E-06
CENPN	222118_at	8.75	1.31127E-15	1.17531E-12
C8orf33	222551_s_at	8.74	1.7832E-09	1.05516E-07
RBM8A	222443_s_at	8.74	3.07794E-11	3.88652E-09
SYNCRIP	1555427_s_at	8.72	4.89561E-09	2.40276E-07
WDR3	218882_s_at	8.72	3.06337E-09	1.63031E-07
NIP7	223397_s_at	8.72	4.73946E-08	1.55727E-06
NBN	217299_s_at	8.69	6.27354E-08	1.96003E-06
RHOF	219045_at	8.69	7.45852E-10	5.18163E-08
TNPO1	225766_s_at	8.68	3.7844E-07	8.76746E-06
PRIM1	205053_at	8.65	4.45249E-12	8.16912E-10
SHMT2	214096_s_at	8.64	9.97537E-14	3.84087E-11
QRSL1	241933_at	8.64	1.61804E-09	9.73225E-08
UBE2G2	209041_s_at	8.63	1.73562E-08	6.69216E-07
PSME3	209853_s_at	8.63	3.87761E-07	8.93795E-06
None	231715_s_at	8.61	3.71167E-09	1.91629E-07
N6AMT1	220311_at	8.60	5.60374E-16	6.00754E-13
TRD@	213830_at	8.59	2.47552E-09	1.3783E-07
SBNO1	218737_at	8.58	7.11973E-10	4.9779E-08
TRAF4	211899_s_at	8.58	2.86223E-10	2.41501E-08
PRKCQ	210038_at	8.57	1.50642E-08	5.94682E-07
RGS16	209325_s_at	8.57	6.96684E-08	2.13636E-06
MARS	213671_s_at	8.56	3.61268E-08	1.23762E-06
PRIM2	1554885_a_at	8.56	3.66475E-12	7.08022E-10
GNA13	206917_at	8.56	7.53283E-07	1.56125E-05
None	207247_s_at	8.55	4.04435E-13	1.15772E-10
IL23R	1561853_a_at	8.55	2.69493E-11	3.49989E-09
NUP50	222583_s_at	8.54	3.21842E-09	1.69689E-07

DDX39	201584_s_at	8.53	3.86381E-13	1.12369E-10
TOMM22	217960_s_at	8.52	1.04144E-07	2.99216E-06
None	1557733_a_at	8.50	3.85227E-13	1.12369E-10
LARP4	238959_at	8.48	1.45571E-11	2.13954E-09
CENPN	228559_at	8.48	1.16809E-11	1.81952E-09
LSM2	209449_at	8.48	2.52772E-12	5.21522E-10
FAM40B	1555292_at	8.47	4.97318E-14	2.21064E-11
XCL1	206365_at	8.45	2.07144E-18	5.148E-15
NUTF2	202397_at	8.44	2.63942E-07	6.48585E-06
TOMM22	222474_s_at	8.42	4.82267E-08	1.58081E-06
HSF2	211220_s_at	8.41	1.28574E-09	8.11751E-08
None	233746_x_at	8.40	6.9579E-09	3.19415E-07
WDR75	224721_at	8.39	1.13282E-08	4.74977E-07
THEMIS	1558971_at	8.37	1.8568E-08	7.1043E-07
GPCPD1	230492_s_at	8.36	2.18494E-07	5.55376E-06
JAK3	227677_at	8.34	1.97575E-08	7.49649E-07
GFM1	232296_s_at	8.34	8.89431E-10	5.93045E-08
PSME3	200987_x_at	8.32	2.08506E-07	5.32962E-06
SOCS1	209999_x_at	8.31	7.5354E-09	3.40494E-07
DDX3Y	1570360_s_at	8.30	3.92783E-10	3.10788E-08
TRD@	216133_at	8.30	6.09981E-12	1.07237E-09
EXOSC2	214507_s_at	8.29	4.0657E-12	7.73556E-10
EEF1E1	213907_at	8.29	4.94285E-10	3.74827E-08
NME1	201577_at	8.28	5.52567E-09	2.64551E-07
C13orf27	213346_at	8.28	6.97526E-07	1.46232E-05
MRPL12	203931_s_at	8.27	1.5849E-07	4.2603E-06
EBNA1BP2	201323_at	8.26	5.32257E-07	1.16532E-05
GADD45GIP1	212891_s_at	8.25	9.3452E-08	2.73235E-06
ST8SIA4	206925_at	8.24	1.41256E-11	2.10492E-09
YRDC	218647_s_at	8.23	4.84138E-07	1.07646E-05
SPCS3	222753_s_at	8.22	4.87276E-08	1.59246E-06
GOSR2	210009_s_at	8.21	1.38343E-07	3.80671E-06
CD96	206761_at	8.20	9.6593E-15	5.93396E-12
GSPT1	217595_at	8.20	8.60552E-11	8.92301E-09
C9orf114	218565_at	8.20	1.51665E-10	1.42724E-08

MRPS12	204331_s_at	8.18	2.7298E-07	6.64818E-06
RABGGTB	209180_at	8.17	8.47387E-09	3.72734E-07
TRMT1	210463_x_at	8.16	1.06477E-10	1.06819E-08
RABL3	226089_at	8.13	3.52111E-07	8.23777E-06
RCAN3	219864_s_at	8.12	1.93336E-11	2.66936E-09
MTAP	204956_at	8.12	4.3303E-10	3.37263E-08
SNRPD1	202690_s_at	8.10	4.87154E-08	1.59246E-06
CDC25A	204695_at	8.10	3.23565E-08	1.12609E-06
MCOLN2	1555465_at	8.09	6.35413E-14	2.652E-11
KCTD5	222645_s_at	8.09	1.25728E-07	3.51619E-06
RPAIN	216962_at	8.08	1.23107E-10	1.21496E-08
C6orf150	1559051_s_at	8.08	7.24462E-09	3.29808E-07
TTK	204822_at	8.05	7.77983E-07	1.60878E-05
MIER1	1555105_a_at	8.05	4.01569E-09	2.04811E-07
None	216575_at	8.05	1.73933E-13	5.98099E-11
C1QBP	208910_s_at	8.04	3.90678E-07	8.9976E-06
NUP62	207740_s_at	8.02	1.99571E-08	7.55125E-07
CIRH1A	230656_s_at	7.99	1.24276E-08	5.10888E-07
ETNK1	224453_s_at	7.97	5.87956E-11	6.52059E-09
KIAA0020	203712_at	7.95	7.82585E-12	1.3247E-09
None	1557624_at	7.95	3.07846E-10	2.56187E-08
TRMT1	203701_s_at	7.95	1.55836E-11	2.25321E-09
PPAT	209434_s_at	7.94	1.52497E-09	9.33681E-08
ARPP19	214553_s_at	7.93	5.96099E-09	2.81498E-07
CCND3	201700_at	7.92	3.10656E-09	1.64425E-07
MARS2	243529_at	7.90	2.15025E-08	8.0249E-07
BOLA3	227291_s_at	7.89	6.49232E-07	1.37212E-05
TIMM50	224913_s_at	7.87	1.92483E-10	1.754E-08
PINX1	223907_s_at	7.87	5.25765E-09	2.54166E-07
ARL6	223735_at	7.86	3.27567E-12	6.41925E-10
STX11	210190_at	7.86	4.3023E-08	1.43695E-06
TARS	201263_at	7.85	7.13106E-09	3.25451E-07
ZFR	33148_at	7.84	1.04424E-07	2.99862E-06
TSEN15	230257_s_at	7.84	2.61725E-10	2.25707E-08
FAM113B	228298_at	7.83	3.00943E-07	7.2072E-06

GBP4	235574_at	7.80	3.14666E-12	6.25613E-10
YKT6	217785_s_at	7.80	8.27955E-08	2.46158E-06
UBE3C	1554794_a_at	7.79	1.91428E-08	7.28709E-07
MRPL17	222216_s_at	7.79	2.03549E-10	1.83951E-08
LARS	223888_s_at	7.75	1.55788E-08	6.12579E-07
RPF2	225866_at	7.75	3.55531E-07	8.30358E-06
EIF2S1	201144_s_at	7.73	8.97855E-11	9.14157E-09
OSM	230170_at	7.73	1.90573E-07	4.95228E-06
None	206499_s_at	7.72	4.59263E-11	5.37814E-09
C16orf61	218447_at	7.69	5.27625E-10	3.90893E-08
MRPS7	217932_at	7.69	1.46635E-09	9.01827E-08
PRKCQ	210039_s_at	7.69	7.92504E-08	2.37426E-06
SSSCA1	203114_at	7.68	1.35515E-12	3.07439E-10
KIAA1704	220171_x_at	7.68	6.71967E-08	2.06868E-06
CALCB	214636_at	7.67	2.66197E-10	2.28842E-08
GK	215977_x_at	7.66	8.17289E-10	5.55787E-08
PFDN2	218336_at	7.64	8.25438E-10	5.59936E-08
SEMA7A	230345_at	7.63	7.22561E-11	7.7615E-09
EMG1	209233_at	7.63	1.85349E-10	1.69464E-08
RASAL3	228677_s_at	7.63	4.56903E-07	1.0255E-05
None	234852_at	7.61	3.18782E-13	9.80738E-11
CLEC2D	228426_at	7.61	3.89993E-13	1.12476E-10
MCM5	216237_s_at	7.59	2.95593E-09	1.58602E-07
SYT11	209198_s_at	7.59	1.12096E-12	2.64175E-10
DDX18	208897_s_at	7.59	1.27904E-07	3.55885E-06
FAM169A	235048_at	7.58	2.88196E-08	1.02252E-06
CBX5	209715_at	7.57	2.30631E-07	5.78961E-06
MYCBP	203360_s_at	7.56	2.67754E-07	6.55889E-06
IL26	221111_at	7.56	5.55685E-14	2.39229E-11
MRPL4	223743_s_at	7.56	4.65933E-08	1.53463E-06
PSMC4	201252_at	7.56	8.44744E-12	1.40812E-09
None	207891_s_at	7.55	2.58852E-10	2.23935E-08
GOLT1B	218193_s_at	7.53	4.2224E-07	9.60314E-06
ORC5L	211212_s_at	7.52	1.60352E-08	6.26408E-07
ATAD2	218782_s_at	7.51	2.09793E-08	7.87805E-07

XRN1	1555785_a_at	7.50	1.40094E-11	2.09853E-09
AP4S1	210278_s_at	7.50	1.54285E-09	9.4357E-08
WDR77	201420_s_at	7.49	1.23474E-14	7.25171E-12
DNLZ	228272_at	7.48	2.65081E-11	3.45079E-09
CASP2	209811_at	7.47	1.89568E-11	2.63479E-09
BCCIP	218264_at	7.47	4.8488E-07	1.07724E-05
RPIA	212973_at	7.47	2.51798E-07	6.24357E-06
None	221513_s_at	7.47	1.80692E-09	1.06352E-07
HNRPLL	1554453_at	7.46	1.02193E-13	3.90727E-11
PPAN	221649_s_at	7.46	5.04451E-08	1.64172E-06
THEM4	243492_at	7.46	5.11039E-11	5.88233E-09
RSC1A1	214583_at	7.46	9.37784E-09	4.06931E-07
PLAGL2	202925_s_at	7.45	1.11374E-09	7.16246E-08
F5	231029_at	7.44	6.67063E-08	2.05822E-06
TDRKH	223530_at	7.44	2.74993E-12	5.5893E-10
MCM6	238977_at	7.43	1.98527E-15	1.55064E-12
NEDD1	1552417_a_at	7.43	4.81522E-09	2.36543E-07
NFKB2	209636_at	7.41	1.97192E-07	5.08828E-06
POLR3G	206653_at	7.40	1.11481E-09	7.16246E-08
SGOL2	230165_at	7.37	4.70404E-08	1.54843E-06
C3orf26	224523_s_at	7.35	3.71739E-07	8.62685E-06
KIAA1586	231869_at	7.35	2.05673E-10	1.85258E-08
TIA1	1554890_a_at	7.35	5.2743E-07	1.15766E-05
ZC3H8	223506_at	7.35	1.71426E-07	4.5257E-06
SNRPA1	216977_x_at	7.34	7.4502E-09	3.38322E-07
CACYBP	201381_x_at	7.34	1.92853E-07	5.00201E-06
PSPH	205194_at	7.33	2.49478E-09	1.38437E-07
SEC23IP	216392_s_at	7.33	1.01692E-07	2.93404E-06
GK	217167_x_at	7.32	6.95834E-10	4.89007E-08
RABEP1	203223_at	7.32	1.64955E-09	9.88914E-08
BCL2L11	222343_at	7.31	4.8714E-14	2.18315E-11
RIOK1	224450_s_at	7.30	1.4335E-07	3.91296E-06
PTPN6	206687_s_at	7.29	1.36388E-07	3.76238E-06
TBRG4	220789_s_at	7.28	1.34081E-11	2.0251E-09
C1orf128	223123_s_at	7.28	6.26007E-08	1.95806E-06

COX17	203880_at	7.28	1.30421E-08	5.30115E-07
NOP16	214011_s_at	7.27	7.62491E-08	2.29819E-06
C1QBP	214214_s_at	7.25	4.69287E-10	3.5936E-08
KIAA1429	243927_x_at	7.25	4.41118E-10	3.42587E-08
C17orf75	203830_at	7.25	9.56037E-08	2.77743E-06
BRCC3	216521_s_at	7.24	3.17653E-07	7.55459E-06
MPHOSPH9	206205_at	7.24	1.65459E-10	1.53852E-08
MPDU1	209208_at	7.24	8.48442E-09	3.72899E-07
MRPL44	222555_s_at	7.23	2.27277E-07	5.72645E-06
HELLS	223556_at	7.22	1.48707E-08	5.8886E-07
MCM5	201755_at	7.18	9.93074E-13	2.39191E-10
LPIN2	202460_s_at	7.17	2.15149E-09	1.23047E-07
CXorf15	219969_at	7.17	6.38103E-09	2.96669E-07
NUDT4	206303_s_at	7.17	2.99592E-10	2.5123E-08
PPP2R1B	202884_s_at	7.16	1.75797E-07	4.62546E-06
RNGTT	204207_s_at	7.15	4.52189E-08	1.49658E-06
TIMM17A	201821_s_at	7.13	4.43404E-07	1.00013E-05
SLC19A1	211576_s_at	7.13	2.70735E-10	2.3165E-08
KIAA1826	223799_at	7.13	8.37524E-09	3.69884E-07
DCUN1D5	223151_at	7.12	5.61366E-10	4.1352E-08
GART	210005_at	7.10	5.64504E-09	2.68618E-07
None	211005_at	7.09	4.24697E-09	2.13815E-07
CCND2	200953_s_at	7.09	3.96209E-07	9.09434E-06
CCDC86	203119_at	7.09	3.58998E-08	1.23216E-06
CCDC75	1559893_at	7.08	5.01618E-10	3.79861E-08
MCM7	210983_s_at	7.08	8.37488E-11	8.72184E-09
RBL1	205296_at	7.08	5.22174E-10	3.90269E-08
CENPQ	219294_at	7.07	8.32187E-08	2.47013E-06
SNRPA1	215722_s_at	7.06	1.73786E-10	1.60001E-08
NAPG	210048_at	7.04	6.83201E-09	3.14692E-07
AASDHPPT	202170_s_at	7.04	7.7263E-07	1.59892E-05
C12orf73	226943_at	7.03	6.16784E-10	4.43784E-08
LOC387895	1560573_at	7.02	3.60883E-07	8.4142E-06
APOBEC3B	206632_s_at	7.02	5.0477E-07	1.11463E-05
CSNK2A1	212075_s_at	7.00	3.7088E-08	1.26205E-06

RHOF	222812_s_at	7.00	1.06396E-08	4.50595E-07
EXOSC3	227912_s_at	7.00	2.23595E-10	1.9943E-08
HTATSF1	202601_s_at	6.99	2.15138E-07	5.47611E-06
POLR2D	203664_s_at	6.98	9.84565E-12	1.60212E-09
PIK3CG	206369_s_at	6.97	4.99195E-12	9.07973E-10
C1GALT1	219439_at	6.97	5.36194E-07	1.17078E-05
KLRD1	207795_s_at	6.97	2.92009E-07	7.03108E-06
LSM6	205036_at	6.96	3.48119E-07	8.15485E-06
LOC100287482	235736_at	6.96	7.36101E-14	2.98121E-11
HSPA4	208815_x_at	6.96	8.60259E-09	3.77183E-07
PATL1	235234_at	6.95	1.0167E-09	6.67323E-08
LMNB1	203276_at	6.94	5.35839E-07	1.17048E-05
RRP7A	202937_x_at	6.93	1.58914E-07	4.2696E-06
MTMR2	214649_s_at	6.93	6.72592E-07	1.41656E-05
C18orf19	235022_at	6.93	1.34993E-08	5.43162E-07
None	209836_x_at	6.92	2.22933E-09	1.26835E-07
BATF3	220358_at	6.92	3.22865E-09	1.70064E-07
BGLAP	206956_at	6.92	3.7117E-08	1.26205E-06
C10orf2	218590_at	6.91	2.25744E-10	2.01018E-08
ELAVL1	201727_s_at	6.91	7.00524E-08	2.14572E-06
MARS	201475_x_at	6.90	2.35216E-14	1.23658E-11
RBBP4	217301_x_at	6.90	6.14335E-08	1.92928E-06
ME2	210153_s_at	6.89	4.12011E-10	3.23505E-08
POLR1C	207515_s_at	6.89	1.76103E-07	4.62904E-06
CTU2	226410_at	6.89	1.1207E-07	3.18142E-06
CD40LG	207892_at	6.89	1.20888E-11	1.8724E-09
None	213454_at	6.88	5.26677E-10	3.9072E-08
HSPA9	200691_s_at	6.88	3.93298E-08	1.32738E-06
FUBP1	203091_at	6.88	1.27382E-10	1.24146E-08
DNAJC18	227166_at	6.87	1.27408E-09	8.05323E-08
TNFSF8	241819_at	6.85	6.70326E-13	1.73697E-10
EAF2	219551_at	6.85	1.86026E-09	1.08664E-07
ATIC	208758_at	6.84	5.5257E-09	2.64551E-07
SLC38A5	234973_at	6.84	4.13578E-08	1.38726E-06
LZIC	226087_at	6.83	4.38978E-08	1.46348E-06

C12orf60	229888_at	6.83	1.45006E-11	2.13698E-09
NUP160	214962_s_at	6.82	1.7879E-07	4.68392E-06
MTAP	216685_s_at	6.82	7.73934E-09	3.48557E-07
WDR89	244038_at	6.81	1.42403E-08	5.66659E-07
TERF2	229790_at	6.81	2.51876E-12	5.21522E-10
TRAF4	235688_s_at	6.80	1.03676E-09	6.79677E-08
MINA	1554774_at	6.80	2.90665E-11	3.73055E-09
TFAM	208541_x_at	6.80	3.38254E-07	7.95099E-06
NCRNA00158	231303_at	6.80	5.17801E-15	3.45253E-12
GIGYF2	1558305_at	6.79	1.57474E-15	1.36665E-12
GTF2H4	203577_at	6.78	2.50107E-10	2.18096E-08
IMP4	212411_at	6.78	1.43654E-11	2.12854E-09
GTPBP3	1555062_s_at	6.77	1.61382E-08	6.29357E-07
ZNF101	1552634_a_at	6.76	3.01146E-11	3.8281E-09
SLC25A19	223222_at	6.76	5.21263E-09	2.52436E-07
TXNRD1	201266_at	6.76	3.10256E-10	2.57409E-08
CDV3	213554_s_at	6.76	1.10657E-09	7.14306E-08
METTL1	204027_s_at	6.75	1.18855E-12	2.75356E-10
FAM40B	231880_at	6.75	6.63709E-13	1.73331E-10
SLC7A6	203578_s_at	6.75	4.11781E-10	3.23505E-08
WDR5	223308_s_at	6.74	5.04386E-10	3.81429E-08
FBXO22	225737_s_at	6.74	9.16546E-08	2.68698E-06
CISH	223377_x_at	6.72	3.97145E-09	2.03224E-07
KPNA2	211762_s_at	6.72	8.82921E-10	5.90865E-08
EXOSC3	223489_x_at	6.71	2.35233E-10	2.07777E-08
ADSL	210250_x_at	6.71	2.14535E-09	1.22824E-07
PRIM2	215708_s_at	6.71	5.04487E-09	2.46716E-07
C17orf81	219260_s_at	6.70	2.34819E-08	8.66896E-07
DISP1	235466_s_at	6.68	1.38591E-08	5.55125E-07
CBFB	206788_s_at	6.68	1.22776E-07	3.44775E-06
PDCD5	219275_at	6.67	1.2302E-07	3.45283E-06
None	215207_x_at	6.66	2.7089E-07	6.60907E-06
LCP2	244251_at	6.66	4.8318E-13	1.32753E-10
None	1560156_at	6.66	3.65498E-11	4.4907E-09
PRPS1	209440_at	6.66	8.17741E-08	2.43917E-06

EIF4EBP1	221539_at	6.65	7.79081E-08	2.33789E-06
GRPEL1	212432_at	6.65	3.50465E-09	1.82841E-07
SMARCA5	213859_x_at	6.64	1.73345E-07	4.56974E-06
HSPA14	219212_at	6.64	7.52836E-07	1.56125E-05
THOC6	218848_at	6.63	1.97203E-07	5.08828E-06
BACH2	227173_s_at	6.62	2.29572E-09	1.29936E-07
SLC3A2	200924_s_at	6.62	1.94168E-09	1.12409E-07
MRPS10	224247_s_at	6.61	1.17567E-08	4.8882E-07
RBM22	222527_s_at	6.60	2.63362E-09	1.44571E-07
None	215570_s_at	6.60	1.46733E-10	1.39084E-08
OIP5	213599_at	6.58	1.69378E-07	4.48244E-06
LAX1	207734_at	6.56	5.32016E-09	2.56367E-07
PFDN6	222029_x_at	6.56	8.6342E-11	8.92301E-09
RAD51C	206066_s_at	6.55	2.36483E-11	3.11559E-09
RPUSD3	1566603_s_at	6.54	4.30811E-12	7.93083E-10
None	244598_at	6.54	8.11327E-10	5.5242E-08
CCT5	208696_at	6.54	5.8031E-12	1.03014E-09
SLA2	1555688_s_at	6.54	3.06531E-09	1.63031E-07
TFDP1	242939_at	6.53	5.67234E-11	6.3164E-09
SLA2	232234_at	6.52	3.16641E-07	7.54202E-06
POU2F2	228343_at	6.52	9.31665E-12	1.53073E-09
SKA2	225686_at	6.51	2.82382E-07	6.83453E-06
DENR	234347_s_at	6.51	1.99904E-11	2.73928E-09
API5	201686_x_at	6.49	2.43721E-07	6.07081E-06
GNPDA1	202382_s_at	6.49	1.96518E-07	5.07779E-06
WDR67	214061_at	6.49	7.1507E-07	1.49451E-05
PSMA5	201274_at	6.48	1.45397E-08	5.77732E-07
NPM1	221923_s_at	6.48	2.00206E-07	5.14393E-06
LOC256021	1564150_a_at	6.48	1.72233E-11	2.44594E-09
DIMT1L	204405_x_at	6.47	8.67302E-08	2.56184E-06
SFRS2IP	235579_at	6.45	3.17485E-07	7.55459E-06
SIKE1	235294_at	6.45	2.70488E-07	6.6022E-06
TNFSF11	210643_at	6.45	5.43488E-09	2.60889E-07
THUMPD3	225730_s_at	6.44	6.25148E-07	1.3309E-05
UTP15	228043_at	6.44	1.32175E-09	8.25903E-08

CREM	214508_x_at	6.43	1.4198E-07	3.88526E-06
AGMAT	222930_s_at	6.43	6.21556E-08	1.94637E-06
TRIP13	204033_at	6.42	3.35518E-10	2.72173E-08
TMEM38B	222736_s_at	6.42	6.8397E-11	7.4346E-09
PRMT5	217786_at	6.41	3.61777E-12	7.01423E-10
CIRH1A	224903_at	6.41	6.4785E-13	1.71117E-10
C8orf76	225702_at	6.41	3.88935E-08	1.31347E-06
RBM19	205115_s_at	6.40	6.69543E-11	7.29228E-09
ZNF593	204175_at	6.40	3.47005E-09	1.81381E-07
RAD51	205024_s_at	6.40	1.29371E-10	1.25414E-08
PSMG1	203405_at	6.39	1.87107E-07	4.88076E-06
IFRD2	209100_at	6.39	3.23722E-10	2.64963E-08
PRPF4	209162_s_at	6.38	3.28287E-08	1.14035E-06
ZNF643	207219_at	6.33	1.80658E-12	3.95099E-10
NOP2	214427_at	6.33	5.48096E-08	1.75657E-06
DPP3	218567_x_at	6.32	1.4539E-09	8.96192E-08
RPP40	213427_at	6.31	2.37983E-10	2.09192E-08
PLD6	227037_at	6.30	1.42134E-08	5.66001E-07
PASK	213534_s_at	6.30	8.18635E-08	2.44031E-06
RILPL2	227983_at	6.29	3.67982E-07	8.56145E-06
None	234488_s_at	6.29	2.6968E-07	6.59427E-06
SERPINB9	209722_s_at	6.28	1.02106E-10	1.02811E-08
C1orf107	204699_s_at	6.27	1.10862E-11	1.75184E-09
DCTPP1	218069_at	6.27	1.64792E-11	2.36483E-09
None	218119_at	6.25	4.36169E-07	9.86792E-06
SEC11C	223299_at	6.25	2.46576E-08	9.02928E-07
KPNA6	212102_s_at	6.25	7.27111E-08	2.21106E-06
P2RY10	1553856_s_at	6.25	5.14139E-07	1.13303E-05
TRA@	215540_at	6.24	1.31168E-09	8.23377E-08
C8orf41	219124_at	6.23	6.38206E-12	1.11127E-09
None	222010_at	6.22	9.53053E-09	4.11272E-07
XPO5	223055_s_at	6.21	3.82471E-10	3.03401E-08
None	227030_at	6.21	1.04406E-09	6.82321E-08
RIT1	209882_at	6.21	5.76893E-07	1.24523E-05
WDR74	221712_s_at	6.21	2.6138E-09	1.43628E-07

PLK3	204958_at	6.20	6.32874E-07	1.34483E-05
AP1S3	1555733_s_at	6.20	3.62613E-11	4.46528E-09
MTFMT	235689_at	6.20	1.24413E-10	1.21905E-08
POLR1C	209317_at	6.20	1.0386E-08	4.42471E-07
ASAH2B	229793_at	6.19	3.38434E-08	1.17317E-06
NOLC1	211951_at	6.19	1.60463E-12	3.58095E-10
ARG2	203946_s_at	6.19	2.78692E-08	9.94614E-07
CENPH	231772_x_at	6.19	6.36541E-07	1.35157E-05
GTF2A2	202678_at	6.19	1.90124E-07	4.94434E-06
METTL6	1553689_s_at	6.18	7.37717E-08	2.23957E-06
ACD	204617_s_at	6.18	6.66473E-10	4.73239E-08
GAR1	219110_at	6.16	2.34243E-08	8.66319E-07
MCM3	201555_at	6.16	8.79021E-11	9.01375E-09
MTERFD1	219363_s_at	6.15	1.10162E-07	3.13702E-06
MCM4	222037_at	6.14	3.4849E-08	1.1991E-06
EIF1AD	223682_s_at	6.14	1.47667E-08	5.85901E-07
GART	212379_at	6.14	5.62515E-12	1.00838E-09
LINS1	220121_at	6.13	1.07498E-07	3.06917E-06
SNRPF	203832_at	6.12	4.45274E-10	3.45324E-08
PFDN6	233588_x_at	6.12	3.41421E-10	2.76551E-08
PTRH2	218732_at	6.10	7.19479E-09	3.28086E-07
DIMT1L	217106_x_at	6.10	1.75108E-07	4.60954E-06
CDC20	202870_s_at	6.07	2.36994E-07	5.91674E-06
ABCF2	209247_s_at	6.06	9.71006E-12	1.58951E-09
MCM4	212141_at	6.06	1.16611E-08	4.85951E-07
C1orf77	209927_s_at	6.05	2.47484E-08	9.04491E-07
HEATR2	1554761_a_at	6.05	7.04968E-09	3.22545E-07
TRAF4	202871_at	6.05	1.24343E-07	3.4846E-06
WDHD1	204728_s_at	6.05	2.48262E-10	2.17179E-08
DKC1	216212_s_at	6.05	1.65594E-07	4.4122E-06
WDHD1	204727_at	6.04	3.74992E-13	1.10825E-10
SOCS4	1552792_at	6.04	1.1564E-12	2.70197E-10
DHFR	202533_s_at	6.03	5.66836E-09	2.69259E-07
None	234860_at	6.02	2.8226E-12	5.69466E-10
EPB41	207793_s_at	6.02	1.1718E-11	1.82012E-09

MGAT4A	1569136_at	6.01	2.83371E-11	3.64549E-09
EXOSC4	218695_at	6.01	1.61951E-07	4.33204E-06
CLPP	202799_at	6.00	2.69007E-10	2.30532E-08
BNIP1	37226_at	6.00	1.24675E-14	7.25171E-12
BNIP1	207829_s_at	5.99	2.18225E-10	1.95278E-08
SLC4A7	210286_s_at	5.99	8.66373E-11	8.92301E-09
YARS	212048_s_at	5.98	1.7003E-09	1.01268E-07
SLC39A14	1555434_a_at	5.98	5.58163E-09	2.66529E-07
PATL1	235235_s_at	5.97	9.21751E-08	2.70079E-06
TADA2A	210537_s_at	5.97	2.04192E-11	2.76861E-09
WDR76	205519_at	5.95	2.86105E-09	1.55033E-07
None	236075_s_at	5.94	1.73549E-08	6.69216E-07
RPA3	209507_at	5.94	9.2098E-09	4.00912E-07
TGS1	238346_s_at	5.94	6.79345E-07	1.42699E-05
DHODH	213632_at	5.93	1.28916E-08	5.25197E-07
None	232790_at	5.93	1.35008E-08	5.43162E-07
C18orf55	223181_at	5.93	4.2849E-07	9.7291E-06
SENP3	215113_s_at	5.92	1.92237E-09	1.11696E-07
GK3P	215966_x_at	5.91	2.11438E-08	7.91805E-07
NAA15	226998_at	5.91	3.18366E-08	1.11083E-06
GFPT1	202721_s_at	5.91	2.48232E-08	9.06617E-07
MCM10	222962_s_at	5.90	2.11258E-11	2.83102E-09
PHF19	227211_at	5.90	5.09249E-08	1.65463E-06
GNG2	1555766_a_at	5.89	2.21625E-13	7.29962E-11
ADSL	202144_s_at	5.89	1.4055E-09	8.71264E-08
HSPD1	243372_at	5.89	1.46978E-14	8.28455E-12
CCND2	231259_s_at	5.88	3.12595E-10	2.58174E-08
None	224233_s_at	5.88	1.9942E-11	2.73928E-09
SLC43A3	213113_s_at	5.88	8.41672E-09	3.71116E-07
GTPBP8	223486_at	5.87	7.49656E-08	2.26952E-06
NFKBID	230052_s_at	5.87	8.60706E-12	1.43037E-09
TPM3	224164_at	5.87	1.78427E-10	1.63409E-08
BLM	205733_at	5.86	3.31847E-08	1.15198E-06
UTP18	222038_s_at	5.86	2.68158E-10	2.30165E-08
WDR46	209196_at	5.85	2.71269E-09	1.48168E-07

SNRPG	205644_s_at	5.84	8.812E-09	3.85745E-07
OSGEP	209450_at	5.84	5.3714E-12	9.69245E-10
CLPB	221845_s_at	5.83	7.34017E-07	1.52885E-05
XPO4	218479_s_at	5.83	1.53792E-08	6.05805E-07
TRUB1	235447_at	5.82	3.53807E-10	2.84477E-08
ZFY	207246_at	5.82	1.9142E-09	1.11458E-07
PDS5A	213983_s_at	5.81	1.15922E-07	3.27963E-06
POLR2D	214144_at	5.81	3.97341E-09	2.03224E-07
PWP2	209336_at	5.81	7.16172E-12	1.22365E-09
KLHL5	220682_s_at	5.81	2.15621E-10	1.93264E-08
GALM	234974_at	5.80	2.69688E-09	1.47452E-07
NOM1	227161_at	5.80	2.42639E-07	6.04938E-06
NUDCD1	225438_at	5.80	1.56237E-09	9.49141E-08
CD5	206485_at	5.80	1.7845E-07	4.67725E-06
CDC45L	204126_s_at	5.79	1.10277E-07	3.13704E-06
KLHL7	220238_s_at	5.78	4.97698E-07	1.10258E-05
STRBP	223246_s_at	5.78	1.6931E-10	1.56898E-08
NASP	201970_s_at	5.77	6.49697E-08	2.0181E-06
APOL6	1557236_at	5.76	1.05617E-11	1.68848E-09
OSBPL3	209626_s_at	5.76	1.60676E-07	4.30634E-06
CCDC59	222792_s_at	5.76	8.63471E-08	2.55191E-06
EXOSC9	205061_s_at	5.75	3.07623E-09	1.63294E-07
MRPL52	226241_s_at	5.75	2.53003E-08	9.22195E-07
FLAD1	205661_s_at	5.74	1.09175E-08	4.60581E-07
HSP90AA1	211968_s_at	5.74	4.51858E-07	1.01618E-05
LSM10	225593_at	5.73	2.1121E-09	1.21174E-07
LOC729082	231808_at	5.73	4.5639E-07	1.02477E-05
PHF19	227212_s_at	5.73	2.92915E-09	1.57784E-07
UBASH3A	220418_at	5.73	6.566E-08	2.03167E-06
BUB1	215509_s_at	5.72	5.39388E-13	1.47455E-10
METTL11A	223368_s_at	5.72	5.20166E-10	3.90125E-08
SNX20	229045_at	5.71	2.42959E-07	6.0546E-06
None	1558522_at	5.71	9.67597E-16	9.44703E-13
